# How Do Trauma‐ and Violence‐Informed Care Approaches Underpin Bariatric Surgery Interventions for Type 2 Diabetes Mellitus Remission? A Scoping Review

**DOI:** 10.1111/obr.13980

**Published:** 2025-07-03

**Authors:** Michelle Greenway, Michelle Domjancic, Yixuan (Claire) Liu, Alegria Benzaquen, Megan Racey, Susan M. Jack, Diana Sherifali, Carly Whitmore

**Affiliations:** ^1^ School of Nursing McMaster University Hamilton ON Canada; ^2^ McMaster Evidence Review and Synthesis Team McMaster University Hamilton ON Canada; ^3^ Department of Health Research, Methods, Evidence, and Impact McMaster University Hamilton ON Canada; ^4^ Centre for Addiction and Mental Health Toronto ON Canada

**Keywords:** bariatric surgery, obesity, trauma and violence informed care, type 2 diabetes mellitus

## Abstract

For individuals living with obesity, bariatric surgery is an effective intervention for type 2 diabetes mellitus (T2DM) remission. Given the established relationships between trauma and obesity, and obesity and T2DM, there is a need to examine bariatric surgical practices from a trauma‐ and violence‐informed care (TVIC) perspective. The purpose of this scoping review was to explore and describe the extent to which the four TVIC principles—(1) understand trauma, violence, and its impact; (2) create emotionally and physically safe environments; (3) foster opportunities for choice, collaboration, and connection; and (4) use a strengths‐based and capacity‐building approach—have been integrated into bariatric surgery processes for T2DM remission.

Following the PRISMA‐ScR framework, we searched MEDLINE and EMBASE from inception to January 2024. Eligible studies included adults ≥ 18 years with T2DM undergoing bariatric surgery and reporting remission outcomes. Data were summarized narratively and charted using the TIDieR checklist.

Nineteen studies were included, described in 30 publications. Despite established associations between trauma, obesity, and chronic illness, none of the included studies collected demographic data on participants' history of trauma or violence. Among included studies, mental health exclusions were common, potentially limiting access for individuals with trauma‐related mental health challenges.

Our findings highlight the absence of reporting TVIC principles in bariatric surgery for T2DM remission, raising concerns about emotional safety, risks for retraumatization, and long‐term outcomes. Integrating the principles of TVIC throughout bariatric surgical care is essential to promote emotionally safe and inclusive care to enhance postoperative success and sustained health outcomes.

## 1 Introduction

Obesity is a complex health condition influenced by a range of societal, structural, environmental, and genetic factors that extend beyond individual choices and behaviors [1]. The prevalence of obesity has risen to pandemic levels, with the World Health Organization reporting that one in eight individuals worldwide is living with obesity [2]. Second only to nicotine use, obesity is a leading cause of preventable or premature deaths, including cancers, cardiovascular disease, and metabolic syndromes [3]. A significant health complication associated with obesity is type 2 diabetes mellitus (T2DM) [4]. T2DM is characterized by inadequate insulin secretion and insulin resistance in the liver, muscles, and adipose tissue [5]. Obesity is related to various mechanisms that contribute to insulin resistance, including inflammation, mitochondrial dysfunction, and hyperinsulinemia [6].

In 2021, an estimated 529 million people were living with T2DM, with this global burden projected to reach 1.31 billion people by 2050 [7]. The management and potential reversal of T2DM is an emerging priority for policymakers and public health officials, given the profound impacts this condition has on individuals and the expansive and costly implications for healthcare systems. While the traditional management of T2DM has focused on glycemic control, there has been a shift toward greater consideration of obesity‐related complications and incorporating weight loss strategies to achieve disease remission [6]. Diabetes remission definitions vary geographically, but an international consensus report defines it as glycated hemoglobin (HbA1c) < 6.5% (48 mmol/mol) measured at least 3 months after cessation of glucose‐lowering pharmacotherapy [8].

Since 2011, bariatric surgery has been acknowledged by the International Diabetes Foundation (IDF) as a viable treatment option for T2DM and obesity [4, 9]. To date, research findings have demonstrated that bariatric surgery is more effective in achieving diabetes remission compared to behavioral interventions such as diet and physical activity [10] and to medical therapy [11, 12]. However, in practice, there are several barriers for individuals to qualify and receive bariatric surgery to treat their diabetes. Depending on the country's regulations and bariatric surgery practices, there can be restrictive criteria to qualify for this surgery that do not align with populations who have T2DM, such as having a Body Mass Index (BMI) of > 40 kg/m^2^ or > 35 kg/m^2^ with obesity‐related complications such as T2DM, high blood pressure, or severe sleep apnea [13]. Further, access to bariatric surgery is limited and where available, often constrained by long wait times [14, 15]. Bariatric surgery carries inherent risks, such as surgical complications, nutrient deficiencies, and the potential for long‐term gastrointestinal issues, which must be carefully considered when weighing treatment options [16, 17]. Despite these barriers and risks, research continues to establish the effectiveness, safety, and longevity of bariatric surgery as a treatment for T2DM remission.

Known risk factors for obesity include genetic predisposition, environmental factors, and behavioral influences, yet for decades, obesity has often been viewed as a moral failing. Originally viewed as an energy imbalance stemming from poor lifestyle choices [18], the prevailing belief was that obesity could be corrected through “good” lifestyle choices, such as diet or exercise. However, in recent years, there has been a growing recognition that obesity is an outcome linked to a complex interplay of factors, and because of the groundbreaking work of Vincent Felitti, the link between obesity and the presence of a history of trauma or violence has been well‐established. In his seminal Adverse Childhood Experiences (ACE) Study, Felitti first identified the connection between an individual's history of sexual trauma and obesity, highlighting how past traumatic experiences can profoundly influence physical and mental health well into adulthood [19]. Reporting a graded relationship between the number of ACE exposures, Felitti identified that for those who experienced four or more categories of childhood exposure (e.g., psychological, physical, or sexual abuse, violence against mother, living with household members who use substances, are mentally ill, or ever imprisoned), compared to those who had experienced none, contributed to a 1.4‐ to 1.6‐fold increase in obesity [19]. Since this original work, several studies have reinforced these findings [18, 20], including in a meta‐analysis by Wiss and Brewerton, who found a 46% increased risk of obesity following exposure to ACEs [21]. Similarly, several studies reporting on candidates for bariatric surgery specifically have noted a positive association between lifetime traumatic experiences and eating habit dysfunction, such as binge‐eating disorder [1, 22].

While there is some evidence that has identified the utility and effectiveness of psychosocial interventions to help individuals prepare for and adjust to life after bariatric surgery, there is considerable variation and diversity in the types and timing of these interventions [23]. Given the established relationships between: (a) trauma, including experiences of interpersonal violence, and obesity; (b) obesity and T2DM; and (c) trauma and violence exposures and the presence of chronic conditions such as T2DM, there is a need to examine how trauma‐ and violence‐informed care (TVIC) approaches are integrated into comprehensive preoperative, operative, and postoperative assessment and support in bariatric surgery practices. Further, considering the known linkages between trauma and violence exposures and the presence of chronic conditions like T2DM—and that bariatric surgery is considered an effective approach for achieving diabetes remission in this population—the extent to which TVIC principles have been integrated into bariatric surgery processes for diabetes remission remains largely unknown.

TVIC builds on the foundational work of trauma‐informed care, which emerged from research on the lasting impacts of trauma on physical, emotional, and psychological health. Over time, this framing expanded to encompass the broader social and structural violence that individuals may experience throughout their lives, leading to the development of TVIC [24]. TVIC acknowledges that trauma is not just an individual experience but is often embedded within systemic and structural inequities. As a result, there is an opportunity for healthcare providers to create safe and collaborative environments within healthcare systems that honor individuals' histories and strengths, while providing optimal care for bariatric surgery that does not place individuals at risk for harm or retraumatization.

The extent to which TVIC principles have been integrated into bariatric surgery processes for diabetes remission remains largely unknown. In this scoping review, we sought to understand how TVIC approaches have been incorporated as strategies to minimize harm for those undergoing bariatric surgery for T2DM remission. This included an exploration of as follows: (a) who was eligible for these interventions; (b) the assessment and support offered or provided preoperatively, operatively, and postoperatively to address the needs of individuals living with a history of trauma and violence; (c) to identify gaps in the current evidence base; and (d) pose recommendations for further consideration.

## 2 Materials and Methods

This scoping review followed the Preferred Reporting Items for Systematic Reviews and Meta‐analyses for scoping reviews (PRISMA‐ScR) guidelines [25].

### 2.1 Search Strategy

The search terms, databases, and strategy were developed in consultation with a research librarian at McMaster University. We searched MEDLINE and EMBASE from inception to January 11, 2024. Search terms and strategy can be found in Appendix S1. We manually searched reference lists of similar and on‐topic systematic reviews for citations that were not captured in our search. Results from the search were deduplicated and uploaded to a secure internet‐based platform for screening (DistillerSR, Evidence Partners Inc., Ottawa, Canada).

### 2.2 Study Selection and Eligibility

To be included, studies had to be written in English, published in a peer‐reviewed journal, and meet the following criteria: (1) adults ≥ 18 years of age with T2D; (2) any bariatric surgery; (3) any study design that included a comparison group; and (4) diabetes remission outcomes. There were no criteria for diagnosis of T2DM or diabetes remission; however, studies with general adult populations or mixed populations that have subgroup analysis for participants with T2DM were also considered. Without subgroup analysis, a mixed population must have at least 80% of participants with T2D to be included in our review. Diabetes remission was defined by the authors of the included studies. Studies were excluded if: (1) they reported data on participants younger than 18 years of age who did not have T2DM or who were pregnant; (2) the primary intervention was not surgical; and (3) they were observational, cohorts, case studies, or chart reviews.

A team of researchers conducted the screening and data extraction. A minimum of two reviewers were required to independently and in duplicate screen titles and abstracts of all potentially eligible studies. Articles marked for inclusion by either team member went on to full‐text screening completed independently and in duplicate by two team members and required consensus for inclusion or exclusion. We used the DistillerSR AI tool to check for screening errors [26, 27]. This tool uses AI predictions against reviewed references to identify any that might have been erroneously excluded. Any inconsistencies in the included studies were discussed and resolved among the two reviewers and verified by a third reviewer.

### 2.3 Data Extraction and Data Charting

We developed, piloted, and deployed standardized forms for data extraction. For each included study, two researchers extracted study characteristics (e.g., country, setting, year, design, objective, sample size, recruitment, and duration of follow‐up), participant characteristics (e.g., age, sex/gender, ethnicity, socioeconomic status, and comorbidities), and intervention and comparison characteristics (e.g., inclusion/exclusion criteria, description, duration, dose, intensity, and setting of delivery). We also extracted diabetes remission definitions, outcomes, and results, which included how the study authors defined diabetes remission, what outcomes were assessed and measured, the diabetes remission rates postintervention with within and between group *p*‐values (when available), outcomes at other time points (if applicable), and any narrative reporting in the results and discussion.

To elicit information related to the description of surgical interventions and their implementation, we used the Template for Intervention Description and Replication (TIDieR) checklist tool [28]. TIDieR focuses on reporting details of the core and adaptable components of an intervention, and where relevant, comparison elements of a study.

There are four principles of TVIC: (1) understand trauma, violence, and its impact on people's lives and behavior; (2) create emotionally and physically safe environments for all patients and providers; (3) foster opportunities for choice, collaboration, and connection; and (4) use a strengths‐based and capacity‐building approach to support clients [24]. Aligned to the four principles of TVIC, we organized and extracted data that reported on preoperative screening or assessment, operative procedures, and postoperative support. See Table 1 for bariatric surgery‐specific examples of TVIC strategies at the individual and organization level.

**TABLE 1:** TVIC principles and bariatric surgery examples.

TVIC principle	Example (individual level)	Example (organization level)
Impact of trauma and violence on life and behavior	Be aware of potential “indicators” of a current/past history of trauma, including weight bias or bullying. Be prepared to ask about and listen without judgment to disclosures of trauma/abuse. Offer appropriate mental health referrals. Allow individuals to express and explore concerns about surgical processes and outcomes.	Staff training on the impact of trauma and violence on individuals' thoughts, feelings, actions, and health behaviors to foster sensitive, nonjudgmental care
Emotional and physically safe environments	Use language that is neutral and respectful, avoiding stigmatizing terms like “morbidly obese.” Foster a positive nonjudgmental attitude to reduce shame or stigma. Ensure individuals have access to bariatric‐appropriate seating, wheelchairs, and exam tables that are physically comfortable and accommodate their needs. Provide anticipatory guidance and clear expectations on what to expect before, during, and after the bariatric surgery.	Clinics that have private, calm waiting areas to ensure staff and individuals feel safe and respected. Incorporate trauma‐informed design principles, such as clear signage, privacy, and staff visibility
Opportunities for choice, collaboration, and connection	Offer choices between different treatment options or supportive care pathways. Empower the individual to make informed choices, emphasizing they have control over their healthcare decisions. Encourage individuals to bring a support person to appointments. Recognize the inherent power imbalances in the provider–patient relationship; engage the individual as an equal partner in their care plan to reduce feelings of helplessness.	Develop collaborative care teams that include dietitians, mental health professionals, and peer support workers to facilitate coordinated care and foster long‐term patient–provider relationships. Reimagine the use of time within the organization, so that providers have sufficient time to develop therapeutic alliances with individuals and address their concerns
Strengths‐based and capacity‐building approaches	Highlight the individual's strengths and past successes in managing their health, empowering them to see bariatric surgery as one tool among many they have to improve their health and quality of life. Acknowledge vulnerability, recognize that discussions of weight and surgery may evoke strong emotional responses. Approach these conversations with compassion, validating emotions rather than minimizing concerns. Be prepared to teach individual skills for navigating traumatic stress responses (e.g., grounding exercises, box breathing).	Implement programs that focus on capacity building, such as peer support groups or education workshops, share success stories to build community

All data were extracted in duplicate and verified by a third research team member.

### 2.4 Analysis

Extracted data were summarized and synthesized in the form of figures and tables. A narrative synthesis of the preoperative, operative, and postoperative assessment and support provided in the included studies is described. This includes a presentation of study, participant, and intervention characteristics as they relate to TVIC principles. Analytic interpretations were explored and discussed by all team members.

### 2.5 Ethical Considerations

As this study was solely literature‐based and did not involve any research participants or subjects, no formal ethics approval from the McMaster Research Ethics Board was required.

## 3 Results

**FIGURE 1:**
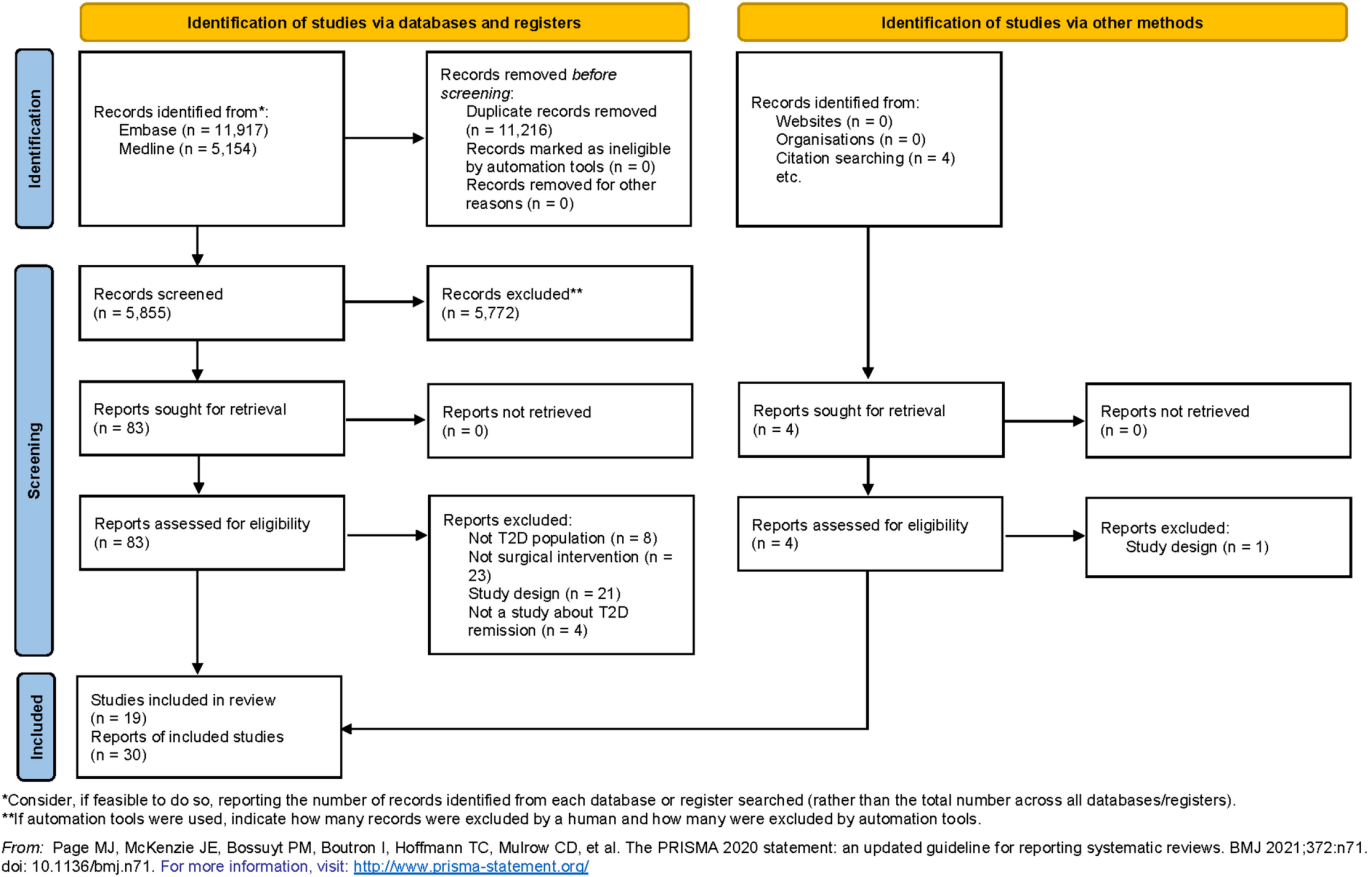
PRISMA 2020 flowchart.

We searched databases from inception and considered all studies based on the inclusion/exclusion criteria. Our search strategy yielded 5855 citations after duplicates were removed. Four additional records were identified from citation searching. After initial title and abstract screening and exclusion, we assessed 83 full‐text articles for eligibility. Nineteen eligible unique studies were included (Figure 1—PRISMA) described in 30 publications. Publications were grouped together if they were from the same initial study and provided information on various follow‐up periods. The studies were published from 2008 to 2023.

### 3.1 Characteristics of Studies

Study designs included 17 RCTs [29, 30, 31, 32, 33, 34, 35, 36, 37, 38, 39, 40, 41, 42, 43, 44, 45] and two prospective comparison studies [46, 47] (Table 2—Characteristics of Included Studies). Prospective studies were included if participants were assigned to a surgery or comparison group prior to the intervention. Six studies were conducted in Europe [32, 34, 37, 42, 46, 47], five in Asia [29, 35, 36, 41, 45], three in Oceania [38, 43, 44], three in North America [30, 31, 39], one in both Asia and North America [33], and one did not report the study location [40]. The median number of participants in a study was 60, with total sample sizes ranging from 24 to 127 participants. The duration of the study reporting ranged from 6 months (*n* = 1) [39] to 10 years (*n* = 1) [46], with the majority lasting 1 year (*n* = 10) [30, 31, 32, 34, 35, 36, 38, 42, 45, 47]. The remaining studies continued to measure outcomes for 2 years (*n* = 5) [33, 37, 41, 43, 44], 4 years (*n* = 1) [40], and 5 years (*n* = 1) [29].

**TABLE 2:** Characteristics of included studies.

Author, year	Country	Study design	Inclusion criteria	Sample size (N)	Age (mean years (SD))	Gender (female n (%))	BMI (mean (SD))	Duration of DM (years (SD))	Baseline A1C (% (SD))	Bariatric surgery Type(s)	Duration (years)
Cheng 2022 [29]	Singapore	RCT	T2D ≤ 10 years; age 21–65; BMI 27–32; HbA1c ≥ 8% despite primary care physician treatment; and one of the following: hypertension; hyperlipidemia; micro‐/macroalbuminuria; DM nephropathy; DM retinopathy.	N: 26 I: 12 C: 14	N: 44 (10) I: 40 (11) C: 48 (9)	I: 7 (58) C: 10 (71)	N: 29.4 (1.6) I: 29.1 (1.6) C: 29.7 (1.6)	N: N/R I: 5 (4–9 range) C: 6 (3–9 range)	N: N/R I: 9.9 (1.4) C: 9.3 (1.4)	RYGB GB	5
Courcoulas 2014 [30] Linked studies: Courcoulas 2015 [48]; Courcoulas 2020 [10]	United States of America	RCT	Age 25–55; BMI 30–40; T2D; treatment with anti‐DM meds and permission from treating physician for those with grade 1 obesity	N: 69 I: 24; 22 C: 23	N: 47.3 (6.4) I: 46.3 (7.2); 47.3 (7.0) C: 48.3 (4.7)	I: 19 (79); 18 (82) C: 19 (83)	N: 35.6 (3.0); 35.5 (3.4) I: 35.5 (2.6) C: 35.7 (3.3)	N: 6.4 (4.8) I: 7.4 (4.5); 6.1 (4.3) C: 5.7 (5.6)	N: 7.9 (2.0) I: 8.7 (2.2); 7.9 (2.2) C: 7.0 (0.76)	RYGB GB*	1
Cummings 2016 [31]	United States of America	RCT	Age 25–64; BMI 30–45; T2DM. DM medications; covered by insurance that had a bariatric surgery rider (if BMI 35–45 kg/m2); willing to accept randomization into either intervention group and full protocol for ≥ 1 year	N: 43 I: 23 C: 20	N: N/R I: 52.0 (8.3) C: 54.6 (6.3)	I: 12 (80) C: 10 (58.8)	N: 37.7 (N/R) I: 38.3 (3.7) C: 37.1 (3.5)	N: N/R I: 11.4 (4.8) C: 6.8 (5.2)	N: 7.5 (N/R) I: 7.7 (1.0) C: 7.3 (0.9)	RYGB GB	1
Dixon 2008 [44]	Australia	RCT	Age 20–60; BMI 30–40; T2D within 2 years; no evidence of renal impairment or DM retinopathy; able to understand and comply with study process	N: 60 I: 30 C: 30	N: N/R I: 46.6 (7.4) C: 47.1 (8.7)	I: 15 (50) C: 17 (57)	N: N/R I: 32 (2.7) C: 37.2 (2.5)	N: < 2 years I: N/R C: N/R	N: N/R I: 7.8 (1.2) C: 7.6 (1.4)	GB*	2
Fernandez‐Soto 2017 [47]	Spain	Prospective observational	N/R	N: 49 I: 12 C: 37	N: 49.0 (10.0) I: 51.0 (11.4) C: 48.4 (9.6)	I: 4 (8) C: 10 (20)	N: 50.2 (7.9) I: 47.7 (6.3) C: 51.0 (8.3)	N: 6.5 (2.5) I: 6.7 (2.8) C: 6.1 (2.3)	N: 7.4 (1.6) I: 8.3 (2.1) C: 7.1 (1.4)	RYGB SG BPD*	1
Hofsø 2019 [32] Linked study: Svanevik 2023 [49]	Norway	RCT (triple blind)	Age ≥ 18; BMI ≥ 33 with previous BMI ≥ 35; T2D; HbA1c ≥ 6.5% or use of antidiabetic meds with HbA1c ≥ 6.1%	N: 109 I: 55 C: 54	N: N/R I: 47.1 (10.2) C: 48.2 (8.9)	I: 32 (58) C: 40 (74)	N: N/R I: 42.1 (5.3) C: 42.4 (5.4)	N: N/R I: 6.3 (5.5) C: 6.6 (6.5)	N: NR I: 7.9 (range, 6.9–9.9) C: 7.6 (range, 6.8–8.5)	SG GB	1
Ikramuddin 2016 [33] Linked studies: Ikramuddin 2015 [50]; 2018 [51]; Chong 2017 [52]	Taiwan and United States of America	RCT	Age 30–67; T2D under medical care for at least 6 months before recruitment; HbA1c ≥ 8%; serum c‐peptide level > 1.0 ng/mL; BMI 30–39.9; willingness to accept randomization to either study group	N: 120 I: 60 C: 60	N: 49 (8) I: 49 (9) C: 49 (8)	I: 38 (63) C: 34 (57)	N: 34.6 (3.1) I: 34.9 (3.0) C: 34.3 (3.1)	N: 9 (95% CI, 7.9–10.0) I: 8.9 (6.1) C: 9.1 (5.6)	N: 9.6 (1.1) I: 9.6 (1.0) C: 9.6 (1.2)	RYGB	2
Kehagias 2023 [34]	Greece	RCT	Age 18–60; BMI ≥ 40; T2D; T2D < 8 years	N:24 I:12 C:12	N: 47 (11) I: 49.9 (9.8) C: 45.6 (10.7)	I: 6 (50%) C: 7 (58%)	N: 53 (11) I: 49.7 (7.8) C: 56.8 (11.5)	N: N/R I: 4.3 (2.4) C: 3.2 (1.9)	N: N/R I: 7.8 (1.7) C: 8.1 (1.7)	RYGB GB	1
Lee 2011 [35]	Taiwan	RCT	Age 30–60; BMI 25–35; diagnosis clear of poorly controlled HbA1c > 7.5%; T2D; treated by endocrinologist for ≥ 6 months; no renal impairment or DM retinopathy; able to understand and comply with study process	N: 60 I: 30 C: 30	N: 45 (range, 34–58) I: N/R C: N/R	N/R	N: 30.3 (range, 25–34) I: N/R C: N/R	N: N/R I: N/R C: N/R	N:10 (range, 7.5–15) N: N/R C: N/R	GB SG	1
Lin 2022 [36]	China	RCT	T2D; BMI 27.5–40; HbA1C > 7%; age 20–60; T2D diagnosis < 15 years; fasting C‐peptide > 370 pmol/L; and one of the following: BMI ≥ 32.5; BMI 27.5–32.5 with CV risk factors (high TG, low HDL, hypertension, or DM complications)	N: 96 I: 48 C: 48	N: N/R I: 36.9 (8.8) C: 36.9 (7.4)	I: 32 (67) C: 32 (67)	N: N/R I: 33.7 (3.5) C: 33.4 (3.5)	N: N/R I: 3.2 (2.7) C: 4.2 (4.0)	N: N/R I: 8.8 (1.2) C: 8.9 (1.3)	RYGB SG DJBSG	1
Mingrone 2012 [37] Linked studies: Mingrone 2015 [53]; 2021 [54]	Italy	RCT	Age 30–60; BMI ≥ 35; history T2D of 5 years; HbA1c ≥ 7.0%; and ability to understand/comply with study protocol	N: 60 I: 40 C: 20	N: N/R I: 42.8 (8.1); 43.9 (7.6) C: 43.5 (7.3)	I: 22 (55) C: 10 (50)	N: N/R I: 45.1 (7.8); 44.9 (5.2) C: 45.6 (6.2)	N: N/R I: 6.0 (1.3); 6.0 (1.2) C: 6.1 (1.2)	N: N/R I: 8.9 (1.7); 8.6 (1.4) C: 8.5 (1.2)	GB BPD*	2
Moriconi 2022 [46]	Italy	Prospective	Age 40–65; BMI ≥ 35; T2D according to ADA criteria; insulin‐taking patient with DM onset ≥ 40 years with negative presence of islet autoantibodies were considered to have T2D	N: 127 I: 96 C: 31	N: N/R I: 53 (8) C: 54 (7)	I: 67 (76) C: 12 (48)	N: N/R I: 46.7 (6.8) C: 45.5 (5.1)	N: N/R I: 2.5 (range, 1–10) C: 2 (range, 1–4.5)	N: N/R I: 7.9 (1.6) C: 8.0 (1.1)	RYGB	10
Murphy 2018 [38] Linked study: Pullman 2023 [55]	New Zealand	RCT	Age 25–50; BMI 35–65; T2D 6 months; suitable for surgical procedure and committed to follow up	N: 114 I: 56 C: 58	N: 47 (7) I: 46.6 (6.7) C: 45.5 (6.4)	I: 33 (59) C: 26 (45)	N: 43.0 (6.0) I: 42.2 (6.2) C: 1.9 (5.9)	N/R	N: 7.9 (1.4) I: 64.5 ± 18.1 mmol/mol C: 61.9 ± 12.8 mmol/mol	RYGB SG	1
Parikh 2014 [39]	United States of America	RCT	T2D; BMI 30–35; overweight for 5 years; failed to lose weight by nonsurgical means; the absence of medical or psychiatric contraindications; understanding of procedure and risks; strong motivation to comply with postsurgery regimen	N: 57 I: 29 C: 28	N: N/R I: 46.8 (8.1) C: 53.9 (8.4)	I: 23 (79) C: 22 (79)	N: N/R I: 32.8 (1.7) C: 32.4 (1.8)	N: N/R I: N/R C: N/R	N: 7.8(N/R) N: 7.7 (1.4) C: 7.9 (1.3)	RYGB SG GB GB*	0.5
Singh 2023 [40]	N/R	RCT	BMI ≥ 30; T2D	N: 49 I: 25 C: 24	N: N/R I: 45.8 (9.1) C: 46.6 (8.2)	I: 18 (72) C:16 (67)	N: N/R I: 47.0 (6.7) C: 44.7 (4.9)	N: N/R I: 3.4 (2.2) C: 4.6 (6.0)	N: N/R I: 7.9 (1.0) C: 8.3 (1.5)	RYGB GB	4
Techagumpuch 2019 [41]	Thailand	RCT	Age 15–60; BMI 32.5–60; T2D history < 10 years; history uncontrolled DM with medication treatment for > 6 months and HbA1c > 7% 1 week before surgery; ability to follow‐up for glycemic control at authors' hospital; and performance status safe for surgery	N: 104 I: 48 C: 56	N: 38.1 (N/R) I: 36.9 (10.8) C: 39.3 (8.8)	I: 21 (44) C: 24 (43)	N: 46.7 (N/R) I: 47.2 (5.4) C: 46.1 (6.6)	N: N/R I: N/R C: N/R	N: 9.0 (N/R) I: 9.0 (1.4) C: 9.0(1.5)	RYGB GB SG	2
Wallenius 2020 [42]	Sweden	RCT	T2D requiring anti‐DM medications; BMI 35–50; age 18–60.	N: 60 I: 29 C: 31	N: N/R I: 49.1 (9.2) C: 47.0 (10.7)	I: 12 (48) C: 11 (46)	N: N/R I: 39.5 (3.7) C: 40.8 (4.1)	N: N/R I: 5.5 (4.1) C: 5.0 (3.7)	N: NR I: 7.9 (1.5) C: 8.2 (1.9)	RYGB GB	1
Wentworth 2014 [43] Linked studies: Wentworth 2015 [56]; Qi 2023 [57]	Australia	RCT	Age 18–65; BMI 25–30; DM diagnosis < 5 years; willing to randomize to either study; ability to comply with treatment protocol	N: 51 I: 25 C: 26	N: N/R I: 53 (6) C: 53 (7)	I: 19 (76) C:17 (65)	N: N/R I: 29 (1) C: 29 (1)	N: N/R I: 2.2 (1.7) C: 2.8 (1.8)	N: N/R I: 6.9 (1.2) C: 7.2 (1.1)	GB*	2
Yi 2015 [45]	China	RCT	T2DM according to 1999 WHO diagnostic criteria, a ratio of the peak value to the base value of C peptide release test (CPRT) 42 (the base value of CPRT divided by an OGTT result 40.33 mg/L); BMI ≤ 35; age ≥ 18 years and ≤ 65 years	N: 60 I: 30 C: 30	N: N/R I: 49.1 (6.2) C: 48.2 (8.2)	I: 6 (20) C: 8 (27)	N: N/R I: 26.9 (0.7) C: 25.7 (0.9)	N: N/R I: 6.1 (4.7) C: 5.9 (4.5)	N: N/R I: 8.0 (1.5) C: 8.0 (1.2)	RYGB GB LRYGB‐SSP	1

Abbreviations: ADA, American Diabetes Association; BMI, body mass index (kg/m2); BPD*, biliopancreatic diversion; C, control; CV, cardiovascular; DJBSG, Duodenojejunal bypass with sleeve gastrectomy; DM, diabetes mellitus; GB, gastric bypass; GB*, gastric band; HbA1c, glycated hemoglobin; HDL, high‐density lipoprotein; I, intervention; LAGB, laparoscopic adjustable gastric banding; LRYGB, laproscopic Roux‐en‐y gastric bypass; LRYGB‐SSP, LRYGB with a small stomach pouch; N, overall/total; N/R, not reported; OGTT, oral glucose tolerance test; RCT, randomized controlled trial; RYGB, Roux‐en‐y gastric bypass; SG, sleeve gastrectomy; T1D, type 1 diabetes; T2D, type 2 diabetes; TG, triglycerides.

There were three main bariatric surgery techniques described in the included articles, including gastric bypass (*n* = 17) [29, 30, 31, 32, 33, 34, 35, 36, 37, 38, 39, 40, 41, 42, 45, 46, 47], sleeve gastrectomy (*n* = 8) [32, 35, 36, 38, 39, 47], and adjustable gastric banding (*n* = 4) [30, 39, 41, 42, 43, 44]. These surgical techniques were compared to each other (*n* = 11) [32, 34, 35, 36, 38, 40, 41, 42, 45, 46, 47] or to nonsurgical interventions such as lifestyle or medical management (*n* = 6) [29, 30, 31, 37, 39, 43, 46]. Two studies compared a combination intervention (surgical and nonsurgical) to nonsurgical [33, 44]. See Appendix S2 for more information on the included studies and the surgical procedures.

The studies in our review had variable diabetes remission definitions, with several providing thresholds in the absence of pharmacotherapy, such as fasting plasma glucose levels, glycated hemoglobin levels, and oral glucose tolerance tests (Table 3). All studies defined diabetes remission based on glycated hemoglobin levels below 5.7%, 6.0%, 6.5%, and 7.0%. Several studies required these levels without the use of hypoglycemic agents [29, 30, 31, 32, 33, 34, 35, 36, 37, 38, 39, 40, 42, 44, 45, 46]. In addition to the absence of these agents, specific durations of time postintervention were required, including assessment at 3 months [36], 6 months [34], 12 months [29, 41, 42, 46], and 24–36 months [33].

**TABLE 3:** Diabetes remission outcome results.

Author, year	Intervention description	Control description	Diabetes remission definition	Intervention diabetes remission (%)	Control diabetes remission (%)
Cheng 2022	Roux‐en‐Y gastric bypass (RYGB)	Best medical treatment	HbA1c ≤ 6% (≤ 42 mmol/mol) without use of glucose‐lowering meds 1‐year postintervention and beyond	42% at 4 years^3^	0% at 4 years
Courcoulas 2015	Roux‐en‐Y gastric bypass (RYGB)	Lifestyle weight loss intervention (LWLI)	Absence of meds with HbA1c < 5.7% and FPG ≤ 100 mg/dL per ADA guidelines	17% at 12 months^2^	0% at 12 months
	Laparoscopic adjustable gastric banding (LAGB)	Lifestyle weight loss intervention (LWLI)	Absence of meds with HbA1c < 5.7% and FPG ≤ 100 mg/dL per ADA guidelines	**23% at 12 months** ^**3**^	0% at 12 months
Cummings 2016	Roux‐en‐Y gastric bypass (RYGB)	Intensive lifestyle and medical intervention (ILMI)	HbA1c < 6.0% [< 42.1 mmol/mol] and off all diabetes medications	**60% at 1 year** ^**3**^	6% at one year
Dixon 2008	Laparoscopic adjustable gastric banding with conventional diabetes care	Conventional‐therapy program	FG < 126 mg/dL [7.0 mmol/L] and HbA1c < 6.2%, taking no glycemic therapy	**73% at 2 years** ^**3**^	13% at 2 years
Fernandez‐Soto 2017	Laparoscopy sleeve gastrectomy (LSG)	Biliopancreatic diversion (BPD) and Roux‐en‐Y gastric bypass (RYGB)	FPG < 100 mg/dL (5.6 mmol/L) and HbA1c < 6.0% (42 mmol/mol), suppressing the hypoglycemic treatment	83% at 1 year^2^	78% at 1 year
Hofsø 2019	Sleeve gastrectomy	Gastric bypass	HbA1c < /6.0% (42 mmol/mol) with no diabetes medications	47% at 1 year	**74% at 1 year** ^**3**^
Ikramuddin 2016	Lifestyle and Intensive Medical management and Roux‐en‐Y gastric bypass surgery (RYGB)	Lifestyle and intensive Medical management	HbA1c 6.0% at 24 and 36 months, with no use of antihyperglycemic meds from 24 to 36 months	**16% at 2 years** ^**3**^	0% at 2 years
Kehagias 2023	Laparoscopic Roux‐en‐Y gastric bypass with fundus resection (LYGBP + FR)	Laparoscopic Roux‐en‐Y gastric bypass (LRYGBP)	FG < 126 mg/dL, unimpaired glucose values after 120 min 75 g (OGTT), HbA1c < 6.5%, and no use of anti‐DM medications. Glycemic improvement and antidiabetic meds discontinued, without need for recommencing at 6 months	92% at 12 months^1^	100% at 12 months
Lee 2011	Gastric bypass with duodenum exclusion (GB)	Sleeve gastrectomy without duodenum exclusion (SG)	FPG glucose levels < 126 mg/dL and HbA1c < 6.5% without use of oral hypoglycemics or insulin	**93% at 12 months** ^**3**^	47% at 12 months
Lin 2022	Loop and duodenojejunal bypass with sleeve gastrectomy (Loop DJBSG)	Roux‐en‐Y duodenojejunal bypass with sleeve gastrectomy (Roux‐en‐Y DJBSG)	Return of HbA1C to < 6.5% (48 mmol/mol) postsurgery and persists for at least 3 months in the absence of usual glucose‐lowering medications	71% at 1 year^2^	65% at 1 year
Mingrone 2012	Roux‐en‐Y gastric bypass (RYGB)	Conventional medical treatment	HbA1c ≤ 6% (≤ 42·1 mmol/mol) and FPG ≤ 5·6 mmol/L at least 1 year without active pharmacologic therapy	**5% at 5 years** ^**3**^ (3 years after end of intervention)^a^	0% at 5 years
	Biliopancreatic diversion (BPD)	Conventional medical therapy	HbA1c ≤ 6% (≤ 421 mmol/mol) and FPG ≤ 5·6 mmol/L without glucose‐lowering drugs	**37% at 5 years** ^**3**^ (3 years after end of intervention)^a^	0% at five years
Moriconi 2022	Roux‐en‐Y gastric bypass (RYGB)	Medical therapy (MT)	FG < 100 mg/dL and HbA1c < 6.0% for 1 year in the absence of pharmacologic therapy or ongoing procedures	53% at 10 years^1^	0% at 10 years
Murphy 2018	Silastic ring laparoscopic Roux‐en‐Y gastric bypass (SR‐LRYGB)	Laparoscopic sleeve gastrectomy (LSG)	Four thresholds in the absence of pharmacotherapy including [≤ 38 mmol/mol (5.6%), < 42 mmol/mol (6.0%), < 48 mmol/mol (6.5%), or < 53 mmol/mol (7.0%)]	< 38 mmol (5.6%): 38% < 42 mmol (6.0): 52% < 48 mmol (6.5): 75% < 52 mmol (7.0): 80% at 1 year^2^	< 38 mmol (5.6%): 43% < 42 mmol (6.0): 49% < 48 mmol (6.5): 72% < 52 mmol (7.0): 77% at 1 year^2^
Parikh 2015	Surgery (bypass, band, or sleeve gastrectomy)	Intensive medical weight management (MWM)	No longer meeting the ADA criteria for T2D (FG > 126 mg/dL or glucose > /200 at 120 mins after 75‐g oral glucose load; or HbA1c > /6.5%), and without the use of diabetes medications	**65% at 6 months** ^**3**^	0% at 6 months
Singh 2023	One anastomosis gastric bypass (OAGB)	Roux‐en‐Y gastric bypass (RYGB)	Normal measures of glucose metabolism (HbA1c < 6%, FBG < 100 mg/dL) in the absence antidiabetic medications	72% at 4 years^2^	71% at 4 years
Techangumpuch 2019	Laparoscopic sleeve gastrectomy (LSG)	Laparoscopic Roux‐En‐Y gastric bypass (LRYGB)	FPG < 100, HbA1c < 6.0% without any diabetes medications for 1 year	67% at 2 years^2^	71% at 2 years
Wallenius 2022	Roux‐en‐Y gastric bypass (RYGB)	Sleeve gastrectomy (SG)	HbA1C < 6.0%, without diabetes medications 1 year after surgery	44% at 12 months^2^	46% at 12 months
Wentworth 2014	Laparoscopic adjustable gastric banding plus multidisciplinary diabetes care (LAGB)	Multidisciplinary diabetes care	Glucose concentrations < 7.0 mmol/L and < 11.1 mmol/L 2 h after PO glucose, at least 2 days after stopping glucose‐lowering drugs. Remission defined even when taking glucose‐lowering meds continuously until 2 days before this test	**52% at 2 years** ^**3**^	8% at 2 years
Yi 2015	Laparoscopic Roux‐Y gastric bypass with a small gastric pouch (LRYGB‐SSP)	Laparoscopic Roux‐Y gastric bypass (LRYGB)	Postop FBG < 7.0 mmol/L, 2‐h OGTT BG levels < 11.1 mmol/L, and HbA1c < 6.5% without DM meds	47% at 12 months^1^	30% at 12 months

1 = *p*‐value between groups not reported. 2 = *p*‐value between groups ≥ 0.05. 3 = *p*‐value between groups < 0.05. Also **bolded** to denote significance.

^a^ Complete remission rate at 5 years (3‐year follow‐up time point). Only partial remission was provided at the end of the intervention (2 years).

Abbreviations: BG, blood glucose; FG, fasting glucose; FPG, fasting plasma glucose; OGTT, oral glucose tolerance test.

### 3.2 Who Received Bariatric Surgery for T2DM Remission

A description of intervention participants is presented to characterize who received bariatric surgery for T2DM remission and illustrate how TVIC principles underpinned this sample. This includes a summary of demographic data, a description of the preoperative assessments reported, and the study eligibility criteria set.

All studies included adults and reported mean age ranging between 38.1 and 49.0 years (Table 2—Characteristics of Included Studies). Sixty percent of participants in the included studies were women. The studies in our review reported mean BMIs for the total population that ranged between 29.4 and 53 kg/m2. Thirteen studies required participants to have a BMI > 30 kg/m2 [30, 31, 32, 33, 34, 37, 38, 39, 40, 41, 42, 44, 46]. Duration of T2DM for participants in our included studies ranged from less than 2 years to 6.5 years, and the average participant baseline A1C level ranged between 7.4% and 10%.

Across all of the studies reviewed, there were no demographic data collected on participants' history of trauma or violence, such as childhood maltreatment, interpersonal violence, or other adverse experiences. This included no reporting in any of the included studies of any assessment or capture of this history in either preoperative or study‐specific eligibility screening. However, 13 of the included studies (68%) set inclusion or exclusion criteria related to mental health status and the presence of mental illness—a potential outcome of exposure to or experience of trauma. This included studies that excluded individuals who lived with a history of or active substance use disorder (*n* = 8) [29, 30, 32, 35, 36, 42, 44, 45], cognitive dysfunction or mental impairment, including dementia (*n* = 5) [30, 31, 35, 39, 44], serious mental illness such as schizophrenia, psychosis, bipolar disorder, or major depression (*n* = 5) [31, 32, 34, 39, 43], and a more broad description of “unstable” or “uncontrolled” psychiatric illness (*n* = 4) [29, 36, 42, 45]. The rationale for these exclusions included that participants with mental illness may have a higher than‐average risk for complications or that there may be greater difficulty in maintaining the treatment plan or following prescribed postsurgical care [31]. Four studies did not endorse any exclusion criteria regarding mental health and addictions [37, 38, 41, 46], with three additional studies not reporting any exclusion criteria altogether [33, 40, 47]. Beyond these exclusion criteria, mental health was not explicitly discussed in any of the included studies.

### 3.3 Intervention Implementation

For each of the included studies, a TIDieR template of reported intervention domains was completed (Table 4 and Appendix S3). Aligned with TVIC principles, the implementation of these bariatric surgical interventions and the postoperative assessment and support made available to participants is described.

**TABLE 4:** Template for intervention description and replication (TIDieR) checklist.

	Name	Why	What		Who	How	Where	When and how much	Tailoring	Modification	How well	
Author, year			Materials	Procedures							Planned	Actual
Cheng et al. 2020	X	X	N/R	X	X	N/R	X	X	N/R	N/R	N/R	X
Courcoulas et al. 2014	X	X	N/R	X	X	X	X	X	X	N/R	N/R	N/R
Cummings et al. 2016	X	X	N/R	X	X	X	N/R	X	N/R	N/R	N/R	N/R
Dixon et al. 2008	X	X	N/R	X	X	N/R	N/R	X	X	N/R	X	N/R
Fernandez‐Soto et al. 2017	X	X	N/R	X	X	N/R	X	X	N/R	N/R	N/R	N/R
Hofsø et al. 2019	X	X	N/R	X	X	X	X	X	X	X	X	X
Ikramuddin et al. 2016	X	X	X	X	X	X	X	X	X	X	X	X
Kehagias et al. 2023	X	X	N/R	X	X	N/R	X	X	N/R	N/R	N/R	N/R
Lee et al. 2011	X	X	N/R	X	X	N/R	X	X	N/R	N/R	X	N/R
Lin et al., 2022	X	X	N/R	X	N/R	N/R	X	X	X	N/R	N/R	N/R
Mingrone et al. 2012	X	X	N/R	X	X	N/R	X	X	X	X	N/R	X
Moriconi et al. 2022	X	X	N/R	X	X	X	X	X	X	N/R	N/R	N/R
Murphy et al., 2017	X	X	N/R	X	X	N/R	X	X	X	X	N/R	N/R
Parikh et al. 2014	X	X	X	X	X	N/R	X	X	X	N/R	N/R	N/R
Singh et al. 2023	X	X	N/R	X	X	N/R	X	X	N/R	N/R	N/R	N/R
Techagumpuch et al. 2019	X	X	N/R	X	X	N/R	X	X	N/R	N/R	N/R	N/R
Wallenius et al. 2020	X	X	N/R	X	X	N/R	X	X	N/R	X	N/R	N/R
Wentworth et al. 2014	X	X	N/R	X	X	X	X	X	X	X	X	X
Yi et al. 2017	X	X	N/R	X	X	N/R	X	X	N/R	N/R	N/R	N/R

Who delivered or administered these interventions were identified in all but three of the studies [36, 38, 47] and included physicians with specialization in endocrinology [33, 43], diabetology [29, 46], internal medicine [39], and psychiatry [46]; health professional team members including nutritionists and dietitians [29, 39, 43, 46], psychologists [29, 39, 46], diabetes educators [43], physiotherapists [29], and health educators [31]. Of these studies, 10 studies only described the engagement of a surgeon or surgical team in intervention delivery [30, 32, 34, 35, 37, 40, 41, 42, 44, 45]. While four of the studies did report multidisciplinary engagement or collaboration [29, 31, 39, 43, 46], it was not always clear what parts of the intervention components were delivered by these team members, how extensively or intensively these team members engaged in the interventions, how these team members collaborated with participants, or what their role may have been in building capacity postoperatively.

Only six studies reported on the mode of delivery of the intervention [30, 31, 32, 33, 43, 46], with all being in person, and one study offering additional telephone‐based appointments [31] and another offering additional multidisciplinary consultation as needed [43]. Tailoring and choice were offered to participants in some of the included studies, including variation in the frequency of clinical assessments [30], and the titration or adjustment of medications or nutritional advice respectively [32, 37, 43, 44]. Only one study provided participants an opportunity to choose the bariatric surgical procedure that they preferred [39]. This same study also permitted crossover from the nonsurgical control to the surgical intervention if the participant wished [39]. However, despite an opportunity for choice and collaboration in the implementation of these interventions, one of the included studies even blinded participants to the type of surgical intervention that they would receive [32].

Reporting of postsurgical support, education, and capacity building was limited. Only two of the 19 included studies reported the use of postoperative resources or educational materials [33, 39]. These included providing medication and nutritional supplementation recommendations or postoperative dietary guidelines [33, 39]. None of the included studies reported the provision of ongoing psychological counselling or support.

None of the studies reported serious adverse events related to the intervention or surgery, such as deaths or permanent disability. Several studies reported the need for additional surgery for treatment of infection or sepsis [29, 38], obstruction [29, 32, 33, 37, 42], ulcers [30], hernia [37, 43], cholecystectomy [38, 43], the replacement or repair of the initial bariatric procedure [30, 36], or for revisional bariatric procedure [30, 32, 42, 43, 44]. Other adverse events included ulcer [30, 45], pain [29], infection [32, 39, 41, 42, 44], anemia [29, 37], dehydration [29, 39], or hypoglycemia [31, 36, 37, 44], including diabetic ketoacidosis [33] treated medically. Two studies reported adverse mental health outcomes in the postoperative period, including one hospitalization for acute alcohol intoxication [31], one diagnosis of depression [33], and one suicide attempt [31, 33]. Two studies did not report adverse events [46, 47]; one reported the presence of no postoperative complications [34], with the other reporting nonspecific complications that required hospitalization [35].

## 4 Discussion

This review has identified 19 studies, described in 30 publications, that examined bariatric surgical interventions for T2DM remission. Despite well‐established associations between trauma, obesity, and chronic conditions like T2DM, none of the included studies reported data on participants' trauma histories, and many employed exclusion criteria targeting mental health conditions—a potential outcome of trauma—restricting access for those who could benefit from bariatric surgery under supportive conditions. This review has additionally identified that the focus of reported intervention components was predominantly clinical, limiting insights into whether preoperative, operative, and postoperative processes incorporated TVIC principles. The absence of these principles raises concerns about the potential for emotional harm, retraumatization, and the perpetuation of structural violence within care pathways. Embedding TVIC strategies throughout all surgical phases is essential to fostering emotionally safe, inclusive care environments and enhancing both immediate and long‐term patient outcomes.

Organized by surgical phase, we highlight opportunities to integrate and apply TVIC principles and pose key recommendations to improve the care, experience, and outcomes of bariatric surgery for T2DM remission through the integration of TVIC principles in preoperative, operative, and postoperative processes.

### 4.1 Preoperative Education and Assessment

It has been reported that 19.2% of individuals who seek bariatric surgery experienced childhood sexual abuse, and 22.1% reported childhood physical abuse [58]. The association between early exposure to trauma and violence and its impacts on long‐term physical and mental health, including increased risk of obesity and T2DM in later life, is well‐established. Given the well‐documented association between trauma, violence, obesity, and psychological and physical comorbidities, the presence of a history of trauma, including violence, should play a critical role in the pre‐ and postoperative treatment assessment, planning, and goal setting for those seeking bariatric surgery for T2DM remission. However, despite this relationship, our review has identified that beyond assessing for and subsequently excluding those who live with symptoms of mental health challenges or illness—a potential outcome of a history of trauma and violence—this history was not acknowledged, integrated, or addressed in bariatric surgical interventions for T2DM remission.

To deliver care that is safe, accessible, and does not retraumatize those who have been impacted or are experiencing trauma and violence requires that all members of the healthcare team incorporate an understanding of trauma and violence into their work [59, 60]. For this reason, it is recommended that all multidisciplinary staff delivering bariatric surgical interventions receive training in TVIC. This education should include foundational knowledge on trauma, violence, and their impacts on health, skills to identify signs of trauma and how to safely respond to disclosures, strategies for creating care environments that are experienced as emotionally, physically, and culturally safe, and to also recognize and respond to the unique trauma experiences of marginalized or racialized groups. Moreover, this education should bring an awareness of the links between the history of trauma, violence, obesity, and chronic conditions, so that staff may contribute to systems that place less blame on individuals and foster greater empathy for the complex reasons that individuals may struggle with their health and weight [60, 61, 62, 63]. While some of the included studies engaged multidisciplinary team members, no studies reported training staff for implementing these surgical interventions.

To better inform bariatric care pathways, ensure that the surgical interventions developed are inclusive of those with trauma histories and effectively address the unique needs of individuals undergoing bariatric surgical interventions. It is essential to collect comprehensive demographic data and thorough histories preoperatively [64]. This could include assessments of trauma and violence histories, psychosocial evaluations that assess motivation, social support, current stressors, weight history, symptoms of eating disorder, psychiatric history, and history of mental health treatment [65, 66]. Importantly, this assessment should evaluate for signs of post‐traumatic stress disorder, explore whether previous weight loss attempts have triggered trauma symptoms, and identify any social or structural barriers that may limit success [67]. Without understanding these underlying factors and incorporating them in care planning and goal setting, individuals may struggle with postoperative guidance, emotions, or self‐care activities, potentially diminishing the effectiveness of the intervention [68, 69]. Further, staff must be prepared for trauma and violence disclosures and trained on how to respond sensitively and effectively, ensuring that support, resources, and appropriate follow‐up are provided. None of the included studies in this review assessed for trauma or violence history, and only five of the included studies mentioned a psychological assessment prior to the intervention [29, 30, 35, 39, 46], with no further information provided on the assessment process, whether any mental health support was provided prior to the intervention, or how assessment findings may have tailored the care delivered.

### 4.2 Operative Processes

Research has shown that when individuals are actively involved in their healthcare decisions, they experience greater satisfaction, improved engagement in treatment plans, and better long‐term health outcomes [70]. For individuals with a history of trauma or violence, this involvement and collaboration help to create a sense of control and trust within the healthcare setting, reducing the risk of retraumatization and promoting emotional safety [24]. Healthcare services, like bariatric surgery for diabetes remission, can themselves unintentionally traumatize or retraumatize people during preoperative, operative, and postoperative processes [60]. However, the potential for these interventions to retraumatize those who have experienced trauma and violence was not explored in these interventions. Among the included studies, opportunities for input, choice, or collaboration were rare. Only one study allowed participants to select the bariatric surgical technique used and then offered the option to cross over from the control, once complete, to a surgical intervention [39]. Conversely, and antithetical to the TVIC principle of choice, collaboration, and connection, in one of the included studies, participants were blinded to the type of bariatric surgery that they would receive [32]. This lack of choice and collaboration is problematic, as different bariatric surgical techniques carry distinct risks, complications, and postsurgical care needs, which may impact long‐term outcomes, including diabetes remission [16], and may contribute to retraumatization.

To avoid this retraumatization, it is important to create both emotionally and physically safe environments as part of the bariatric surgical intervention process. This safety can be fostered by providing anticipatory guidance, ensuring that individuals are fully informed about what to expect throughout the preoperative, operative, and postoperative phases, and by offering clear expectations [24], including information about potential risks, complications, and long‐term outcomes of different surgical techniques. Patients can then actively participate in decision‐making with their healthcare team to align expert advice with their personal values and priorities. Moreover, creating and sustaining safe environments includes ensuring privacy, using nonstigmatizing language, and fostering a nonjudgmental clinical atmosphere. Modest actions like ensuring that individuals feel respected, using warm referrals to other services, and incorporating trauma‐sensitive approaches can reduce the potential for retraumatization, enhance the overall experience, and improve postoperative outcomes [60]. While the included studies discussed the ethical safety of study participants, this emotional and physical safety was not described.

### 4.3 Postoperative Care and Support

The evidence regarding the impact of bariatric surgery on postoperative outcomes and psychological well‐being is conflicting and poorly understood. Although some studies suggest a correlation between a history of trauma and worse postoperative outcomes, including reduced weight loss or diminished diabetes remission rates [71], others have found no significant relationship [64, 72, 73]. In some studies, it has been found that bariatric surgery may be beneficial to improving overall mental health, including reducing depression and anxiety symptoms and the presence of eating disorders after surgery [74, 75]; however, these improvements may only be in the short term, as evidence has also identified that these symptoms and conditions may return as early as 4 to 5 years following bariatric surgery [68, 69, 76, 77], with improvements peaking around 1 year after surgical intervention [68]. Specific to those who have reported a history of trauma or violence, several studies have reported a significant relationship with worsened depression symptoms, disordered eating, and heightened inpatient psychiatric hospitalization after bariatric surgery [58, 72, 78, 79, 80]. While this evidence is largely conflicting, the relationship between a risk of suicidal ideation and completion [81, 82, 83, 84, 85], self‐harm behavior [81, 86], and increased alcohol use disorder [87, 88] after bariatric surgery has been established.

Only two studies included in this review reported adverse mental health outcomes postoperatively, including hospitalization for one suicide attempt, a diagnosis of depression, and one participant with acute alcohol intoxication [31, 33]. Moreover, none of the included studies reported pre‐ or postoperative psychological counselling or support. Research has suggested that individuals with a history of trauma and violence may experience higher levels of distress in the first 5–9 months after bariatric surgery, but that these levels decrease by 10–14 months, highlighting the need for monitoring and psychological support within the first year after surgery [71, 83, 89]. Psychological support could include strengths‐based and capacity‐building interventions, such as motivational interviewing, where individuals can be encouraged to reflect on successes to identify the skills and abilities they can use to assist themselves post‐bariatric surgery [24]. Coupled with affirmations and structured goal setting, these strategies may enhance the ability of an individual to recognize their capacity for change and long‐term success. For individuals with trauma and violence histories, the risk for poor psychiatric comorbidities is already elevated, making it critical that surgical programs have comprehensive supports in place.

Included studies generally tracked diabetes remission outcomes for periods ranging from 6 months to several years, with most studies following participants for one to 2‐years post‐surgery. In studies with longer‐term follow‐up, our review identified a trend of unsustained diabetes remission, particularly 2 years post‐surgery. Although a decline in remission can result from multiple factors, such as baseline diabetes severity and duration and recurrent weight gain, one key factor may also be challenges linked to insufficient psychological support [46, 50, 90]. Our review highlights the need for more comprehensive, strengths‐based strategies to enhance long‐term postoperative success for individuals seeking bariatric surgery for diabetes remission.

## 5 Strengths and Limitations

Originally intended to be a systematic review (PROSPERO‐CRD42024524585), this review followed a systematic search, screening, and data extraction approach that was comprehensive, utilizing multiple databases and a methodological, rigorous process that involved duplication and verification during screening and data extraction. However, the identified literature did not support this type of review due to an overall lack of reporting of TVIC components and thus the inability to confirm or refute whether current practice considers TVIC in bariatric surgery for T2DM remission [91]. As a result, we pivoted to a scoping review to instead examine this emerging evidence and examine how this research was conducted [91]. No critical appraisal of the included literature was completed in this review; however, this is not a requirement of this review type [92]. Nonetheless, as the review aimed to explore the extent to which bariatric surgical interventions for T2DM remission are underpinned by TVIC principles, the use of the TIDieR tool served as a valuable way to examine intervention implementation details.

This review is limited by the reporting of intervention components within each of the included studies. While it could be said that the purpose of the included studies was not to assess or respond to the previous traumas of participants, given the body of literature on the importance of adverse childhood experiences and their association with obesity and diabetes risk in adulthood, we argue that TVIC principles should be a part of routine care for individuals considering bariatric surgery, including those who may investigate surgery as an option for diabetes remission.

## 6 Conclusion

This scoping review has highlighted the critical need for integrating TVIC principles into bariatric surgical interventions for T2DM remission. While bariatric surgery remains an effective treatment option for achieving diabetes remission, our findings suggest that the exclusion of TVIC principles in preoperative, operative, and postoperative bariatric surgical care not only fails to support the well‐being of the individual but also may contribute to increased psychological distress and poorer long‐term health outcomes, including the risk of retraumatization and reduced engagement in postsurgical care. Incorporating TVIC principles at the individual and organizational level has the potential to enhance both short‐ and long‐term outcomes by creating safer, more personalized, and supportive care environments. Moving forward, it is essential for healthcare professionals and systems to prioritize comprehensive care pathways that acknowledge the complex histories of those living with chronic conditions like T2DM and promote sustained health improvements post‐surgery.

## Author Contributions

All authors approved the protocol; Diana Sherifali and Megan Racey were responsible for overseeing the search of databases and literature. Megan Racey handled the management of the database and the deduplication of records. Megan Racey, Michelle Greenway, Michelle Domjancic, Yixuan (Claire) Liu, Alegria Benzaquen, Carly Whitmore, and Diana Sherifali were involved in the screening of citations; Megan Racey, Michelle Greenway, Michelle Domjancic, Yixuan (Claire) Liu, Alegria Benzaquen, and Carly Whitmore were responsible for data extraction, data verification, and analysis of data. All authors were involved in the interpretation of data and supported the drafting of the manuscript, with Michelle Greenway, Carly Whitmore, Megan Racey, Susan M. Jack, and Diana Sherifali leading the initial draft. All authors supported revising and formatting the manuscript. All authors have provided final approval of the version of the manuscript submitted for publication, and all authors agree to be accountable for all aspects of the work.

## Conflicts of Interest

The authors declare no conflicts of interest.

## Supporting information


**Data S1.** Search strategy.
**Data S2.** Characteristics of included studies.

## Data Availability

The main study data are the data extracted from the included studies, most of which are included in the manuscript tables. Any other supporting data relating to this review is available from the authors.
