# Supplementary material for: How Do Trauma‐ and Violence‐Informed Care Approaches Underpin Bariatric Surgery Interventions for Type 2 Diabetes Mellitus Remission? A Scoping Review
Source: Obes Rev. 2025 Jul 3;26(12):e13980. doi: 10.1111/obr.13980 (PMC12620105; doi:10.1111/obr.13980)
Supplement: Supplementary file 1 — Data S1. Search strategy. Data S2. Characteristics of included studies. [file OBR-26-e13980-s001.pdf]

How do trauma- and violence-informed care approaches underpin bariatric surgery interventions for type 2 diabetes mellitus remission? A scoping review

Michelle Greenway[1]
Michelle Domjancic[1]
Yixuan (Claire) Liu[1]
Alegria Benzaquen[1]
Megan Racey[1,2]
Susan M. Jack[1,3]
Diana Sherifali[1,2]
Carly Whitmore[1,4]

1. School of Nursing, McMaster University – Hamilton ON Canada
2. McMaster Evidence Review and Synthesis Team, McMaster University – Hamilton ON Canada
3. Department of Health Research, Methods, Evidence, and Impact, McMaster University – Hamilton ON Canada
4. Centre for Addiction and Mental Health – Toronto ON Canada

Corresponding Author:

Michelle Greenway
1280 Main St W, Hamilton ON L8S 4L8
m.greenway@mcmaster.ca 905-630-6014

Supplementary File 1: Search Strategy

Database: Ovid MEDLINE(R) ALL <1946 to January 09, 2024>

Search Strategy:

1 ((remission or recurrence* or relaps* or cure* or recurrent or revers*) adj5 diabet*).ti,ab,kf,kw. (9021)

2 Remission Induction/ (44729)

3 Recurrence/ (200115)

4 2 or 3 (239842)

5 diabetes mellitus/ or diabetes mellitus, type 1/ or diabetes mellitus, type 2/ (381876)

6 4 and 5 (2005)

7 1 or 6 (10279)

8 exp Bariatric Surgery/ (34357)

9 Bariatrics/ (516)

10 (bariatric* adj2 (surg* or operation* or procedure*)).ti,ab,kf. (26726)

11 (weight reduc* adj2 (surg* or procedure* or operation*)).ti,ab,kf. (343)

12 (obesity adj2 (surg* or operation* or procedure*)).ti,ab,kf. (3485)

13 (stomach* adj2 (band or banding or stapling)).ti,ab,kf. (43)

14 (gastr* adj2 (sleeve* or bypass* or band or banding)).ti,ab,kf. (23242)

15 ((intestinal or ileo* or jejuno*) adj2 bypass*).ti,ab,kf. (1730)

16 (biliopancreatic adj2 bypass*).ti,ab,kf. (216)

17 gastroplasty.ti,ab,kf. (2311)

18 gastroplasties.ti,ab,kf. (80)

19 lipectom*.ti,ab,kf. (903)

20 adipectom*.ti,ab,kf. (7)

21 liposuction*.ti,ab,kf. (3954)

22 lipolysis.ti,ab,kf. (15958)

23 lipoplast*.ti,ab,kf. (375)

24 or/8-19 (50278)

25 7 and 24 (1607)

26 exp clinical study/ (1157063)

27 exp Clinical Studies as Topic/ (394711)

28 clinical trial.pt. (539277)

29 controlled clinical trial.pt. (95521)

30 experimental trial*.mp. (3817)

31 clinical trial*.mp. (1192838)
32 clinical stud*.mp. (186795)
33 clinical article*.mp. (809)
34 (random* or rct*).mp. (1737571)
35 randomized controlled trial.pt. (606505)
36 exp case-control studies/ (1472431)
37 clinical trial, phase I.pt. (25519)
38 clinical trial, phase II.pt. (40743)
39 clinical trial, phase III.pt. (22356)
40 clinical trial, phase IV.pt. (2463)
41 ((intervention* or longitudinal or prospective or retrospective or case control*) adj2 stud*).mp. (2575069)
42 (open adj2 (trial* or stud*)).mp. (32593)
43 Random Allocation/ (107059)
44 Single-Blind Method/ (33159)
45 Double-Blind Method/ (177161)
46 ((singl* or doubl* or tripl* or trebl*) adj2 (blind* or mask*)).mp. (274423)
47 cohort studies/ or follow-up studies/ or longitudinal studies/ or prospective studies/ or retrospective studies/ (2559668)
48 cohort stud*.mp. (553988)
49 ((experimental or quasiexperimental or quasi experimental) adj2 (stud* or trial*)).mp. (230677)
50 prevention stud*.mp. (4853)
51 (multicenter stud* or multicentre stud* or multi center stud* or multi centre stud*).mp. (381385)
52 product surveillance, postmarketing/ or clinical trials, phase iv as topic/ (8021)
53 (postmarket* adj2 (stud* or trial* or surveill*)).mp. (9199)
54 follow up stud*.mp. (720627)
55 cohort analys?s.mp. (13058)
56 (non experimental stud* or nonexperimental stud*).mp. (475)
57 systematic review*.mp. (351484)
58 systematic reviews.pt. (0)
59 Cross-Sectional Studies/ (488769)
60 (cross sectional adj1 (stud* or survey* or design* or research*)).mp. (592778)
61 meta-analysis/ (192947)
62 meta analysis.pt. (192947)
63 meta analys?s.mp. (322788)

64 or/26-63 (6262192)

65 25 and 64 (994)

66 65 not (animals/ not (humans/ and animals/)) (962)

67 remove duplicates from 66 (960)

Database: Ovid MEDLINE(R) ALL <1946 to January 09, 2024>

Search Strategy:

1 ((remission or recurrence* or relaps* or cure* or recurrent or revers*) adj5 diabet*).ti,ab,kw. (8943)

2 remission/ (0)

3 recurrent disease/ (0)

4 2 or 3 (0)

5 diabetes mellitus/ or insulin dependent diabetes mellitus/ or non insulin dependent diabetes mellitus/ (381876)

6 4 and 5 (0)

7 1 or 6 (8943)

8 exp bariatric surgery/ (34357)

9 bariatrics/ (516)

10 (bariatric* adj2 (surg* or operation* or procedure*)).ti,ab,kw. (25091)

11 (weight reduc* adj2 (surg* or procedure* or operation*)).ti,ab,kw. (343)

12 (obesity adj2 (surg* or operation* or procedure*)).ti,ab,kw. (3631)

13 (stomach* adj2 (band or banding or stapling)).ti,ab,kw. (40)

14 (gastr* adj2 (sleeve* or bypass* or band or banding)).ti,ab,kw. (22791)

15 ((intestinal or ileo* or jejuno*) adj2 bypass*).ti,ab,kw. (1719)

16 (biliopancreatic adj2 bypass*).ti,ab,kw. (213)

17 gastroplasties.ti,ab,kw. (80)

18 gastroplasty.ti,ab,kw. (2280)

19 lipectomy/ (4106)

20 lipectom*.ti,ab,kw. (888)

21 adipectom*.ti,ab,kw. (7)

22 liposuction/ (4106)

23 liposuction*.ti,ab,kw. (3944)

24 lipolysis/ (8563)

25 lipolysis.ti,ab,kw. (15894)

26 lipoplasty/ (4106)

27 lipoplast*.ti,ab,kw. (372)

28 8 or 9 or 10 or 11 or 12 or 13 or 14 or 15 or 16 or 17 or 18 or 19 or 20 or 21 or 22 or 23 or 24 or 25 or 26 or 27 (69924)

29 7 and 28 (1476)

30 clinical study/ or exp case control study/ or clinical article/ or exp clinical trial/ or exp "clinical trial (topic)"/ or intervention study/ or longitudinal study/ or major clinical study/ or open study/ or exp postmarketing surveillance/ or prospective study/ or retrospective study/ (3022242)

31 clinical trial*.mp. (1192838)

32 clinical stud*.mp. (186795)

33 clinical article*.mp. (809)

34 ((intervention* or longitudinal or retrospective or prospective or case control) adj2 stud*).mp. (2574071)

35 (open adj2 (stud* or trial*)).mp. (32593)

36 (postmarket* adj2 (stud* or trial* or surveill*)).mp. (9199)

37 ((quasi experimental or quasiexperimental or experimental) adj2 (trial* or stud*)).mp. (230677)

38 quasi experimental study/ (1067)

39 experimental study/ (0)

40 randomized controlled trial/ (606505)

41 ((multicenter or multicentre) adj2 (stud* or trial*)).mp. (395845)

42 (rct* or random*).mp. (1737571)

43 follow-up stud*.mp. (720627)

44 exp randomization/ (107059)

45 single blind procedure/ (0)

46 double blind procedure/ (0)

47 ((singl* or doubl* or tripl* or trebl*) adj2 (blind* or mask*)).mp. (274423)

48 cohort analysis/ (336448)

49 cohort stud*.mp. (553988)

50 cohort analys?s.mp. (13058)

51 observational study/ (150775)

52 nonexperimental stud*.mp. (226)

53 non experimental stud*.mp. (250)

54 observational stud*.mp. (263886)

55 "systematic review"/ (249301)

56 systematic review*.mp. (351484)

57 meta analysis/ (192947)

58 meta analys?s.mp. (322788)

59 cross-sectional study/ (488769)

60 (cross sectional adj1 (stud* or survey* or design* or research*)).mp. (592778)

61 prevention stud*.mp. (4853)

62 or/30-61 (6285323)

63 29 and 62 (904)

64 remove duplicates from 63 (902)

65 64 not (animals/ not (humans/ and animals/)) (872)

Supplemental File 2 - Characteristics of Included Studies

**Cheng et al., 2022**	
Country, Year	Singapore, 2022
Question/Study Objective	This randomized controlled trial aimed to assess the durability of metabolic control and weight loss conferred by RYGB versus best medical treatment (including treatment with GLP1RA and SGLT2i) in a multi-ethnic Asian cohort with T2DM and BMI between 27–32 kg/m2 for up to 5 years. Continuous glucose monitoring (CGM) was performed to characterize glucose profiles and glycemic variability over time. In addition, secondary metabolic endpoints, healthcare costs and adverse events were evaluated.
Study Design	RCT.
Inclusion/Exclusion Criteria	Inclusion criteria:<br>Patients with established diagnosis of T2DM of duration ≤ 10 years, aged 21–65 years, BMI 27–32 kg/m2, HbA1c ≥ 8% (≥64 mmol/mol) despite treatment by the primary care physician, and at least one of the following co-morbidities on treatment: hypertension, hyperlipidaemia, micro/macroalbuminuria, diabetic nephropathy, or diabetic retinopathy.<br><br>Exclusion criteria:<br>A history of bariatric surgery or extensive upper abdominal surgery, pregnancy, nephropathy requiring dialysis, unfit for general anaesthesia or surgery, unwilling or possibly unable to adhere to the follow-up process, reluctant to be randomized into the two study groups, unstable psychiatric illness, or active substance abuse.<br><br>All recruited subjects were tested anti-glutamic acid decarboxylase anti-body negative and had c-peptide levels of > 300 pg/ml.
Diabetes Remission Definition	Complete diabetes remission was defined as HbA1c ≤ 6% (≤42 mmol/mol) without the use of glucose-lowering medication at 12 months post-intervention and beyond.
Sample Size	N=26<br>I=12<br>C=14
Loss to Follow-Up	N=13(50%)<br>I=6(50%)<br>C=7(50%)
Age	Mean overall (SD)= 44(10)<br>Mean I (SD)= 40(11)<br>Mean C (SD)= 48(9)
Gender	Male: I: 5(41.7%); C: 4(28.6%)
Race/Ethnicity	Chinese, Malay, and Indian
BMI	Mean overall (SD)= 29.4(1.6)<br>Mean I (SD)= 29.1(1.6)<br>Mean C (SD)= 29.7(1.6)
Comorbidities	The inclusion criteria specified the following co-morbidities: hypertension, hyperlipidaemia, micro/macroalbuminuria, diabetic nephropathy, or diabetic retinopathy.
Duration of Diabetes	Mean overall (years SD)= N/R<br>Mean I (years SD)= 5(4-9 range)

	Mean C (years SD)= 6(3-9 range)
Baseline A1C%	Mean overall (SD)= N/R<br>Mean I (SD)= 9.9(1.4)<br>Mean C (SD)= 9.3(1.4)
Description of Intervention	Initial preoperative workup included, but was not limited to, consultation with surgeon, diabetologist, dietitian, physiotherapist, and psychologist; blood and urine tests, upper endoscopy, colonoscopy for over 50 years of age or otherwise indicated, sleep study, and abdominal ultrasound.<br><br>Subjects underwent a standard laparoscopic RYGB with a 30-ml gastric pouch size, 10 mm gastrojejunostomy, 50-cm biliopancreatic limb, and 100-cm alimentary limb.<br><br>Gastroenterostomy and jejunojejunostomy were performed using linear staples plus handsewn closure of enterostomy. Petersen's space and jejunal mesenteric defect were closed with non-absorbable sutures. A leak test was performed with air insufflation through an endoscope.<br><br>Inpatient stay was usually 2 nights. On the first postoperative day, a barium swallow was performed. Oral fluid was permitted as soon as possible in small quantity until a normal barium swallow was reported. Oral medication was then reintroduced after adequate oral fluid was tolerated. In addition, visits by diabetologist, dietitian, and physiotherapist was conducted on day 1 post-surgery. Subjects were discharged the morning of post-operative day 2. Study visits were scheduled at week 2 and week 6 after surgery, then at 3, 6, 9, 12 months, then at 6-months intervals until the program ends. Each visit includes surgeon and diabetologist consult. Dietary and lifestyle intervention were more regular initially. Other visits were scheduled according to needs, with referral to other services as indicated.
Category of Intervention	RYGB<br>Gastric bypass
Who Delivered Intervention	Surgeon, diabetologist, dietitian, physiotherapist, and psychologist.
Location/Site of Delivery	Hospital.
Description of Control	All participants were assessed by a dietitian, endocrinologist, diabetes nurse educator, and physiotherapist. Initial consultation with diabetologist after randomization involved adjustment to existing glucose-lowering medications, including introduction of newer classes of glucose-lowering drugs (including GLP1RA and SGLT2i). An initial assessment of diet, individualized meal planning and counselling on best dietary practices based on the latest Singapore Ministry of Health Clinical Practice Guidelines for Management of Diabetes Mellitus and advice to engage in moderate intensity physical activity (minimum of 150 min/week) if deemed medically fit to do so, were also provided in the initial consultation. Clinic visits with diabetologists were scheduled every 3 months until the end of the program. Dietary and lifestyle interventions were more frequent during the first year. Other visits and consultations were scheduled as indicated.
Duration of Intervention and control	I: 5 years<br>C: 5 years

Length of Follow-Up Beyond Post-Intervention	N/A
List of Outcomes	1. Weight<br>2. BMI<br>3. Waist circumference<br>4. FPG<br>5. HbA1c<br>6. SBP, DBP<br>7. Total cholesterol, HDL-C, LDL-C, TG<br><br>Other two primary outcomes: hypertension and hyperlipidemia remission.
Serious Adverse Events	There were three postoperative complications needing reinterventions including a re-laparoscopy at 6 weeks for intra-peritoneal sepsis, and two upper endoscopy for bezoar gastrojejunostomy obstruction. One subject was admitted for postoperative dehydration. Two post-surgery defaulters, sans supplements, required transfusion from anaemia. Two other surgical subjects had non-specific abdominal pain, and another had dumping syndrome. A patient who had stage 3 chronic kidney disease (CKD) on randomisation required dialysis at year 5 post-surgery despite achieving prolonged diabetes remission. In the medical group, one subject developed intolerance to GLP1RA requiring discontinuation. Five subjects from the RYGB arm and 13 subjects from the medical treatment arm experienced no complications throughout the study period.
Funding Source	Alexandra Health Small Innovative Grant [SIGII/13001]; Alexandra Health Enabling Grant [AHEG1705]; Alexandra Health Science Translational and Applied Research Grant [STAR18110]; and Medtronic Grant [ERP-2018-11163]. SC Lim is supported by the Singapore Ministry of Health’s National Medical Research Council Clinical Scientist Award [NMRC/CSA-INV/0020/2017].
Comments	N/A

Courcoulas et al., 2014	
Country, Year	USA, 2014
Question/Study Objective	We report the results of a randomized, controlled clinical trial (RCT) examining the feasibility of a larger study and comparing the effectiveness of the two predominant types of bariatric surgery (RYGB, LAGB) and an intensive lifestyle intervention modeled after Look AHEAD19 in adults with grade 1 and 2 obesity and T2DM.
Study Design	RCT.
Inclusion/Exclusion Criteria	Inclusion criteria:<br>Adults were eligible for enrollment if they were between the ages of 25 and 55 years of age and had a BMI between 30 and 40 kg/m2, as this is a high priority subgroup for comparative effectiveness studies.11 T2DM was confirmed by either a documented fasting plasma glucose (FPG) ≥126 mg/dL and/or treatment with an anti-diabetic medication, to include a broad spectrum of T2DM severity. For those with grade 1 obesity, treatment with an anti- diabetic medication and permission from their treating physician were required to participate.<br><br>Exclusion criteria:<br>Adults were excluded for prior weight loss surgery, impaired mental status, drug / alcohol addiction, current smoking, pregnancy or planned pregnancy, inability to tolerate general anesthesia due to poor health, Type I diabetes mellitus, failed nutrition or psychological assessment, unwillingness to be randomized, inability to provide informed consent, or if they were deemed unlikely to comply with study visits or procedures.
Diabetes Remission Definition	Complete remission of T2DM was defined as absence of medications with glycated hemoglobin <5.7% and FPG ≤ 100 mg/dL per ADA guidelines.
Sample Size	N=69<br>I=24<br>C=23
Loss to Follow-Up	N=5(7%)<br>I=2(8%)<br>C=1(4%)
Age	Mean overall (SD)= 47.3(6.4)<br>Mean I (SD)= 46.3(7.2)<br>Mean C (SD)= 48.3(4.7)
Gender	Female: N/R<br>Male: I: 5(20.8); C: 4(17.4)
Race/Ethnicity	African American Race<br>Intervention 1 RYGB: 8(33.3)<br>Intervention 2 LAGB: 3(13.6)<br>Control: 4(17.4)
BMI	Mean overall (SD)= 35.6 (3.0)<br>Mean I (SD)= 35.5(2.6)<br>Mean C (SD)= 35.7 (3.3)
Comorbidities	Dyslipidemia<br>Hypercholesterolemia<br>Hypertension

Duration of Diabetes	Mean overall (years SD)= 6.4(4.8)<br>Mean I (years SD)= 7.4(4.5)<br>Mean C (years SD)= 5.7 (5.6)
Baseline A1C%	Mean overall (SD)= 7.9(2.0)<br>Mean I (SD)= 8.7 (2.2)<br>Mean C (SD)= 7.0 (0.76)
Description of Intervention	The RYGB was performed with a standard retrocolic, retrogastric technique using a linear stapled and hand sewn gastrojejunal anastomosis. Surgical participants underwent clinical follow-up assessments consistent with current practice: RYGB at 2 weeks, 3 months, 6 months, 9 months, and 12 months post-operatively. Those undergoing surgical intervention were counseled on a diet program consistent with post-bariatric surgery recommendations and were encouraged to exercise a minimum of 3–4 times per week and to focus on weight- bearing, aerobic activity.
Category of Intervention	RYGB<br>Gastric band
Who Delivered Intervention	Surgeon.
Location/Site of Delivery	Academic medical centre.
Description of Control	Subjects randomized to undergo the LWLI (Lifestyle Weight Loss Intervention) underwent a standard behavioral weight control program delivered in an in-person, individual, format based on the intervention developed for the Diabetes Prevention Program (DPP)20 and the Look AHEAD Study and adapted into a 12-month program for subjects with grade 1–2 obesity. During the initial 6 months of treatment, LWLI participants attended weekly in-person intervention sessions. During months 7–12, they attended in person sessions on the 1st and 3rd week of the month and received brief telephone contacts on the 2nd and 4th weeks. Each session focused on a specific behavioral topic related to weight loss, eating or exercise behaviors. Participants were provided supplemental written materials and were asked to self-monitor body weight, eating, and exercise. All LWLI participants were prescribed an energy restricted diet (1200– 1800 kcal/day) and were provided meal plans, meal replacements, and calorie-counter books. Moderate-intensity, 5-day-per-week exercise was prescribed beginning at 20 minutes per day and gradually progressing to at least 60 minutes per day, bouts of activity encouraged to be >10 minutes in duration.
Duration of Intervention and control	I: 1 year<br>C: 1 year
Length of Follow-Up Beyond Post-Intervention	5 years
List of Outcomes	Primary outcome:<br>The feasibility of performing a randomized trial involving surgical and non-surgical treatments. Feasibility was assessed by recruitment, randomization and retention rates.<br><br>Secondary outcomes:<br>Effectiveness to induce weight loss and diabetes improvements. Changes in lipids and blood pressure are also reported. Weight and height were assessed using a

	digital scale (Tanita; TBF-300A®) and a standard stadiometer, and blood pressure was measured twice at each visit. Serum measures of glycated hemoglobin, 12-hour fasting plasma glucose (FPG), total cholesterol, triglycerides, HDL, and LDL were obtained. Assessed at 12 months were weight loss (change in weight (kg), percentage weight loss from baseline and change in BMI), glycemic control (change in FPG and glycated hemoglobin), medication usage (categorized; none, oral/other medications, insulin use), and partial and complete remission of T2DM according to the ADA 2009 definitions. Change in blood pressure and serum lipids were also assessed at 12 months. Co-morbid health conditions (dyslipidemia/hypercholesterolemia, hypertension) were evaluated using a standardized comorbidity status form at baseline and follow-up. Participants completed a treatment preference questionnaire (TPQ) prior to but independent of randomization at baseline and follow-up with the participants asked to rate from 1 to 6 (‘strongly prefer’ to ‘strongly do not prefer’) how they felt about each of the three treatments.
Serious Adverse Events	Six surgical subjects stayed one additional night following their operation for either nausea or glucose medication management. One LAGB patient required a second procedure to replace a port that later detached from its position on the muscle and 3 other surgical participants had mild symptoms requiring clinical attention. There were no deaths and 3 serious adverse events. One RYGB participant developed an ulcer that was treated medically and 2 LAGB subjects were hospitalized for dehydration.<br><br>**From RefID 1049*<br>*"For events and complications occurring more than 30 days after randomization, participants in the LWLI experienced primarily orthopedic related events with 10 individuals undergoing orthopedic procedures in the 5-year follow-up period. There were orthopedic events and procedures for participants in the RYGB (5) and LAGB (8) cohorts as well. There were no deaths in any of the groups, 1 cardiovascular event in the RYGB group requiring a coronary stent placement and 2 cardiovascular/blood pressure-related events (hypertension and hypotension) in LAGB participants. One participant in LWLI underwent a crossover bariatric surgical procedure to LAGB. One RYGB participant developed an anastomotic ulcer requiring an operation, and 2 LAGB participants underwent a revisional bariatric procedures. The complete list of 65 adverse events in 32 participants can be found in Table 3."*
Funding Source	The Triabetes Study was funded by NIH-NIDDK 1RC1DK086037-01 and by Magee Womens Hospital of UPMC (University of Pittsburgh Medical Center) for subsidizing the surgical procedures.
Comments	

**Cummings et al., 2016**	
Country, Year	USA, 2016
Question/Study Objective	We sought to address these issues in the CROSSROADS trial (Calorie Reduction or Surgery: Seeking to Reduce Obesity And Diabetes Study). By use of a population-based recruitment strategy to enrol patients with type 2 diabetes and a BMI of 30–45 kg/m2, we conducted a prospective RCT comparing RYGB to an intensive lifestyle and medical intervention (ILMI), including aggressive, supervised dieting and exercise, modelled after the Diabetes Prevention Program (DPP) [24] and LookAHEAD trials.
Study Design	Parallel-group RCT.
Inclusion/Exclusion Criteria	Inclusion criteria:<br>Candidates were considered eligible if they were 25–64 years old, had a BMI of 30–45 kg/m2, were currently taking diabetes medications, were covered by insurance that had a bariatric surgery rider (if BMI 35–45 kg/m2), and were willing to accept randomisation into either intervention group and then follow the full protocol for ≥1 year.<br><br>Exclusion criteria:<br>Candidates were considered ineligible if they had any of the following: pregnancy, cancer (except nonmelanoma skin cancer), ascites, peritoneal effusion, dementia, bipolar disorder, schizophrenia, cirrhosis, end-stage renal disease, human immunodeficiency virus, inflammatory bowel disease, diagnosed type 1 diabetes, diabetes secondary to a specific disease or glucocorticoid therapy, prior bariatric or major gastrointestinal surgery or organ trans- plantation. These exclusions were designed to eliminate patients who were at greater-than-average risk for complications, disease-related weight change or nonadherence to treatment and follow-up visits.
Diabetes Remission Definition	The primary endpoint of diabetes remission at one year (defined as an HbA1c <6.0% [<42.1 mmol/mol] off all diabetes medications).
Sample Size	N=43<br>I=23<br>C=20
Loss to Follow-Up	N=11<br>I=8(34.8%)<br>C=3(15%)
Age	Mean overall (SD)= N/R<br>Mean I (SD= 52.0(8.3)<br>Mean C (SD)= 54.6(6.3)
Gender	Female: I: 12(80%); C: 10(58.8)<br>Male: N/R
Race/Ethnicity	White ethnicity.
BMI	Mean overall (SD)= 37.7(N/R)<br>Mean I (SD)= 38.3(3.7)<br>Mean C (SD)= 37.1(3.5)
Comorbidities	Dyslipidemia<br>Hypertension
Duration of Diabetes	Mean overall (years SD)= N/R<br>Mean I (years SD)= 11.4(4.8)

	Mean C (years SD)= 6.8(5.2)
Baseline A1C%	Mean overall (SD)= 7.5(N/R)<br>Mean I (SD)= 7.7(1.0)<br>Mean C (SD)= 7.3(0.9)
Description of Intervention	Laparoscopic RYGB:<br>Participants randomised to surgery underwent a laparoscopic proximal RYGB, using an estimated 40 ml gastric pouch, 100–150 cm alimentary limb, a biliopancreatic limb that included 30–50 cm of jejunum beyond the ligament of Treitz, an antecolic/antegastric approach, and combined stapled and sutured technique. Surgical patients also underwent a 4-week pre-operative and 10-month postoperative behavioural treatment regimen. In the pre-operative phase, patients had weekly telephone-based appointments with a health educator and were required to attend 2–3 bariatric support group meetings. Patients continued to have phone appointments with their health educator for 10 months after surgery. The postoperative behavioural treatment programme focused on diet and nutrition counselling, behaviour modification and exercise recommendations.
Category of Intervention	RYGB<br>Gastric bypass
Who Delivered Intervention	Surgeon and health educator.
Location/Site of Delivery	N/R
Description of Control	The Intensive lifestyle and medical intervention (ILMI):<br>The ILMI was a 12-month, in-person and telephone-based programme that included behaviour modification skills counselling, combined with training in diet and exercise change.<br><br>Exercise intervention: The focus of the exercise intervention was a gradual increase in brisk walking or other activities of similar moderate aerobic intensity over 12 months. Participants were asked to attend ≥3 exercise physiologist-supervised sessions per week at the FHCRC Prevention Center Exercise Testing and Training Center, a dedicated research gym, and they were asked to exercise an additional ≥2 days/week at home for the first 6 months. For the remaining 6 months, par- ticipants were asked to exercise ≥1 day/week at the Prevention Center and ≥4 days/week at home. In summary, they were directed to exercise ≥45 min/day, ≥5 days/week, for 1 year.<br><br>Dietary intervention: The dietary intervention was conducted by a research dietitian trained in behaviour modification. Each participant was required to attend weekly group nutrition ses- sions for the first 6 months. These sessions were based on DPP [24], with several modifications for our diabetic participants. Although reduced calorie intake and weight loss were strongly encouraged, participants were not given specific weight loss goals. Instead, the dietary intervention emphasised food qual- ity by encouraging consumption of protein, fresh fruits and vegetables, and avoidance of processed foods. The pro- gramme advocated a slightly higher percentage of energy from protein and fat, combined with avoidance of high glycaemic index foods. In the second 6-month phase of the study, participants were contacted weekly by the dietitian via telephone or email, and were encouraged to attend monthly in- person group nutrition sessions.

	Diabetes-related medical care Medical care, including pharmaceutical diabetes treatment, was provided similarly in both groups by each participant's own primary care physician, based on guidelines of the American Diabetes Association (ADA) and European Association for the Study of Diabetes [26]. Study staff conducted quarterly chart reviews to ensure these guidelines were met. Hypertension and lipid-lowering medications were prescribed according to ADA guidelines using the following treatment goals: blood pressure ≤130/ 80 mmHg and LDL-cholesterol ≤2.6 mmol/l.
Duration of Intervention and control	I: 1 year<br>C: 1 year
Length of Follow-Up Beyond Post-Intervention	N/R
List of Outcomes	Primary outcome:<br>Percentage of participants in each group who achieved diabetes remission at 1 year, defined as an HbA1c of <6.0% (<42.1 mmol/mol), off all diabetes medications.<br><br>Secondary outcomes:<br>Changes in fasting glucose and insulin levels, estimated insulin sensitivity, body weight, waist circumference, body composition, blood pressure, plasma lipids, aerobic fitness, medication usage, quality of life and safety.
Serious Adverse Events	During the year of observation, there were no deaths or hospitalisations for serious surgical adverse events (venous thromboembolism, hospitalisation ≥30 days, re-intervention) in either group of enrolled participants. Overall, there were 64 adverse events in the ILMI group and 31 in the RYGB group. Notably, these included more hypoglycaemic events with ILMI than RYGB: 43 vs 16, respectively, including four severe hypoglycaemias in ILMI (i.e. blood glucose <2.2 mmol/l, or <3.3 mmol/l with neuroglycopenic symptoms) vs none after RYGB. The only other severe adverse event was an emergency room visit for acute alcohol intoxication in one RYGB patient. Other reported minor adverse events were very diverse and displayed no obvious differential patterns between groups, except that musculoskeletal complaints were reported in seven cases with ILMI vs two with RYGB.
Funding Source	This study was funded by the NIH National Institute for Diabetes Digestive and Kidney Diseases Grant #R01-DK089528, as well as by grants from the GHRI and Group Health Foundation. The SDM aid used in this study was provided by the Informed Medical Decisions Foundation.
Comments	

Dixon et al., 2008	
Country, Year	Australia, 2008
Question/Study Objective	To determine if surgically induced weight loss results in better glycemic control and less need for diabetes medications than conventional approaches to weight loss and diabetes control.
Study Design	Unblinded RCT.
Inclusion/Exclusion Criteria	Inclusion criteria:<br>Patients were eligible if they were aged between 20 and 60 years, had a body mass index of 30 to 40, had been diagnosed with clearly documented type 2 diabetes within the previous 2 years, had no evidence of renal impairment or diabetic retinopathy, and were able to understand and comply with the study process.<br><br>Exclusion criteria:<br>Candidates were excluded if they had a history of type 1 diabetes, diabetes secondary to a specific disease, or previous bariatric surgery; a history of medical problems such as mental impairment, drug or alcohol addiction, recent major vascular event, internal malignancy, or portal hypertension; or a contraindication for either study group. Participants were excluded if they did not attend 2 initial information visits.
Diabetes Remission Definition	Remission of type 2 diabetes (fasting glucose level <126mg/dL [7.0 mmol/L] and glycated hemoglobin [HbA1c] value <6.2% while taking no glycemic therapy).
Sample Size	N=60<br>I=30<br>C=30
Loss to Follow-Up	N=5(8.3)<br>I=1(3.3)<br>C=4(13.3)
Age	Mean overall (SD)= N/R<br>Mean I (SD)= 46.6 (7.4)<br>Mean C (SD)= 47.1 (8.7)
Gender	Female: I:15 (50%); C:17 (57%)<br>Male: I:15 (50%); C:13 (43%)
Race/Ethnicity	N/R
BMI	Mean overall (SD)= N/R<br>Mean I (SD)= 32(2.7)<br>Mean C (SD)= 37.2(2.5)
Comorbidities	Hypertension I: 28 (93%); C: 27 (90%)<br>Metabolic syndrome I: 29 (97%); C: 29 (97%)<br>Coronary artery disease I: 0; C: 1 (3%)
Duration of Diabetes	Mean overall (years SD)= <2 years<br>Mean I (years SD)= N/R<br>Mean C (years SD)= N/R
Baseline A1C%	Mean overall (SD)= N/R<br>Mean I (SD)= 7.8(1.2)<br>Mean C (SD)= 7.6(1.4)
Description of Intervention	The surgical group underwent placement of a laparoscopic adjustable gastric band via the pars flaccida technique by 1 of 2 experienced surgeons within 1 month of randomization.

Category of Intervention	Gastric band
Who Delivered Intervention	Bariatric surgical team.
Location/Site of Delivery	N/R
Description of Control	Conventional-Therapy Program:<br>This program delivered best available medical practice for the treatment, education, and follow-up of patients with type 2 diabetes. Patients had open access to a general physician,<br>dietitian, nurse, and diabetes educator and had visits with at least 1 team member every 6 weeks throughout the 2 years. Medical therapies, including pharmaceutical agents, were determined by an experienced diabetologist on an individual basis. Lifestyle modification programs were individually structured to reduce energy intake, to reduce intake of fat (<30%) and saturated fats, and to encourage intake of low glycemic index and high-fiber foods. Physical activity advice encouraged 10 000 steps per day and 200 minutes per week of structured activity, including moderate intensity aerobic activity and resistance exercise. Lifestyle was the primary approach to weight loss, but very low calorie diets and medications were discussed with all patients and used after consultation with the dietitian or general physician if the patient expressed a desire to use additional measures.
Duration of Intervention and control	I: 2 years<br>C: 2 years
Length of Follow-Up Beyond Post-Intervention	N/R
List of Outcomes	Primary outcomes:<br>The proportion of participants achieving remission (exceptional glycemic control) of type 2 diabetes, defined as fasting plasma glucose levels less than 126 mg/dL (to convert to mmol/L, multiply by 0.0555) in addition to HbA1c values less than 6.2%<br>without the use of oral hypoglycemics or insulin.<br><br>Secondary outcomes:<br>P change in HbA1C levels, weight, blood pressure, waist circumference, and levels of fasting lipids, including total cholesterol, triglycerides, and high-density lipoprotein cholesterol. Changes in medication use, changes in the proportion of participants with the metabolic syndrome as defined by the National Cholesterol Education Program Adult Treatment Panel III criteria,24 and changes in indirect measures of insulin resistance using the homeostatic model assessment method were assessed.
Serious Adverse Events	Surgical Group:<br>One patient developed a superficial wound infection over the access port site 2 weeks postplacement, which resolved with intravenous antibiotics. Two patients developed gastric pouch enlargement, both at 10 months after placement, and were treated with nonurgent laparoscopic revisional surgery to remove and replace the band. One patient experienced eating difficulties and persistent regurgitation with

	no saline in the band and no impedance of flow on contrast study. The band was removed 15 days after placement. Hospital stay for each revisional procedure was less than 1 day, and there were no complications. Other adverse events reported were postoperative febrile episodes in 1 patient. No cause was found, and the fever resolved. A minor hypoglycemic episode occurred in 1 patient and gastrointestinal tract intolerance to metformin in another.<br><br>Conventional-Therapy Group:<br>Two patients had minor gastrointestinal tract adverse effects, and 1 had persistent diarrhea with metformin. One patient developed vasculitic rash, possibly related to rosiglitazone. All problems resolved when the medications were discontinued. One patient had multiple hypoglycemic episodes, and another was admitted to hospital with angina and a transient cerebral ischemic episode. Two patients were intolerant of very low-calorie meal replacement.
Funding Source	This study was funded by Monash University, which has received an unrestricted grant from Allergan Health. The laparoscopic adjustable gastric bands (Allergan Health) and the laparoscopic ports (Applied Medical) were provided without charge by the manufacturers.
Comments	

**Fernandez-Soto et al., 2017**	
Country, Year	Spain, 2017
Question/Study Objective	In this work, we seek to find out the short- and mid- term effectiveness of one restrictive technique (LSG) in type 2 diabetes mellitus improvement, weight loss, and related co-morbidities progress, in comparison with two mixed procedures (RYGB and BPD), after one year of follow-up. Furthermore, we try to identify reliable clinical baseline predictors of the likelihood of type 2 diabetes mellitus remission after these different surgical procedures.
Study Design	Prospective and observational study.
Inclusion/Exclusion Criteria	N/R
Diabetes Remission Definition	Complete type 2 diabetes mellitus remission required fasting glucose levels in plasma under 100 mg/dL (5.6 mmol/L) and HbA1c levels under 6.0% (42 mmol/ /mol), suppressing the hypoglycemic treatment.
Sample Size	N=49<br>I=12<br>C=37
Loss to Follow-Up	N=0<br>I=0<br>C=0
Age	N=49.0(10.0)<br>I=51.0(11.4)<br>C=48.4(9.6)
Gender	Male I: 4(8%); C: 10(20%)
Race/Ethnicity	N/R
BMI	N=50.2(7.9)<br>I=47.7(6.3);<br>C=51.0(8.3)
Comorbidities	Hypertension<br>Hyperlipidemia<br>Obstructive sleep apnea<br>Gastroesophageal reflux
Duration of Diabetes	N=6.5(2.5)<br>I=6.7(2.8)<br>C=6.1(2.3)
Baseline A1C%	N=7.4(1.6)<br>I=8.3(2.1)<br>C=7.1(1.4)
Description of Intervention	Laparoscopic sleeve gastrectomy technique:<br>A 37-French tube was employed to calibrate the sleeve gastrectomy. The gastric section started 6 cm from the pylorus. Buttress material was applied (Duet®) to strengthen the staple line. Drainage was systematic.

Category of Intervention	RYGB<br>Sleeve gastrectomy<br>Biliopancreatic diversion Scopinaro technique
Who Delivered Intervention	N/R
Location/Site of Delivery	Hospital.
Description of Control	Biliopancreatic diversion Scopinaro technique:<br>At 2.5 m from the ileo-caecal valve, the small bowel was transected and its distal extreme was anastomosed to the remaining stomach. At 50 cm proximal to the ileo-caecal valve, the proximal end of the ileum was anastomosed to the bowel in an end-to-side fashion, incorporating the remaining small bowel conveying the biliopancreatic juice and excluded from food transit.<br><br>Roux-en-Y Gastric Bypass technique:<br>A small gastric pouch was fashioned (30 cc) and a 150-cm-long antecolic alimentary limb was carried out. A gastro-jejunal anastomosis was created using linear stapling in line with the Lonroth technique. A side-to-side jejuno-jejunal anastomosis was carried out, 30 cm from the angle of Treitz and using linear stapling. The mesenteric defect was sealed using non- absorbable stitches. Drainage was systematic.
Duration of Intervention and control	I= 1 year<br>C= 1 year
Length of Follow-Up Beyond Post-Intervention	N/A
List of Outcomes	1. Weight loss<br>2. Glycaemic control<br>3. Lipid profile<br>4. Co-morbidity resolution<br>5. Type 2 diabetes mellitus remission<br>6. Predictive baseline parameters
Serious Adverse Events	N/R
Funding Source	Francisco Javier Zamora Camacho was partly supported by a Ramón Areces Foundation postdoctoral fellowship and Juan de la Cierva-Formación postdoctoral fellowship by the Spanish government
Comments	

**Hofsø et al., 2019**	
Country, Year	Norway, 2019
Question/Study Objective	Comparing the effectiveness of various bariatric procedures on remission of type 2 diabetes Roux-en-Y gastric bypass and sleeve gastrectomy. The primary objectives were to compare the effects of gastric bypass and sleeve gastrectomy on glycaemic control and β-cell function in subjects with severe obesity and type 2 diabetes. Specifically, we aimed to compare the effects of the two most commonly performed bariatric procedures worldwide, first, on remission of diabetes and, second, on beta-cell function.
Study Design	Randomised, triple-blind, single-centre superiority trial.
Inclusion/Exclusion Criteria	Inclusion criteria:<br>Age ≥18 years, current BMI ≥33·0 kg/m2 with previously verified BMI ≥35·0 kg/m2, and type 2 diabetes (glycated haemoglobin ≥6·5% [48 mmol/mol] or use of anti-diabetic medications with glycated haemoglobin ≥6·1% [43 mmol/mol]).<br><br>Exclusion criteria:<br>Previous major abdominal surgery, cancer, severe medical conditions associated with increased risk of complications, drug or alcohol addiction, pregnancy, and severe gastro-oesophageal reflux disease (Los Angeles classification grade>B or Barrett's oesophagus), regular use of steroids, elevated esophageal pressure (DCI >5000 mmHg*sec*cm) and symptoms of dysphagia and/or painful swallowing, reduced compliance due to severe mental and psychiatric conditions.
Diabetes Remission Definition	Defined by having a glycated haemoglobin of 6.0% (42 mmol/mol) or less with no diabetes medication.
Sample Size	N=109<br>I=55<br>C=54
Loss to Follow-Up	N=2<br>I=1<br>C=1
Age	Mean overall (SD)= N/R<br>Mean I (SD= 47.1(10.2)<br>Mean C (SD)= 48.2(8.9)
Gender	Female: I: 32 (58); C: 40 (74)<br>Male: I: 23 (42); C: 14 (26)
Race/Ethnicity	White I: 53 (96%); C: 51 (94%) (self-reported)
BMI	Mean overall (SD)= N/R<br>Mean I (SD)= 42.1 (5.3)<br>Mean C (SD)= 42.4 (5.4)
Comorbidities	Ischaemic heart disease<br>Antihypertensive use<br>Use of lipid-lowering medication<br>Fatty liver disease<br>Obstructive sleep apnea
Duration of Diabetes	Mean overall (years SD)= N/R<br>Mean I (years SD)= 6.3 (5.5)<br>Mean C (years SD)= 6.6 (6.5)

Baseline A1C%	Mean overall (SD)= NR<br>Mean I (SD)= 7.9 (6.9-9.9 range)<br>Mean C (SD)= 7.6 (6.8-8.5 range)
Description of Intervention	Sleeve Gastrectomy:<br>The two intervention groups received identical pre- and post-operative treatment, including a low calorie diet (<1200 kcal/day) during the two weeks preceding surgery. Antidiabetic and<br>antihypertensive medication, statin therapy, the management of reflux disease and vitamin and mineral supplementations, were adjusted according to specific predefined algorithms.<br>Patients were informed about healthy diets and physical activity, and the medical treatment<br>was in accordance with international guidelines.<br><br>The surgical procedures were performed laparoscopically. During sleeve gastrectomy, the greater curvature was dissected free starting 4 to 5 cm from the pylorus and up to the angle of His, with a tubular sleeve created using a 35 Fr bougie. All procedures were performed<br>by at least one of four experienced bariatric surgeons, all of whom are certified specialists in<br>gastrointestinal surgery. After surgery, patients were assessed at 5 weeks, 16 weeks, 34 weeks and one year. The study is ongoing with annual visits at 2, 3, 4 and 5 years after the surgical procedure.
Category of Intervention	Sleeve gastrectomy<br>Gastric bypass
Who Delivered Intervention	All procedures were performed by at least one of four experienced bariatric surgeons, all of whom are certified specialists in gastrointestinal surgery.
Location/Site of Delivery	Vestfold Hospital Trust in Norway.
Description of Control	Gastric bypass was performed with a 25 ml gastric pouch, an alimentary limb of 120 cm and a biliopancreatic limb of 60 cm.
Duration of Intervention and control	I: 1 year<br>C: 1 year
Length of Follow-Up Beyond Post-Intervention	2, 3, 4 and 5 years after the surgical procedure.
List of Outcomes	Primary outcomes:<br>1. Proportion of participants with complete remission of type 2 diabetes (glycated haemoglobin 6.0% [42 mmol/mol] or less without the use of glucose lowering medication)<br>2. The DI, a measure of beta-cell function, both assessed 1 year after surgery.<br><br>Secondary outcomes:<br>1-year changes in glucose homeostasis, body weight, body composition, cardiovascular risk factors and energy balance.
Serious Adverse Events	Adverse events were assessed and registered at each visit, including surgical and medical complications, hypoglycaemic episodes, dumping, and vitamin and mineral deficiencies. Early complications occurring within six weeks after the

	surgical procedure, were graded according to the Contracted Accordion Classification system (grade I-IV).<br>At each visit patients were examined for complications and side effects since the previous visit.<br><br>The largest groups of adverse events included infectious (n=10 vs n=10), gastrointestinal (n=11 vs n=10), and cardiovascular (n=8 vs n=8) complications. One patient who underwent sleeve gastrectomy was converted to gastric bypass because of insufficient weight loss, whereas one gastric bypass patient was re-operated because of small bowel<br>obstruction. A total of four patients, two in each group, underwent cholecystectomy because of symptomatic gallbladder stones. There were no deaths. (From 3-year follow-up).
Funding Source	The study is organised and financed by the Morbid Obesity Centre, Vestfold Hospital Trust, Tønsberg, Norway.
Comments	

Ikramuddin et al., 2016	
Country, Year	Taiwan & USA, 2016
Question/Study Objective	The study was designed to provide 2 years of intense lifestyle and medical management in all subjects with three additional years of observation while on usual medical care, so the results at 3 years reflect the first year of usual medical care after the first 2 study years. We had hypothesized that addition of gastric bypass to intense lifestyle and medical management would substantially improve the achievement of the ADA composite end point of metabolic control of type 2 diabetes.
Study Design	RCT.
Inclusion/Exclusion Criteria	Inclusion criteria:<br>Participants aged 30 through 67 years, under a physician's care for type 2 diabetes for at least 6 months before recruitment, had hemoglobin A1c (HbA1c) levels of 8.0% or higher at the time of entry, and had a serum C-peptide level higher than 1.0 ng/mL (to convert C-peptide to nanomoles per liter, multiply by 0.331) 90 minutes after a liquid mixed meal (250 calories, 6 g fat, 40 g carbohydrate, and 9 g protein). Participants had a BMI of 30.0 to 39.9 and were willing to accept randomization to either treatment group and follow the full treatment protocol. Additional criteria included the absence of conditions that would contraindicate surgery, such as serious cardiovascular disease, previous gastrointestinal surgery, psychological concerns, or history of malignancy.<br><br>Exclusion criteria:<br>N/R
Diabetes Remission Definition	Full remission was defined using the ADA consensus statement, required HbA1c 6.0% at 24 and 36 months, with no use of antihyperglycemic medication from 24 to 36 months.
Sample Size	N=120<br>I=60<br>C=60
Loss to Follow-Up	N=19(16%)<br>I=5(8%)<br>C=14(23%)
Age	Mean overall (SD)= 49(8)<br>Mean I (SD= 49(9)<br>Mean C (SD)= 49(8)
Gender	Female: I: 38(63); C: 34(57)<br>Male: N/R
Race/Ethnicity	Non-Hispanic white C:30 (50); I: 33 (55)<br>East Asian C:17 (28); I: 16 (27)<br>Non-Hispanic black C: 6 (10); I: 5 (8)<br>Hispanic C: 4 (7); I: 4 (7)<br>Native American C: 1 (2); I: 2 (3)<br>Other C: 2 (3); I:0 (0)
BMI	Mean overall (SD)= 34.6(3.1)<br>Mean I (SD)= 34.9(3.0)<br>Mean C (SD)= 34.3(3.1)
Comorbidities	N/R

Duration of Diabetes	Mean overall (years SD)= 9 (95% CI, 7.9-10.0 years)<br>Mean I (years SD)= 8.9(6.1)<br>Mean C (years SD)= 9.1(5.6)
Baseline A1C%	Mean overall (SD)= 9.6(1.1)<br>Mean I (SD)= 9.6(1.0)<br>Mean C (SD)= 9.6(1.2)
Description of Intervention	The 2-year lifestyle intervention was based on protocols from two successful clinical trials: the Diabetes Prevention Program (DPP) and the Look AHEAD (Action for Health in Diabetes) study. Over the first 12 months the median number of lifestyle modules delivered was 32 for lifestyle-medical management intervention and 27 for gastric bypass. Between 12 and 24 months the median number of modules was five and seven for lifestyle-medical management intervention and gastric bypass, respectively. Visits with an endocrinologist occurred monthly for 6 months, then quarterly (or monthly if not at ADA treatment goal) for the next 6 months, and then quarterly through the second year. The intensive medical management protocol for both treatment groups aimed to optimize drug therapy to control hyperglycemia, cholesterol, and hypertension.<br><br>After 24 months, all study interventions ceased and patients returned to usual care with their primary physician. Each subject's primary physician received a letter describing the study, the subject's current status, medications, and goals of care. The primary physicians also received recommendations about medications and information about the need for<br>nutritional supplementation as appropriate. The study coordinator contacted participants at 30 months to maintain their connection with the study and to obtain interim data on adverse events. Study endocrinologists evaluated patients during a clinic visit at 36 months but did not modify medications. The visit included a collection of blood pressure, weight, and waist circumference data and laboratory studies. Participants were encouraged to increase medication compliance, nutritional supplementation, and dietary control if adherence was deemed to be an issue.<br><br>Gastric bypass was laparoscopically performed in a standardized fashion with construction of a 20-mL lesser curvature gastric pouch and a 100-cm biliopancreatic limb. Study surgeons performed all post- operative surgical interventions.
Category of Intervention	RYGB
Who Delivered Intervention	Single surgeon, endocrinologist, and interventionist.
Location/Site of Delivery	Four teaching hospitals.
Description of Control	The 2-year lifestyle intervention was based on protocols from two successful clinical trials: the Diabetes Prevention Program (DPP) and the Look AHEAD (Action for Health in Diabetes) study. Over the first 12 months the median number of lifestyle modules delivered was 32 for lifestyle-medical management intervention and 27 for gastric bypass. Between 12 and 24 months the median number of modules was five and seven for lifestyle-medical management intervention and gastric bypass, respectively. Visits with an endocrinologist occurred monthly for 6 months, then quarterly (or monthly if not at ADA treatment

	goal) for the next 6 months, and then quarterly through the second year. The intensive medical management protocol for both treatment groups aimed to optimize drug therapy to control hyperglycemia, cholesterol, and hypertension.<br><br>After 24 months, all study interventions ceased and patients returned to usual care with their primary physician. Each subject's primary physician received a letter describing the study, the subject's current status, medications, and goals of care. The primary physicians also received recommendations about medications and information about the need for<br>nutritional supplementation as appropriate. The study coordinator contacted participants at 30 months to maintain their connection with the study and to obtain interim data on adverse events. Study endocrinologists evaluated patients during a clinic visit at 36 months but did not modify medications. The visit included a collection of blood pressure, weight, and waist circumference data and laboratory studies. Participants were encouraged to increase medication compliance, nutritional supplementation, and dietary control if adherence was deemed to be an issue.
Duration of Intervention and control	I: 2 years<br>C: 2 years
Length of Follow-Up Beyond Post-Intervention	5 years
List of Outcomes	Dichotomous outcomes:<br>1. HbA1c (<7%, <6%)<br>2. LCL cholesterol<br>3. SBP (<130mmHg, <140mmHg)<br>4. Remission (full or partial)<br>5. Use of prescription medication (insulin, other glycemic medicines, LDL medicine, and BP medications)<br>6. At goal without medication (triple end point, HbA1c (<7%), LDL cholesterol, and SBP (<130 mmHg)<br><br>Continuous outcomes:<br>1. HbA1C %<br>2. Serum lipids (LDL, HDL)<br>3. BP (SBP, DBP)<br>4. Weight<br>5. Percent weight change<br>6. BMI<br>7. Medications prescribed (for glycemia, dyslipidemia and BP)
Serious Adverse Events	At 3 years, 24 serious or clinically significant adverse events were observed in lifestyle-medical management vs. 51 with gastric bypass. 1 death from pancreatic cancer in control group.<br><br>1. Surgical complications<br>Anastomotic leak<br>Anastomotic ulcer<br>Anastomotic stricture

	Wound infection<br>Wound hematoma<br>Pouch gastritis<br>Small bowel obstruction<br>2. Gastrointestinal<br>Acute pancreatitis<br>Pancreatic carcinoma<br>Cholelithiasis<br>Abdominal pain<br>Reflux esophagitis<br>Duodenitis<br>3. Cardiovascular<br>Deep venous thrombosis<br>Congestive heart failure<br>Acute myocardial infarction<br>4. Renal<br>Nephrolithiasis<br>5. Metabolic<br>Diabetic ketoacidosis<br>6. Musculoskeletal<br>Below-the-knee amputation<br>Toe amputation<br>Foot amputation<br>7. Neurologic<br>Herniated spinal disc<br>Multiple sclerosis<br>Partial 3rd cranial nerve palsy<br>8. Psychiatric<br>Depression<br>Suicide attempt<br>9. Miscellaneous<br>Fall with fracture or injury<br>Hypertension w/hospitalization<br>Unwanted pregnancy<br>Abnormal uterine bleeding<br>Retinal vascular occlusion<br>Abdominoplasty Infection
Funding Source	National Institutes of Health National Institute of Diabetes and Digestive and Kidney Diseases Nutrition and Obesity Research Center grant no. P30-DK-050456.<br><br>National Center for Advancing Translational Sciences, National Institutes of Health, formerly the National Center for Research Resources, grants UL1 TR000040 and UL1 RR024156 to Columbia University.<br><br>Grant support from Covidien, EnteroMedics, USGI Medical, and ReShape Medical. J.K. Reports receiving institutional grant support from Covidien and receiving personal support for expert testimony and participation on the speakers bureau for Takeda. J.P.B. Reports receiving institutional support for the Look AHEAD study. A.J.T. and Q.W. Report receiving salary support from Covidien

	for the DSS, as well as supplemental salary support from the Minnesota Obesity Center. J.E.C. Reports receiving institutional and personal grant support from Covidien, National Institutes of Health grant support, and travel expenses and personal support for the U.S. Food and Drug Administration Advisory Panel on Pulmonary Drugs. M.D.J. Reports serving on an advisory board for Vivus and receiving institutional grant support from Aspire Bariatrics. A.V. Reports receiving consulting support from Sanofi, Roche, and Novartis; institutional consulting support from Merck; and institutional grant support from Covidien, Daiichi-Sankyo, Merck, and GI Dynamics. L.A. reports receiving institutional grant support from Covidien. C.J.B. Reports receiving grant support from Covidien and personal support for consultancy from EnteroMedics and Novo Nor- disk.
Comments	

Kehagias et al., 2023	
Country, Year	Greece, 2023
Question/Study Objective	The modification of metabolic LRYGBP with fundus resection, in patients with obesity and T2DM, may appear as a metabolic weapon in the armamentarium of bariatric surgeon by ensuring optimized diabetic control through overwhelming activation of neuroendocrine mechanisms.
Study Design	Prospective non-blinded RCT.
Inclusion/Exclusion Criteria	Inclusion criteria:<br>Twenty-four patients, aged 18 to 60 years old, with BMI ≥40kg/m2 and T2DM. The duration of T2DM was determined less than 8 years, since longer duration is associated with irreversible b-cell function impairment and less recovery postoperatively.<br><br>Exclusion criteria:<br>People with gestation, diabetes mellitus type I, alcohol or drug abuse, major depressive disorder, non-compliance with the medical personnel's instructions, and previous abdominal surgeries with altered GI anatomy.
Diabetes Remission Definition	Remission was defined as a fasting glucose <126mg/dl, unimpaired glucose values after 120-min oral glucose tolerance test 75gr (OGTT), HbA1c <6,5% and no use of anti- diabetic medications. Glycemic improvement and antidiabetic medications discontinued, without need for recommencing at 6 months.
Sample Size	N=24<br>I=12<br>C=12
Loss to Follow-Up	N=N/R<br>I=N/R<br>C=N/R
Age	Mean overall (SD)= 47(11)<br>Mean I (SD)= 49.9(9.8)<br>Mean C (SD)= 45.6(10.7)
Gender	Female: I: 6(50%); C: 7(58.3%)<br>Male: I: 6(50%); C: 5(41.7%)
Race/Ethnicity	N/R
BMI	Mean overall (SD)= 53(11)<br>Mean I (SD)= 49.7(7.8)<br>Mean C (SD)= 56.8(11.5)
Comorbidities	N/R
Duration of Diabetes	Mean overall (years SD)= N/R<br>Mean I (years SD)= 4.33(2.39)<br>Mean C (years SD)= 3.20(1.95)
Baseline A1C%	Mean overall (SD)= N/R<br>Mean I (SD)= 7.78(1.74)<br>Mean C (SD)= 8.11(1.66)
Description of Intervention	All the operations were completed laparoscopically by the same surgeon at the surgical department of the University Hospital of Patras.<br><br>Both procedures included the creation of a small gastric pouch of 30ml capacity, a very long biliopancreatic limb of 200cm and an alimentary limb of 150cm. The

	aforementioned lengths are common practice for LRYGBP in our bariatric and metabolic surgery unit. The gastrojejunal anastomosis was created with a circular stapler of 25mm diameter after transoral placement of the anvil with assistance from the anesthesiologist.<br><br>In the LRYGBP+FR group, the gastric body and fundus were mobilized by dividing the gastrocolic ligament and short gastric vessels until exposing the angle of His. After creating an L-shaped gastric pouch with the linear stapler, the fundus was resected with a horizontal transection at the level of the horizontal line of the gastric remnant. This selection was guided by previous experience with fundus resection modification in our department and relevant studies in the literature. A fundus with mean dimensions, ±5.5cm (width) and ±10cm (vertical length) was removed. The mean operative time was 18.1 ± 1,4 min longer in the LRYGBP+FR group.
Category of Intervention	RYGB<br>Gastric bypass
Who Delivered Intervention	Surgeon and anesthesiologist.
Location/Site of Delivery	Hospital.
Description of Control	All the operations were completed laparoscopically by the same surgeon at the surgical department of the University Hospital of Patras.<br><br>Both procedures included the creation of a small gastric pouch of 30ml capacity, a very long biliopancreatic limb of 200cm and an alimentary limb of 150cm. The aforementioned lengths are common practice for LRYGBP in our bariatric and metabolic surgery unit.<br><br>The gastrojejunal anastomosis was created with a circular stapler of 25mm diameter after transoral placement of the anvil with assistance from the anesthesiologist.
Duration of Intervention and control	I: 1 year<br>C: 1 year
Length of Follow-Up Beyond Post-Intervention	N/R
List of Outcomes	Primary outcome:<br>1. HbA1c levels at one-year follow-up.<br><br>Secondary outcomes:<br>1. BMI<br>2. Excess weight loss (EWL)<br>3. Glycemic parameters (glucose, C-peptide, insulin, insulinogenic index, HOMA-IR)<br>4. GI hormones (ghrelin, GLP-1, PYY).<br><br>* All were reported as means and SD, with p-values.

Serious Adverse Events	Neither intraoperative complications nor conversion to open were reported.
Funding Source	Open access funding provided by HEAL-Link Greece.
Comments	

Lee et al., 2011	
Country, Year	Taiwan, 2011
Question/Study Objective	To determine the efficacies of 2 weight reducing operations on diabetic control and the role of duodenum exclusion.
Study Design	Double blind RCT.
Inclusion/Exclusion Criteria	Inclusion criteria:<br>Participants aged >30 and <60 years old, had a BMI of >25 to <35, had been diagnosed as having clearly documented but poorly controlled (HbA1c >7.5% [to convert to proportion of total hemoglobin, multiply by 0.01]) T2DM and were treated by an endocrinologist for 6 months or longer, had no evidence of renal impairment or diabetic retinopathy, and were able to understand and comply with the study process.<br><br>Exclusion criteria:<br>Participants who had a specific disease; had previously undergone bariatric surgery; had a history of major medical problems, such as mental impairment, drug or alcohol addiction, a recent major vascular event, internal malignant neoplasm, or portal hypertension; or had a contradiction for either surgery. Participants were excluded if their C-peptide level was below 1.0 ng/mL (to convert to nanomoles per liter, multiply by 0.331) or they did not attend 2 initial information visits.
Diabetes Remission Definition	A fasting plasma glucose levels less than 126 mg/dL, in addition to HbA1c values less than 6.5% without the use of oral hypoglycemics or insulin.
Sample Size	N=60<br>I=30<br>C=30
Loss to Follow-Up	N=0(0%)<br>I=0(0%)<br>C=0(0%)
Age	Mean overall (SD)= 45 (range, 34-58)<br>Mean I (SD)= N/R<br>Mean C (SD)= N/R
Gender	Female: N/R<br>Male: N/R
Race/Ethnicity	N/R
BMI	Mean overall (SD)= 30.3 (range, 25-34)<br>Mean I (SD)= N/R<br>Mean C (SD)= N/R
Comorbidities	N/R
Duration of Diabetes	Mean overall (years SD)= N/R<br>Mean I (years SD)= N/R<br>Mean C (years SD)= N/R
Baseline A1C%	Mean overall (SD)= 10 (range 7.5-15)<br>Mean I (SD)= N/R<br>Mean C (SD)= N/R
Description of Intervention	Gastric bypass with duodenum exclusion:<br>For the case of GB surgical procedure, a simplified laparoscopic mini-GB was adopted. To describe briefly, a long-sleeved gastric tube was created (EndoGIA;

	Coviden), approximately 2.0 cm wide along the less curved side from the antrum to the angle of His. A loop gastroenterostomy (Billroth II anastomosis) was created with the small bowel approximately 120 cm distal to the ligament of Treitz. No drainage tube was left.
Category of Intervention	Gastric bypass<br>Sleeve gastrectomy
Who Delivered Intervention	Surgical team.
Location/Site of Delivery	Department of Surgery of the Min-Sheng General Hospital, National Taiwan University.
Description of Control	Sleeve gastrectomy without duodenum:<br>An SG was performed by resecting the greater curvature from the distal antrum (4 cm proximal to the pylorus) to the angle of His, including the complete fundus, by using a laparoscopic stapler (EndoGIA; Coviden, Norwalk, Connecticut) with 60-mm cartridges (3.5-mm stapler height, blue load). The remnant stomach tube was approximately 2 cm wide along the less curved side. The resected portion of the stomach was extracted from the extended periumbilical trocar site. A running absorbable suture was applied to the stapler line to prevent hemorrhage and leakage. No drainage tube was left.
Duration of Intervention and control	I: 1 year<br>C: 1 year
Length of Follow-Up Beyond Post-Intervention	N/R
List of Outcomes	Primary outcome:<br>Glycemic control at 12 months (diabetes remission).<br><br>Secondary outcome:<br>Percentage change in HbA1c levels, weight, blood pressure, waist circumference, and levels of fasting lipids, including total cholesterol, triglyceride, and high-density and low-density lipoprotein cholesterol. Changes in medication use, changes in the proportion of participants with metabolic syndrome as defined by the National Cholesterol Education Program Adult Treatment Panel III criteria,22 and changes in indirect measures of insulin resistance using the homeostasis model assessments were measured.
Serious Adverse Events	Late complications occurred in 2 patients (3%), 1 in each group, and required hospitalization for conservative treatment, but no major adverse effects were observed.
Funding Source	This work was supported by grant MS 97-2314-B-002-065 from the Min-Sheng General Hospital.
Comments	

Lin et al., 2022	
Country, Year	China, 2022
Question/Study Objective	In this study, we performed a single-center, randomized, controlled trial to compare loop DJB-SG with Roux-en-Y DJB-SG with different reconstruction methods but the same size of gastric sleeve and length of intestine bypassed in patients with T2D with preoperative body mass indexes (BMIs) of 27.5–40 kg/m2.
Study Design	RCT
Inclusion/Exclusion Criteria	Inclusion criteria:<br>Patients with T2D with BMIs of 27.5–40 kg/m2, hemoglobin A1C (HbA1C) .7.0% (53 mmol/mol), aged 20–60 years, T2D duration ,15 years, fasting C peptide .370 pmol/L, and meeting one of the following conditions: (1) BMI !32.5 kg/m2, (2) BMI of 27.5–32.5 kg/m2 with cardiovascular risk factors such as high triglycerides and low high- density lipoprotein cholesterol, hypertension, or diabetes complications.<br><br>Exclusion criteria:<br>(1) type 1 diabetes, latent autoimmune diabetes in adults, gestational diabetes, and other special types of diabetes;<br>(2) Los Angeles Classification grade C or D gastroesophageal reflux disease (GERD);<br>(3) drug or alcohol addiction or uncontrollable mental illness;<br>(4) intellectual disability or immaturity;<br>(5) those with unrealistic anticipation of the surgery;<br>(6) unwillingness to bear the risk of potential surgical complications;<br>(7) inability to cooperate with postoperative diet and life- style changes;<br>(8) poor general condition and unable to tolerate general anesthesia or surgery; and<br>(9) decision not to participate.
Diabetes Remission Definition	Remission was defined as a return of HbA1C to ,6.5% (48 mmol/mol) after surgery and persists for at least 3 months in the absence of usual glucose-lowering pharmacotherapy.
Sample Size	N=96<br>I=48<br>C=48
Loss to Follow-Up	N=7(7.3%)<br>I=4(8.3%)<br>C=3(6.25%)
Age	Mean overall (SD)= N/R<br>Mean I (SD)= 36.94(8.76)<br>Mean C (SD)= 36.85(7.42)
Gender	Female I: 32(66.7%); C: 32(66.7%)<br>Male: I: 16(33.3%), C: 16(33.3 %)
Race/Ethnicity	N/R
BMI	Mean overall (SD)=N/R<br>Mean I (SD)= 33.68(3.46)<br>Mean C (SD)= 33.43(3.47)
Comorbidities	Hypertension<br>Lipidemia
Duration of Diabetes	Mean overall (years SD)= N/R<br>Mean I (years SD)= 3.16(2.65

	Mean C (years SD)= 4.22(3.96)
Baseline A1C%	Mean overall (SD)= N/R<br>Mean I (SD)= 8.78(1.18)<br>Mean C (SD)= 8.93(1.26)
Description of Intervention	All operations were performed laparoscopically. Briefly, the greater omentum was mobilized first, and the right gastroepiploic vessels were transected. After that, a passageway behind the duodenal bulb was created at a level of 3 cm from the pylorus, and the duodenum was transected by the lineal stapler (60 mm). Then the stomach was transected from the antrum (4 cm proximal to the pylorus) to the fundus (1.5 cm distal to angle of His) over a 38-F bougie. The staple line was reinforced in hybrid manner [16].<br><br>For loop DJB-SG, an enterotomy was made at the 200 cm jejunum from the ligament of Treitz. Duodenotomy was done at the duodenal bulb, and a side-to-side duodenojejunal anastomosis (DJA) was performed with a continuous inverting suture. The anastomosis was reinforced by a running suture of the seromuscular layer.
Category of Intervention	RYGB<br>Sleeve gastrectomy<br>Duodenojejunal bypass with sleeve gastrectomy
Who Delivered Intervention	N/R
Location/Site of Delivery	Hospital
Description of Control	For Roux-en-Y DJB-SGs, 100 cm of the proximal jejunum was measured, and DJA was performed similar to a loop DJB-SG. After transecting the biliopancreatic limb (1 cm proximal to the DJA), another 100 cm of the jejunum (Roux limb) was measured, and a side-to-side jejunojejunal anastomosis was done with a 60-mm linear stapler. The anastomotic defect was closed by hand suture. The mesenteric and Petersen defects were closed. Meanwhile, 2 patients undergoing loop DJB-SG received synchronous hiatal hernia repair by simple cruroplasty. Drainage was placed as a routine procedure. All patients were requested to take proton-pump inhibitors for 4 weeks and supplement with the multivitamin and multi-mineral complex as well as protein powder for at least 1 year or longer if malnutrition exists.
Duration of Intervention and control	I: 12 months<br>C: 12 months
Length of Follow-Up Beyond Post-Intervention	N/A
List of Outcomes	Weight loss and metabolic outcomes:<br>1. Weight (kg)<br>2. BMI (kg/m2)<br>3. TWL (%) total weight loss<br>4. EWL (%) excessive weight loss<br>5. HbA1C (%)<br>6. FBG (mmol/L) fasting blood glucose<br>7. T2D remission: Cr(n) complete remission, PR(n) partial remission

	8. TC (mmol/L) total cholesterol<br>9. TG (mmol/L) total triglycerides<br>10. LDL (mmol/L)<br>11. HDL (mmol/L)<br>12. Uric acid (umol/L)<br>13. Hypertension (n)<br><br>Changes of nutrition status after surgery:<br>1. Hemoglobin (g/L)<br>2. Anemia (n)<br>3. Iron (umol/L)<br>4. Albumin (g/L)<br>5. Calcium (nmol/L)<br>6. Folic acid (nmol/L)<br>7. Vitamin D (nmol/L)<br>8. Vitamin B12 (pmol/L)<br><br>Surgical outcomes, major post-operative complications, and 1-year complaints:<br>1. Surgical outcomes (operative time (min); medical expenses; postoperative length of stay (d)<br>2. Major postoperative complications (leak (n); hemorrhage (n); reoperation (n)<br>3. Major 1-year complaints (GERD symptoms (n); malodorous flatus (n); hair loss (n); steatorrhea (n); hypoglycemias (n); orthostatic hypotension (n)
Serious Adverse Events	The overall incidence of early major perioperative complications was 6.3% (6 of 96), including 4 DJA leaks (2 in each group), 1 trocar site bleed, and 1 gastrointestinal bleed in the Roux-en-Y DJB-SG group. There were no strictures in any patient. Among 4 patients with DJA leaks, 2 underwent emergency laparotomy (1 in each group), and the other 2 received conservative treatments and recovered. Patients with postoperative bleeding were treated conservatively and recovered.<br><br>At 1-year follow-up, late complications or complaints were de novo GERD symptoms (25.0%; 22 of 88), malodorous flatus (22.7%; 20 of 88), hair loss (17.05%; 15 of 88), steatorrhea (5.7%; 5 of 88), hypoglycemia (4.5%; 4 of 88), and orthostatic hypotension (2.3%; 2 of 88). There was no statistical difference between the 2 groups.
Funding Source	Funded by the Bethune Charitable Foundation (HZB-20190528-1) and Approval Programs in 3 Issue Advantageous Disciplinary Construction Projects of Jiangsu colleges and universities.
Comments	*Lost to follow-up: For DJB-SB, in addition to 4 lost to follow-up, 1 was converted to RYGB but not added to lost to follow-up.

Mingrone et al., 2012	
Country, Year	Italy, 2012
Question/Study Objective	To compare the efficacy of two types of bariatric surgery (gastric bypass and biliopancreatic diversion) with conventional medical therapy in severely obese patients with type 2 diabetes.
Study Design	Single-center, nonblinded RCT.
Inclusion/Exclusion Criteria	Inclusion criteria:<br>Participants with an age of 30 to 60 years, a body-mass index (BMI, the weight in kilograms<br>divided by the square of the height in meters) of 35 or more, a history of type 2 diabetes of at least 5 years, a glycated hemoglobin level of 7.0% or more (as confirmed by at least three analyses), and an ability to understand and comply with the study protocol.<br><br>Exclusion criteria:<br>Participants with a history of type 1 diabetes, diabetes secondary to a specific disease or<br>glucocorticoid therapy, previous bariatric surgery, pregnancy, other medical conditions requiring short-term hospitalization, severe diabetes complications, other severe medical conditions, and geographic inaccessibility
Diabetes Remission Definition	Defined as a fasting glucose level of <100 mg per deciliter [5.6 mmol per liter] and a glycated hemoglobin level of <6.5% in the absence of pharmacologic therapy).
Sample Size	N=60<br>I=40<br>C=20
Loss to Follow-Up	N=4<br>I=2<br>C=2
Age	Mean overall (SD)= N/R<br>Mean I (SD)= BD: 42.75 (8.06); GB: 43.9 (7.57)<br>Mean C (SD)= 43.45 (7.27)
Gender	Female: I: 22 (55); C: 10 (50)<br>Male: I: 18 (45); C: 10 (50)
Race/Ethnicity	N/R
BMI	Mean overall (SD)= N/R<br>Mean I (SD)= BD: 45.14(7.78); GB: 44.85 (5.16)<br>Mean C (SD)= 45.62 (6.24)
Comorbidities	Hypertension<br>Elevated lipid levels
Duration of Diabetes	Mean overall (years SD)= N/R<br>Mean I (years SD)= BD: 6 (1.26); GB: 6.03 (1.18)<br>Mean C (years SD)= 6.08 (1.24)
Baseline A1C%	Mean overall (SD)= N/R<br>Mean I (SD)= BD: 8.88 (1.71); GB: 8.56 (1.4)<br>Mean C (SD)= 8.51 (1.24)
Description of Intervention	Daily multivitamin and mineral supplementation was prescribed to the surgical groups; patients undergoing biliopancreatic diversion received additional vitamin D and calcium supplementation.

	Roux-&-Y Gastric Bypass (RYGB) involves the use of a surgical stapler to create a small and vertically oriented gastric pouch with a volume usually < 30 ml. The upper pouch is completely divided by the gastric remnant and is anastomosed to the jejunum, 75 cm distally to the Treitz's ligament , through a narrow gastrojejunal anastomosis in a Rouxen-Y fashion. Bowel continuity is restored by an entero-entero anastomosis, between the excluded biliary limb and the alimentary limb, performed at 100 cm from the gastrojejunostomy. The operation was performed in laparoscopy.<br><br>Bilio-pancreatic diversion (BPD) consists of an about 60% distal gastric resection with stapled closure of the duodenal stump. The residual volume of the stomach is about 300 ml. The small bowel is transected at 2.5 m from the ileo-cecal valve, and its distal end is anastomosed to the remaining stomach. The proximal end of the ileum, comprising the<br>remaining small bowel carrying the bilio-pancreatic juice and excluded from food transit, is<br>anastomosed in an end-to-side fashion to the bowel 50 cm proximal to the ileo-caecal valve.<br>Consequently, the total length of absorbing bowel is brought to 250 cm, the final 50 cm of<br>which, the so-called common channel, represents the site where ingested food and biliopancreatic juices mix. BPD was performed through traditional open surgery.
Category of Intervention	Gastric bypass<br>Biliopancreatic diversion
Who Delivered Intervention	Two teams of bariatric surgeons, one with expertise in laparoscopic gastric bypass and the other with expertise in open biliopancreatic diversion, performed the procedures.
Location/Site of Delivery	The Day Hospital of Metabolic Diseases and Diabetology of the Catholic University in Rome.
Description of Control	Patients in the medical-therapy group were assessed and treated by a multidisciplinary team<br>that included a diabetologist, a dietitian, and a nurse, with planned visits at baseline and at 1, 3, 6, 9, 12, and 24 months after study entry. Oral hypoglycemic agents and insulin doses<br>were optimized on an individual basis with the aim of reaching a glycated hemoglobin level of less than 7%. Programs for diet and lifestyle modification, including reduced overall energy and fat intake (<30% total fat, <10% saturated fat, and high fiber content) and increased physical exercise (≥30 minutes of brisk walking every day, possibly associated with moderate-intensity aerobic activity twice a week), were designed by an experienced<br>diabetologist with assistance from a dietitian.
Duration of Intervention and control	I: 2 years<br>C: 2 years
Length of Follow-Up Beyond Post-Intervention	5- and 10-year follow-up.

List of Outcomes	Primary outcome:<br>Partial and complete diabetes remission.<br><br>Secondary outcomes:<br>Changes from baseline in levels of fasting plasma glucose and glycated hemoglobin, the average glycated hemoglobin level, body weight, waist circumference, arterial blood pressure, and levels of plasma cholesterol, HDL cholesterol, and triglycerides at<br>2 years.
Serious Adverse Events	There were no operative deaths among patients undergoing either gastric bypass or biliopancreatic diversion. An incisional hernia requiring reoperation 9 months after surgery developed in a patient undergoing biliopancreatic diversion, and one patient undergoing gastric bypass had an intestinal obstruction requiring reoperation 6 months after surgery. Two patients who were receiving medical therapy had persistent diarrhea associated with metformin, a condition that resolved when the drug was discontinued and another oral<br>hypoglycemic agent was substituted.<br><br>At 10-year follow-up: DVT, PE, Afib, Hernia, diarrhea, intestinal occlusion, anemia, hypoalbuminaemia, osteopenia, osteoporesis, nyctalopia, renal calculus, symptomatic hypoglycemia, MI, rentinopathy, neuropathy.
Funding Source	Funded by the Catholic University of Rome.
Comments	*Two bariatic surgeries were compared against a medical treatment group (1:1:1). Values are listed above BD= biliopancreatic diversion and GB= gastric bypass.

Moriconi et al., 2022	
Country, Year	Italy, 2022
Question/Study Objective	The aim of this study was to determine the impact of RYGB on T2DM over 10-year follow-up, focusing on the incidence of T2DM relapse after the initial remission in order to identify potential predictive factors.
Study Design	A single-center, open, 10-year prospective study.
Inclusion/Exclusion Criteria	Inclusion criteria:<br>Age between 40 and 65 years, body mass index (BMI) ≥ 35 kg/m2, T2DM diagnosis according to ADA criteria. Insulin-taking patients whose age at diabetes onset was ≥ 40 years and who were negative for the presence of islet autoantibodies were also considered to have T2DM.<br><br>Exclusion criteria:<br>Severe medical conditions (malignancies, liver cirrhosis, end-stage kidney disease, connective tissue or endocrine disease), history of type 1 diabetes mellitus or secondary forms of diabetes, previous bariatric surgery or pregnancy.
Diabetes Remission Definition	Diabetes remission (DR) was defined as the combination of partial and complete remission according to ADA criteria. Specifically, partial remission was defined as HbA1c < 6.5% and fasting glucose 100−125 mg/dL, while complete remission was defined as return to fasting glucose < 100 mg/dL and HbA1c <6.0% for at least one year in the absence of pharmacologic therapy or ongoing procedures.
Sample Size	N=127<br>I=96<br>C=31
Loss to Follow-Up	N=14(11)<br>I=8(8.3)<br>C=6(19.3)
Age	Mean overall (SD)= N/R<br>Mean I (SD)= 53(8)<br>Mean C (SD)= 54(7)
Gender	Female I: I: 67 (76.1); C: 12 (48)<br>Male: I: 21 (23.9); C: 13 (52)
Race/Ethnicity	N/R
BMI	Mean overall (SD)=N/R<br>Mean I (SD)= 46.7(6.8)<br>Mean C (SD)= 45.5(5.1)
Comorbidities	Hypotension
Duration of Diabetes	Mean overall (years SD)= N/R<br>Mean I (years SD)= 2.5 (1-10 range)<br>Mean C (years SD)= 2 (1-4.5 range)
Baseline A1C%	Mean overall (SD)= N/R<br>Mean I (SD)= 7.9 (1.6)<br>Mean C (SD)= 8 (1.1)
Description of Intervention	Height, weight, systolic and diastolic blood pressure were recorded pre-surgery, peripheral blood samples were obtained for determination of routine blood chemistry, electrolytes, complete blood count, lipid profile, plasma glucose and HbA1c concentrations; kidney and liver function tests were also recorded. Before

	surgery, patients were evaluated by a multidisciplinary team, including a surgeon, an anesthesiologist, a diabetologist, a dietician, a psychologist and a psychiatrist.<br><br>Patients in the surgery group admitted to our ward two days before surgery and returned three days after the intervention for another three days, in order to check glucose control and adjust drug therapy. On the day of surgery, patients with a fasting plasma glucose concentration >8.0 mmol/l were started on an insulin infusion adjusted to maintain fasting plasma glucose between 6.0 and 8.0 mmol/l during surgery and for the following day until they started eating again. Before surgery, 27 patients were on insulin therapy, 20 patients were on sulfonylureas plus metformin, and 58 patients took metformin alone. A small group of 8 patients had sulfonylureas alone due to intolerance to metformin or a combination of metformin and exenatide.<br><br>After identification of the Treitz ligament, the jejunum was transected 120 cm from the ligament of Treitz and an entero-enterostomy a 45-mm linear stapler was performed at 150 cm on the alimentary limb. A sub cardinal gastric pouch with a 20−30 ml capacity was divided from the gastric remnant by sectioning the stomach with multiple 45-mm linear stapler firings, and a 2.5−3 cm long gastrojejunal anastomosis was performed using a hand-sewn technique.
Category of Intervention	RYGB
Who Delivered Intervention	Before surgery, patients were evaluated by a multidisciplinary team, including a surgeon, an anesthesiologist, a diabetologist, a dietician, a psychologist and a psychiatrist.
Location/Site of Delivery	An outpatient clinic of the Internal Medicine Unit of the Azienda Ospedaliero-Universitaria Pisana (Pisa, Italy).
Description of Control	In the MT group patients were treated by oral anti-hyperglycemic agents and/or insulin based on individual characteristics with the aim of reaching HbA1c levels < 6.5%. Patients received nutritional counseling and a low-calorie diet tailored to achieve a weight loss > 10% of initial body weight.
Duration of Intervention and control	I: 10 years<br>C: 10 years
Length of Follow-Up Beyond Post-Intervention	N/A
List of Outcomes	1. BMI<br>2. Fasting glucose<br>3. HbA1c<br>4. Total cholesterol<br>5. HDL<br>6. LDL<br>7. Triglycerides<br>8. eGFR<br>9. Hypertension<br>10. SBP, DBP<br>11. Metformin alone<br>12. Insulin therapy

Serious Adverse Events	N/R
Funding Source	The study was supported by a grant from the European Foundation for the Study of Diabetes (EFSD, “Clinical Research Grant in Diabetes”, Dusseldorf, Germany).
Comments	

Murphy et al., 2018	
Country, Year	New Zealand, 2018
Question/Study Objective	Achieving T2D remission defined as using four different HbA1c thresholds in the absence of glucose lowering therapy.
Study Design	Double blind RCT
Inclusion/Exclusion Criteria	Inclusion criteria:<br>Participants aged 25-50 years, BMI of 35 to 65 kg/m2, T2D diagnosed for at least 6 months, and must be suitable for surgical procedure and committed to follow up.<br><br>Exclusion criteria:<br>Participants with postprandial C peptide < 350 mmol/L, pregnant, with type 1 diabetes or secondary diabetes, chronic pancreatitis, those on oral steroid therapy, current smokers and those not suitable for general anesthesia.
Diabetes Remission Definition	Defined using four different HbA1c thresholds in the absence of glucose lowering therapy including [≤ 38 mmol/mol (5.6%), < 42 mmol/ mol (6.0%), < 48 mmol/mol (6.5%), or < 53 mmol/mol (7.0%)].
Sample Size	N=114<br>I=56<br>C=58
Loss to Follow-Up	N= 5(4%)<br>I= 0(0%)<br>C= 5(9%)
Age	N= 47(7)<br>I= 46.6(6.7)<br>C= 45.5(6.4)
Gender	Female I: 33 (59%); C: 26 (45%)<br>Male I: 41%; C: 55%
Race/Ethnicity	NZ European I: 34 (61%)<br>NZ European C: 38 (66%)<br>Māori I: 11 (20%); Māori C: 9 (16%)<br>Pacific Island I: 6 (11%), Pacific Island C: 4 (7%)<br>Other I: 5 (9%); Other C: 7 (12%)
BMI	N= 43(6)<br>I= 42.2(6.2)<br>C= 41.9 (5.9)
Comorbidities	N/R
Duration of Diabetes	N/R
Baseline A1C%	N=7.9(1.4)%<br>I=64.5 ± 18.1 mmol/mol; FU: 8.1 (1.7)<br>C=61.9 ± 12.8 mmol/mol; FU: 7.7 (1.1)
Description of Intervention	Briefly, this was a single-center, prospective, randomized, parallel 2-arm, double-blind (patient and assessor) clinical trial conducted in

	Auckland, New Zealand, in- volving 114 obese patients with T2D who were randomized 1:1 to LSG or SR-LRYGB (both routine procedures at our institution). SR-LRYGB consisted of a vertical lesser curve- based 20–30 ml gastric pouch, a 100-cm antecolic ante gastric Roux limb, a 50-cm biliopancreatic limb, with a 6.5- or 7-cm silastic ring placed around the gastric pouch, approximately 2 cm above the gastrojejunal anastomosis. Mesenteric defects were closed routinely. Methylene blue dye leak test was performed irrespective of procedure and port placements were identical for both procedures.
Category of Intervention	RYGB<br>Sleeve gastrectomy
Who Delivered Intervention	N/R
Location/Site of Delivery	Hospital
Description of Control	LSG consisted of gastric resection using an Echelon flex 45 stapler (Ethicon) alongside a 36Fr orogastric bougie, commencing 2 cm from the pylorus. Methylene blue dye leak test was performed irrespective of procedure and port placements were identical for both procedures.
Duration of Intervention and control	I= 1 year<br>C=1 year
Length of Follow-Up Beyond Post-Intervention	7 years
List of Outcomes	Secondary outcomes (at 1 year):<br>Weight loss, blood pressure, lipid levels, metabolic medication use, quality of life, anxiety and depressive symptoms (assessed using the RAND 36-item health survey (SF-36) and the Hospital Anxiety and Depression Scale (HADS)
Serious Adverse Events	There were no deaths in either group. Five patients in the SRLRYGB group required additional surgical intervention within the first 1 year: a laparoscopic washout, omental patch and feeding gastrostomy for jejunal perforation after endoscopic dilatation of a stricture following an earlier subclinical anastomotic leak (n = 1), laparotomy for mesenteric bleeding (n = 1), removal of silastic ring for vomiting (n = 1), laparoscopic omental patch and washout for perforated stomal ulcer (n = 1) and one drainage of wound abscess (n = 1). Three patients in the LSG group required additional surgical intervention: a laparoscopic cholecystectomy (n =1), revision to SR-LRYGB for sleeve stricture (n = 1) and wound debridement for infection (n =1).
Funding Source	Waitemata District Health Board; Johnson and Johnson (NZ), Covidien (NZ), and Obex (NZ)
Comments	laparoscopic sleeve gastrectomy (LSG) = control versus laparoscopic Roux-en-Y gastric bypass (LRYGB) = intervention

Parikh et al., 2014	
Country, Year	USA, 2014
Question/Study Objective	The purpose of this study was two-fold:<br>1) to conduct a pilot randomized trial to compare bariatric surgery to MWM in patients with T2DM and BMI 30–35 who otherwise met NIH criteria for surgery, and<br>2) to assess the role of sRAGE as a biomarker for predictor of success after surgery.
Study Design	RCT.
Inclusion/Exclusion Criteria	Inclusion criteria:<br>Participants with T2DM and BMI 30–35 who were otherwise eligible for bariatric surgery by NIH criteria, specifically:<br>(1) overweight for at least 5 years<br>(2) failure to lose weight with non-surgical means<br>(3) absence of medical or psychological contraindications<br>(4) patient understanding of the procedure and its risks<br>(5) strong motivation to comply with the post-surgical regimen<br><br>Exclusion criteria:<br>Participants unable to comply with the study protocol (either self-selected or by indicating during screening that s/he could not complete all requested tasks), participation in other obesity- or diabetes-related clinical trials, or diagnosis of cognitive dysfunction or significant psychiatric comorbidity.
Diabetes Remission Definition	Diabetes remission was defined as no longer meeting the American Diabetes Association criteria for T2DM, without the use of diabetes medications. T2DM was defined based on the American Diabetes Association (ADA) criteria:<br>(1) fasting glucose ≥ 126 mg/dL or<br>(2) glucose ≥ 200 at 120 minutes after 75 g oral glucose load or (3) HbA1c ≥ 6.5%.19
Sample Size	N=57<br>I=29<br>C=28
Loss to Follow-Up	N=13(23%)<br>I=9(31%)<br>C=4(14%)
Age	Mean overall (SD)= N/R<br>Mean I (SD)= 46.8 (8.1)<br>Mean C (SD)= 53.9 (8.4)
Gender	Female: I: 23(79%); C: 22(79%)<br>Male: N/R
Race/Ethnicity	The majority of the participants were female Hispanic or non-Hispanic African-American.
BMI	Mean overall (SD)= N/R<br>Mean I (SD)= 32.8 (1.7)<br>Mean C (SD)= 32.4 (1.8)
Comorbidities	N/R
Duration of Diabetes	Mean overall (years SD)= N/R<br>Mean I (years SD)= N/R<br>Mean C (years SD)= N/R

Baseline A1C%	Mean overall (SD)= 7.8 (N/R)<br>Mean I (SD)= 7.7 (1.4)<br>Mean C (SD)= 7.9 (1.3)
Description of Intervention	Bariatric Surgery Protocol:<br>Patients randomized to surgery underwent bypass, band or sleeve gastrectomy based on<br>patient preference (drawing upon information learned in the monthly bariatric surgery<br>information seminar and during the surgeon consultation). All patients underwent thorough<br>evaluation by a surgeon, internist, nutritionist, and psychologist, and then completed a liquid<br>protein diet for two weeks prior to surgery to decrease hepatomegaly.<br><br>Laparoscopic gastric bypass was performed with a 150 cm Roux limb and 100 cm biliopancreatic limb. Laparoscopic adjustable gastric banding was performed utilizing the<br>pars flaccida technique and the Lap-Band AP-Standard (California, Inamed). Sleeve<br>gastrectomy was created over a 40Fr bougie, approximately 5–7 cm proximal to the pylorus,<br>utilizing bioabsorbable buttressing material on the staple line.<br><br>Postoperative dietary guidelines were based on the ASMBS Allied Health Nutritional Guidelines for the Surgical Weight Loss Patient. Patients were maintained on clear liquids for 48 hours postoperatively, then advanced to full liquid diet (including low-fat, low sugar, protein-rich shakes) for 2 weeks, followed by pureed diet for 2 weeks and then transitioned to regular diet. Patients were seen postoperatively at 2 weeks, 4 weeks and then monthly for the duration of the study. Band adjustments were done percutaneously in the clinic according to a commonly used algorithm based on hunger and weight loss.
Category of Intervention	RYGB<br>Sleeve gastrectomy<br>Gastric bypass<br>Gastric band
Who Delivered Intervention	Surgeon, internist, nutritionist, psychologist, and a bilingual weight loss clinician.
Location/Site of Delivery	Municipal health care system, the New York City Health and Hospitals Corporation (HHC).
Description of Control	Intensive Medical Weight Management (MWM) Protocol:<br>The MWM protocol was based on successful models of lifestyle counseling previously<br>published in the medical literature. These trials utilized intensive lifestyle interventions,<br>including frequent group and individual sessions focusing on nutrition and physical activity<br>counseling. For our short-term study, the weight loss goal was 5% of initial body weight at 6<br>months.

	MWM sessions were led by a bilingual weight loss clinician with an expertise in diabetes<br>education. Sessions were held weekly for the first month and then biweekly. In these 30-<br>minute sessions, the clinician offered culturally tailored, patient-specific counseling on diet,<br>physical activity, self-monitoring, and goal setting. The visits included a review of home<br>glucose data and adjustment of diabetes medications. In addition, participants were provided with pedometers to track their progress, with a goal of 150 min/week of low-impact physical activity by 6 months.<br><br>Patients randomized to the MWM arm were given the option to cross over to the surgical<br>arm after completing 6 months of MWM. The cross-over group existed to ensure compliance with the MWM group, as a previous randomized study conducted at our<br>institution (looking at a different research question involving MWM) suffered from high<br>drop-out/non-compliance in the MWM arm. A minimum of 2% weight loss was required<br>for all MWM participants in order to proceed with the surgery (to ensure maximum<br>compliance with the MWM arm).
Duration of Intervention and control	I: 6 months<br>C: 6 months
Length of Follow-Up Beyond Post-Intervention	N/R
List of Outcomes	Primary outcomes (assessed at 6 months):<br>HOMA-IR, Diabetes Remission, HbA1c, fasting glucose, glucose after OGTT, and requiring T2DM meds.<br><br>Secondary outcomes:<br>Changes in weight, BMI, %EWL, waist circumference, triglycerides, HDL, LDL, CHOL, systolic BP, and diastolic BP were measured as pre, post (6 months) and change.
Serious Adverse Events	There were no complications in the MWM arm. Hospitalizations unrelated to MWM were<br>not tracked in this study. There were no mortalities or life-threatening complications in the<br>surgery arm. There was one readmission with 30 days for dehydration (resolved with<br>intravenous fluids and anti-emetics) and there was one late readmission (>30 days) for a<br>trocar site abscess (resolved with percutaneous drainage and antibiotics).

Funding Source	This project was supported by grant number K12HS019473 from the Agency for Healthcare Research and Quality. This project was also supported by the NYU-HHC Clinical and Translational Science Institute which is supported in part by grant 1UL1RR029893 from the National Center for Research Resources, National Institutes of Health. Due to the fact that insurance companies typically cover bariatric surgery in patients with T2DM and BMI > 35, we partnered with our municipal healthcare system’s primary insurer, who agreed to cover the costs of the surgery as part of this research project.
Comments	

Singh et al., 2023	
Country, Year	N/R, 2023
Question/Study Objective	This randomized study was designed to compare OAGB and RYGB with remission of T2DM as the primary outcome.
Study Design	Open-labelled RCT.
Inclusion/Exclusion Criteria	Inclusion criteria:<br>All patients with a body mass index (BMI) ≥ 30 kg/m2 with concomitant T2DM were included from October 2017 until December 2021.<br><br>Exclusion criteria:<br>N/R
Diabetes Remission Definition	"Normal measures of glucose metabolism (HbA1c <6 %, FBG <100 mg/dL) in the absence antidiabetic medications."<br>- From article cited: Brethauer SA, Kim J, el Chaar M, et al. Standardized outcomes reporting in metabolic and bariatric surgery. Surg Obes Relat Dis. 2015;11(3):489–506.
Sample Size	N=49<br>I=25<br>C=24
Loss to Follow-Up	N=10(20.4%)<br>I=7(28%)<br>C=3(12.5%)
Age	Mean overall (SD)= N/R<br>Mean I (SD)= 45.8(9.1)<br>Mean C (SD)= 46.6(8.2)
Gender	Female: I: 18(72%); C: 16(66.67%)<br>Male: I: 7(28%); C: 8(33.33%)
Race/Ethnicity	N/R
BMI	Mean overall (SD)= N/R<br>Mean I (SD)= 47.0(6.7)<br>Mean C (SD)= 44.7(4.9)
Comorbidities	1. Hypertension<br>2. Obstructive sleep apnea<br>3. Nutritional indices (hemoglobin; percentage of patients with anemia and iron deficiency; albumin; vitamin D; vitamin B12; and folic acid)
Duration of Diabetes	Mean overall (years SD)= N/R<br>Mean I (years SD)= 3.4(2.2)<br>Mean C (years SD)= 4.6(6.0)
Baseline A1C%	Mean overall (SD)= N/R<br>Mean I (SD)= 7.9(1.0)<br>Mean C (SD)= 8.3(1.5)
Description of Intervention	All the procedures were performed laparoscopically.<br><br>For OAGB, a long gastric tube based on lesser curvature was fashioned over a 38 Fr bougie with a biliopancreatic limb length of 200 cm. RYGB was performed using a standard omega loop technique with a 70-cm biliopancreatic limb and 130-cm alimentary limb. Mesenteric defect and Peterson defect were closed in all patients in the RYGB group. Patients with intraoperative detection of cirrhosis or

	advanced liver fibrosis or other technical difficulty precluding a bypass procedure underwent Sleeve gastrectomy.
Category of Intervention	RYGB<br>Gastric bypass
Who Delivered Intervention	Surgeon.
Location/Site of Delivery	Hospital.
Description of Control	All the procedures were performed laparoscopically.<br><br>RYGB was performed using a standard omega loop technique with a 70-cm biliopancreatic limb and 130-cm alimentary limb. Mesenteric defect and Peterson defect were closed in all patients in the RYGB group. Patients with intraoperative detection of cirrhosis or advanced liver fibrosis or other technical difficulty precluding a bypass procedure underwent Sleeve gastrectomy.
Duration of Intervention and control	I: 4 years<br>C: 4 years
Length of Follow-Up Beyond Post-Intervention	N/R
List of Outcomes	The primary objective was to compare the complete remission and improvement rates of type 2 diabetes mellitus following OAGB and RYGB at 6 months, 12 months, and yearly thereafter.<br><br>1.Total weight loss (%TWL)<br>2. Percentage excess weight loss (%EWL)<br>3. Significant weight regain (WR)<br><br>Serum total protein, albumin, vitamin D, vitamin B 12, intact PTH, folate, and iron levels were measured at 3, 6, and 12 months postoperatively and then yearly.
Serious Adverse Events	Major Complications:<br>None of the patients in either group had a leak or bleeding. There was no 30-day mortality. One patient (4%) in the OAGB group (severe OSA with prolonged weaning from mechanical ventilation) and two patients (8.33%) in the RYGB group (post-operative pneumonia) had a prolonged stay beyond 7 days.<br><br>Minor Complications:<br>The early post-operative minor complication rate was 12% in the OAGB group (3 out of 25 patients) as compared to 12.5% in the RYGB group (3 out of 24 patients) ($p = 0.67$). Minor complications include: marginal ulcer, nausea and vomiting requiring IV fluids, tracer site SSI requiring local wound care, UTI managed with antibiotics, and iron/vitamin and mineral deficiency requiring IV supplementation.
Funding Source	N/R
Comments	* Country: All authors from India.

Techagumpuch et al., 2019	
Country, Year	Thailand, 2019
Question/Study Objective	The present study was conducted to compare the effectiveness of diabetic control at two years after gastric bypass surgery and sleeve gastrectomy among Thai patients.
Study Design	Single-blind RCT.
Inclusion/Exclusion Criteria	Inclusion criteria:<br>Male and female adults aged between 15 to 60 years who had:<br>1) body mass index (BMI) between 32.5 and 60<br>2) history of T2DM of less than 10 years<br>3) history of uncontrolled DM by medication treatment for more than six months and had HbA1c of more than 7 from preoperative evaluation at one week before surgery<br>4) ability to follow-up for glycemic control at the authors' hospital, and<br>5) performance status was safe for surgery, were included in the study after given an informed consent and agreed to participate in the study.<br><br>Exclusion criteria:<br>1) previous bariatric surgery<br>2) use of additional or alternative medication for weight control and DM control including steroid, diuretic (HCTZ), and Eltoxin.
Diabetes Remission Definition	Defined by having FPG less than 100, HbA1c less than 6 without any medication used within one year.
Sample Size	N=104<br>I=48<br>C=56
Loss to Follow-Up	N=0(0%)<br>I=0(0%)<br>C=0(0%)
Age	Mean overall (SD)= 38.12(N/R)<br>Mean I (SD= 36.93(10.76)<br>Mean C (SD)= 39.3(8.78)
Gender	Female: I: 21(43.75%); C: 24(42.86%)<br>Male: I: 27(56.25%); C: 32(57.14%)
Race/Ethnicity	"Thai patients."
BMI	Mean overall (SD)= 46.7 (N/R)<br>Mean I (SD)= 47.16(5.35)<br>Mean C (SD)= 46.12(6.6)
Comorbidities	N/R
Duration of Diabetes	Mean overall (years SD)= N/R<br>Mean I (years SD)= N/R<br>Mean C (years SD)= N/R
Baseline A1C%	Mean overall (SD)= 8.96%(N/R)<br>Mean I (SD)= 9.01(1.37)<br>Mean C (SD)= 8.93(1.52)
Description of Intervention	All operations were performed laparoscopically by the same surgeon and by the same surgical team. The patients were operated under general anesthesia in a

	reverse Trendelenburg position. A standard laparoscopic technique with four to five ports were used for both procedures.<br><br>For the sleeve gastrectomy, the procedure was started from mobilized greater curvature of stomach until reach angle of His. The stomach was then incised using linear staple with 4 cm antral sparing and the staple line was continued to create the residual stomach size equally to Bougie 36 Fr.<br><br>After the operation, all patients were educated about the nutrition, medication, and routine post- operative care. Patients with fasting plasma glucose (FPG) of more than 120 or HbA1c of more than 6% continued antiglycemic control medication according to ADA guideline for DM control(13). Follow-up was arranged at 2-week, 1-month, and every three months. At six-month and two-year after operation, the evaluation of FPG, HbA1c, and weight loss were performed by physician.
Category of Intervention	RYGB<br>Gastric bypass<br>Sleeve gastrectomy
Who Delivered Intervention	Surgeon, surgical team, and physician.
Location/Site of Delivery	The study was conducted in the Department of Surgery of the King Chulalongkorn Memorial University Hospital.
Description of Control	All operations were performed laparoscopically by the same surgeon and by the same surgical team. The patients were operated under general anesthesia in a reverse Trendelenburg position. A standard laparoscopic technique with four to five ports were used for both procedures.<br><br>In the bypass procedure, the pouch was created by using linear staple to archive the residual volume of 30 ml. The staple line started from the second branch of right gastric artery and vertically to angle of His. After that, the jejunojejunostomy was done using linear staple to create a 2 cm anastomosis length followed by creating an anticolic antegastric alimentary limb length 150 cm and biliary limb length 15 cm. Finally, the gastrojejunostomy was done using circular staple No.25.<br><br>After the operation, all patients were educated about the nutrition, medication, and routine post- operative care. Patients with fasting plasma glucose (FPG) of more than 120 or HbA1c of more than 6% continued antiglycemic control medication according to ADA guideline for DM control(13). Follow-up was arranged at 2-week, 1-month, and every three months. At six-month and two-year after operation, the evaluation of FPG, HbA1c, and weight loss were performed by physician.
Duration of Intervention and control	I: 2 years<br>C: 2 years
Length of Follow-Up Beyond Post-Intervention	N/R
List of Outcomes	1. Excess weight loss (%) at 6 months<br>2. Excess weight loss (%) at 2 years

	3. HbA1c level at 6 months after surgery<br>4. HbA1c level at 2 years after surgery<br>5. HbA1c level change (preop to postop) at 6 months HbA1c level change (preop to postop) at two years<br>6. Diabetic remission rate at 6 months (%)<br>7. Diabetic remission rate at 2 years (%)<br>8. FPG
Serious Adverse Events	The safety analysis, all procedures were successfully performed by a laparoscopic technique, with no deaths or major complications in both groups. Minor complication was found in two cases in LRYGB group with wound infection treated by local wound care. The mean postoperative hospital stay was 2.3 day in LSG and 3.2 days in LRYGB. All patients received follow-up at two years.
Funding Source	N/R
Comments	

Wallenius et al., 2020	
Country, Year	Sweden, 2020
Question/Study Objective	The aim of the present study was to compare diabetes remission rates in obese T2D patients (body mass index [BMI] 35–50) undergoing RYGB or SG. We hypothesized in line with the results of earlier studies that RYGB would be superior to SG in diabetes remission rates.
Study Design	RCT.
Inclusion/Exclusion Criteria	Inclusion criteria:<br>Participants with T2D requiring antidiabetic medications, BMI between 35 and 50 kg/m2, and age between 18 and 60 years.<br><br>Exclusion criteria:<br>Participants with an uncontrolled psychiatric disorder, alcohol and/or substance abuse, severe nephropathy (chronic kidney disease index .2), retinopathy or neuropathy,<br>had previously undergone surgical weight reducing procedures, or if Barrett's esophagus was diagnosed on preoperative endoscopy.
Diabetes Remission Definition	The primary endpoint was remission of T2D at 1 year after surgery with a planned follow-up time of 5 years. Diabetes remission was defined as HbA1C <6.0%, without diabetes medications.
Sample Size	N=60<br>I=29<br>C=31
Loss to Follow-Up	N=11<br>I=4<br>C=7+2
Age	Mean overall (SD)= N/R<br>Mean I (SD= 49.1 (9.2)<br>Mean C (SD)= 47.0 (10.7)
Gender	Female: I: 12(48); C: 11(45.8)<br>Male: I: 13(52); C: 13(54.2)
Race/Ethnicity	N/R
BMI	Mean overall (SD)= N/R<br>Mean I (SD)= 39.5 (3.7)<br>Mean C (SD)= 40.8 (4.1)
Comorbidities	Hypertension n(%) I: 20(80), C: 16(67)<br>Dyslipidemia n(%) I: 22(88), C: 23(96)
Duration of Diabetes	Mean overall (years SD)= N/R<br>Mean I (years SD)= 5.5 (4.1)<br>Mean C (years SD)= 5.0 (3.7)
Baseline A1C%	Mean overall (SD)= NR<br>Mean I (SD)= 7.9 (1.5)<br>Mean C (SD)= 8.2 (1.9)
Description of Intervention	All operations were performed by laparoscopy.<br><br>For RYGB, a 15- to 30-mL proximal gastric pouch was constructed using a 45-mm linear stapler. The biliopancreatic limb of approximately 50 cm was measured by hand-overhand

	along the mesenteric border. An antecolic, antegastric, end-to-side gastrojejunostomy was formed with a linear stapler combined with hand-suturing of the remaining opening. A 120-cm alimentary limb was constructed as described previously. The gastroenteroanastomosis and gastric pouch staple lines were tested intraoperatively for leakage by placing a gastrotube in the pouch and infusing methylene blue solution. Mesenteric defects were closed by clips or by suturing in all patients.<br><br>A standardized supplementation regimen of micronutrients was prescribed to all patients including daily intake of 100 to 200 mg iron, 1 g calcium, 800 to 1600 U of vitamin D3, 1 mg vitamin B12, and a multivitamin supplementation.
Category of Intervention	RYGB<br>Gastric bypass
Who Delivered Intervention	Surgeon.
Location/Site of Delivery	Bariatric centres in hospitals.
Description of Control	For SG, the gastrocolic ligament along the greater curvature was divided close to the stomach starting approximately 4 cm proximal from the pylorus along the greater curvature<br>of the stomach and up to the angle of His. Particular attention was paid to mobilizing the entire fundus to the midportion of the left crus of the diaphragm. A 35- or 36-Fr gastric calibration tube was placed along the lesser curvature. The tip of the first stapler cartridge was positioned so that a distance of one and a half times the width of a bougie at the<br>area of the incisura angularis was achieved. The resection was thereafter parallel to the lesser curvature and in contact with the calibration tube. The staple line was finished approximately 1 cm lateral to the angle of His. The stapler line was reinforced by a running suture and checked for leakage by infusion of methylene blue solution through the gastrotube. In the SG group 20 mg omeprazole was given daily for 2 months starting immediately after the operation.
Duration of Intervention and control	I: 1 year<br>C: 1 year
Length of Follow-Up Beyond Post-Intervention	2 years
List of Outcomes	Preoperatively, demographic data and the patients' medical history was collected. On all visits, including preoperatively, BMI (weight, height), waist circumference, blood pressure, and any ongoing medications for treatment of diabetes, dyslipidemia, or hypertension were recorded. Blood samples were collected for analysis of complete blood count, C-reactive protein, glycemic control (glycosylated hemoglobin [HbA1C], fasting glucose, insulin), lipid profiles, liver function tests, electrolytes and creatinine, iron, calcium, and vitamin D. Homeostasis model assessment of insulin resistance an index of insulin resistance, was calculated using the following formula: homeostasis model assessment of insulin<br>resistance 5 (insulin [mIU/ mL] ! fasting glucose [mmol/L]) / 22.5.

	Secondary outcomes at 30 days:<br>Surgical complication rates, weight loss, measured as reduction in BMI units, total weight loss (in kilograms), and percentage excess weight loss, as well as percentage weight loss, resolution of other co-morbidities associated with obesity and T2D.
Serious Adverse Events	One patient (4.2%) in the SG group and 3 patients (12%) in the RYGB group had intraoperative complications. In the SG group, 1 patient had a bleeding from the spleen that was controlled laparoscopically. Another patient (which was not considered a surgical complication) had to be converted to RYGB because of a thick and inflamed gastric antrum, where both the staple and hand-sewn sutures lacerated the tissue, and therefore this patient could not be operated by protocol but had to be converted to a RYGB, with resection of the remnant stomach. In the RYGB group, 2 patients had small bowel perforations, that were oversewn perioperatively, and 1 patient required a redo of the jejuno-jejunostomy because of obstruction. All these patients had uneventful recoveries postoperatively and normal length of stay at the hospital. Postoperative complications occurred in 2 patients after SG (8.3%). One patient was reoperated early because of a staple-line leak at the proximal part of the sleeve. The leaking part of the staple-line was restapled and the patient recovered uneventfully and was dismissed after 5 days from admission. The other patient had a suspected intra-abdominal abscess and was successfully treated conservatively with antibiotics and was dismissed 4 days after admission. There were no postoperative complications in the RYGB group. There were no deaths, and the average hospital stay was 2.5 6 .8 and 3.0 6 1.4 days in RYGB and SG groups, respectively.
Funding Source	VW reports grants from the Region Vastra G€otaland in Sweden (grant no. ALFGBG 813871) and grants from Erik and Lily Philipson memorial foundation. AT reports grants from the Erling-Persson Family Foundation (grant no. 140604). AM reports grants from the Health & Medical Care Committee of the Regional Executive Board, Region V€astra G€otaland in Sweden (grant no. VGFOUREG-384231).
Comments	* Loss to follow-up included: exclusion criteria, dropouts, non-compliance with pre-op, etc<br>Loss to follow-up at 24months (n=2) in control; n=2 withdrew consent, unclear if same 2 people as n=24 analyzed in control at 12 months, and n=22 analyzed at 24months (assuming same 2 people)<br><br>* Waist circumference, cm I: 127.9 (10.0); C: 129.6 (12.3)<br><br>* A1C also reported as mmol/mol mean(SD): I: 62.8 (16.1); C: 66.0 (21.0)

**Wentworth et al., 2014**	
Country, Year	Australia, 2014
Question/Study Objective	This trial was to establish whether laparoscopic adjustable gastric banding had a similar effect on glucose control in people with type 2 diabetes who were overweight but not obese (BMI 25–30 kg/m2). We postulated that laparoscopic adjustable gastric banding in addition to multidisciplinary diabetes care would improve glycaemic control with acceptable comparative costs and safety compared with multidisciplinary care alone.
Study Design	Single centre, open-label, parallel- group RCT.
Inclusion/Exclusion Criteria	Inclusion criteria:<br>Participants age between 18 and 65 years, BMI between 25 and 30 kg/m2, diabetes duration less than 5 years, willingness to be randomised to either study group, and ability to comply with the treatment protocol.<br><br>Exclusion criteria:<br>Positive glutamic acid decarboxylase autoantibody titre, pancreatic disease, previous bariatric surgery, or contraindication to laparoscopic adjustable gastric banding (including previous upper gastrointestinal surgery, hypothalamic disease, pregnancy, history of psychosis, or myocardial infarct in the preceding 6 months). If HbA1c was less than 6·5% (48 mmol/mol), diabetes was confirmed by oral glucose tolerance test.
Diabetes Remission Definition	Remission was defined as glucose concentrations of less than 7·0 mmol/L and less than 11·1 mmol/L 2 h after oral glucose, at least 2 days after stopping glucose-lowering drugs. Participants could be classified as in remission even if they had been taking glucose-lowering drugs continuously until 2 days before this test.
Sample Size	N=51<br>I=25<br>C=26
Loss to Follow-Up	N=3(6(%)<br>I=2(8%)<br>C=1(4%)
Age	Mean overall (SD)= N/R<br>Mean I (SD= 53(6)<br>Mean C (SD)= 53(7)
Gender	Female: I: 19(76%); C: 17(65%)<br>Male: I: 6(24%); C: 9(35%)
Race/Ethnicity	N/R
BMI	Mean overall (SD)= N/R<br>Mean I (SD)= 29(1)<br>Mean C (SD)= 29(1)
Comorbidities	N/R
Duration of Diabetes	Mean overall (years SD)= N/R<br>Mean I (years SD)= 2.2(1.7)<br>Mean C (years SD)= 2.8(1.8)
Baseline A1C%	Mean overall (SD)= N/R<br>Mean I (SD)= 6.9%(1.2)<br>Mean C (SD)= 7.2%(1.1)

Description of Intervention	Multidisciplinary diabetes care AND gastric banding surgery:<br>Patients in the gastric banding group underwent surgery within 3 months of randomisation. The LAP- BAND APS (Allergan Inc, Irvine, CA) was placed via the pars flaccida pathway and sited over the gastric cardia within 1 cm of the oesophago-gastric junction by one of three surgeons (PEO, WAB, PB). The basal fluid within the system at completion of the procedure was usually 4 mL. Incidental hiatal hernia was repaired as part of the procedure. Patient discharge was planned for roughly 3 h after completion of the procedure, and happened for all but one person. Aftercare to adjust the band and optimise eating and exercise behaviours was provided at The Centre for Bariatric Surgery (Melbourne, Australia) according to the management algorithms established by the centre.
Category of Intervention	Gastric band
Who Delivered Intervention	Surgeons and multidisciplinary care team (endocrinologists, clinical biochemistry, physician, dietitian, diabetes educator). Additional consultations with members of the care team were arranged if deemed necessary.
Location/Site of Delivery	Hospital.
Description of Control	Multidisciplinary diabetes care was based on guidelines from the American Diabetes Association16 and delivered in Melbourne by an endocrinologist. Biochemistry and physician review was done every 3 months in year 1, and every 6 months in year 2. Consultations with a dietitian and a diabetes educator were arranged within 3 months from enrolment in patients in the multidisciplinary care group or within 6 months in those in the laparoscopic adjustable gastric banding group. Additional consultations with members of the care team were arranged if deemed necessary by either JMW or the participant. If HbA1c was greater than 7% in year 2 of the trial, endocrinology review was scheduled every 3 months.<br><br>Participants were advised to do at least 150 min of moderate-intensity physical activity each week. HbA1c was targeted to less than 7·0% (54 mmol/mol) with use of drugs available through the Australian Pharmaceutical Benefit Scheme. Metformin was recommended to all participants unless it was not tolerated or if results of an oral glucose tolerance test were normal at 1 year. Sitagliptin or exenatide were commenced if HbA was 1c greater than 7·0% despite metformin or sulfonylurea therapy. Insulin was commenced after sitagliptin and exenatide were trialed and shown to be ineffective at lowering HbA1c to less than 7·0%. We were able to combine exenatide with insulin, but usually increased the insulin dose and did not prescribe exenatide if HbA1c was greater than 7·0% despite insulin therapy. Blood pressure was targeted to less than 120/80 mm Hg in participants with albuminuria or a history of cardiovascular disease, and to less than 130/80 mm Hg for others. Antihypertensive drug therapy was intensified if these targets were not achieved after 3 months of lifestyle change. Anti-platelet therapy and statins were prescribed to all participants with a history of cardiovascular disease and to those older than 40 years who had an additional cardiovascular risk factor. Annual ophthalmology review was arranged through the family physician. Drugs were weaned if JMW judged that the participant would continue to meet treatment targets, which were reassessed within 3 months of this change.<br><br>The dietitian recommended a tailored calorie-restricted diet to all participants. Multivitamin supplements were recommended to all participants in the gastric

	banding group. The sessions with a diabetes educator focused on diabetes self-management, including blood glucose monitoring and sick-day management (ie, patients are educated about the possibility of hyperglycaemia or hypoglycaemia if they develop an illness such as respiratory tract or bowel infections).<br><br>For all patients, clinical biochemistry was done by Melbourne Pathology (Collingwood, Australia). The oral glucose tolerance test to assess diabetes status was done at 2 year follow-up only if HbA1c was less than 6·5% (48 mmol/mol) and fasting blood glucose less than 7·0 mmol/L, irrespective of whether the participant was receiving antidiabetes therapy. Anthropometric data and blood pressure, measured by automated sphygmomano- meter (Philips SureSign VS2; MA, USA), were collected by a study nurse. Quality-of-life measures were assessed at baseline and at year 2 with the Short Form-36 questionnaire,18 standardised to Australian norms (Australian Bureau of Statistics Catalogue 4399.0). We recorded all adverse events prospectively, but their severity was assessed after completion of the trial by the research nurses (JP and CL), verified by contacting the relevant health practitioner or hospital, and adjudicated by the authors. There was no data safety monitoring board.
Duration of Intervention and control	I: 2 years<br>C: 2 years
Length of Follow-Up Beyond Post-Intervention	10-year follow-up.
List of Outcomes	Primary outcome:<br>Diabetes remission after 2 years.<br><br>Secondary outcomes:<br>Exploratory secondary outcomes assessed during the trial and after 2 years included weight change (waist circumference), systolic and diastolic BP, glycaemic control (fasting glucose, HbA1c), blood pressure, lipid profile, drug burden, quality of life (physical and mental well-being), and costs.<br><br>10 year outcomes (REFID-12286) - Diabetes remission HbA1c Diabetes treatment Metformin Sulphonylurea DPP- inhibitor GLP agonist SGLT inhibitor Insulin HOMA-B HOMA-IR Fasting glucose Weight Weight loss Percentage weight loss Change in waist Systolic blood pressure Diastolic blood pressure Total cholesterol Triglycerides High-density lipoprotein cholesterol Low-density lipoprotein cholesterol 10-year coronary artery disease risk score Diabetes medication cost/month Number of diabetes medications CV medication cost/month Number of CV medications Complications – total Macrovascular complications Microvascular complications Cataracts QoL physical composite score QoL mental composite score Health utility<br><br>* Abbreviations: CV, cardiovascular; DPP-4, dipeptidyl peptidase-4; GB, gastric band; GLP, glucagon-like peptide; QoL, quality of life; HbA1c, glycated haemoglobin; SGLT, sodium-glucose cotransport.

Serious Adverse Events	One patient developed a symmetrical enlargement of the stomach above the band that required revision surgery as an outpatient at week 99 of the study. Four (17%) patients in the gastric band group had a total of five episodes of food intolerance that required a reduction of the fluid volume in the band as outpatients. Five unplanned surgical procedures (knee arthroscopy, uterine curettage, inguinal hernia repair, cholecystectomy, and transurethral resection of prostate) were recorded in four (17%) participants in the gastric band group compared with one participant (knee arthroscopy) in the multidisciplinary care group. Two (8%) patients in the multidisciplinary care group needed retinal photocoagulation and one was admitted to hospital for 2 months to manage eosinophilic fasciitis, possibly precipitated by atorvastatin.
Funding Source	Monash University Centre for Obesity Research and Education and Allergan.
Comments	

**Yi et al., 2015**	
Country, Year	China, 2015
Question/Study Objective	We conducted a comparative study to assess the efficacy and side effects of 2 procedures, to determine which procedure is more suitable for Chinese T2DM patients.
Study Design	RCT
Inclusion/Exclusion Criteria	Inclusion criteria:<br>(1) a diagnosis of T2DM according to the 1999 World Health Organization (WHO) diagnostic criteria [4],<br>(2) a ratio of the peak value to the base value of C peptide release test (CPRT) 42 (the base value of CPRT divided by an oral glucose tolerance test [OGTT] result 40.33 mg/L),<br>(3) BMI r35 kg/m2, and<br>(4) age Z18 years and r65 years.<br><br>Exclusion criteria:<br>Patients with a history of open abdominal surgery; unstable psychiatric illness; an inability or reluctance to cooperate during long-term follow-up; alcohol or drug addiction; or relatively high surgical risks, such as active ulcers or Helicobacter pylori infection (detected by preoperative upper endoscopy).
Diabetes Remission Definition	The remission criterion was based on the recommendation of the international diabetes federation (IDF) (postoperative FBG o7.0 mmol/L, 2-h OGTT blood glucose levels o11.1 mmol/L, and HbA1c o6.5% without DM medications).
Sample Size	N=60<br>I=30<br>C=30
Loss to Follow-Up	N=0<br>I=0<br>C=0
Age	Mean overall (SD)= N/R<br>Mean I (SD)= 49.1(6. 2)<br>Mean C (SD)= 48.2 (8.2)
Gender	Female I: 6(20%); C: 8(26.7%)<br>Male: I: 24(80%), C: 22(73.3 %)
Race/Ethnicity	Chinese
BMI	Mean overall (SD)=N/R<br>Mean I (SD)= 26.9 (0.7)<br>Mean C (SD)= 25.7 (0.9)
Comorbidities	N/R
Duration of Diabetes	Mean overall (years SD)= N/R<br>Mean I (years SD)= 6.1 (4.7)<br>Mean C (years SD)= 5.9 (4.5)
Baseline A1C%	Mean overall (SD)= N/R<br>Mean I (SD)= 8(1.5)<br>Mean C (SD)= 8(1.2)
Description of Intervention	LRYGB with a small stomach pouch left a small stomach pouch of approximately 50 mL (the rest of the stomach was excluded), based on the lesser curvature and

	ending at the angle of His, and the alimentary and biliopancreatic limbs remained 50 cm in length.<br><br>The LRYGB with a small stomach pouch (marked with small stomach pouch); the volume of the small stomach pouch is 50 mL. The alimentary limb from end-to-side anastomosis of the stomach and distal jejunum to the second side-to-side anastomosis of the proximal jejunum and the distal jejunum is 50 cm. The biliopancreatic limb from the ligament of Treitz to the second side-to-side anastomosis of the proximal jejunum and the distal jejunum is 50 cm. The total time required for each procedure was approximately 3 hours.
Category of Intervention	RYGB<br>Gastric bypass<br>LRYGB with a small stomach pouch
Who Delivered Intervention	Surgeon
Location/Site of Delivery	Hospital
Description of Control	Laparoscopic Roux-en-Y gastrojejunostomy was specifically performed under general anesthesia using 4 trocars. The subtotal stomach (based on the greater curvature and ending at the angular incisure) and the jejunum 50 cm distal from the ligament of Treitz were then divided and closed using a linear cutting stapler. An end-to-side anastomosis connected the distal jejunum to the posterior wall of the stomach, and a second side-to-side anastomosis was performed 50 cm away from the first anastomosis and the proximal jejunum. This procedure reserved subtotal gastric volume and did not restrict food from flowing through the gastrointestinal (GI) tract. The duodenum and proximal jejunum were bypassed so that food directly entered the distal jejunum. The GI continuity was reestablished, but the content was mixed with pancreatic juice and bile, thereby leading to delayed digestion and absorption.<br><br>Laparoscopic Roux-en-Y gastrojejunostomy (marked with big stomach pouch): The alimentary limb from end-to-side anastomosis of the stomach and distal jejunum to the second side-to-side anastomosis of the proximal jejunum and the distal jejunum is 50 cm. The biliopancreatic limb from the ligament of Treitz to the second side-to-side anastomosis of the proximal jejunum and the distal jejunum is 50 cm. The total time required for each procedure was approximately 3 hours.
Duration of Intervention and control	I: 12 months<br>C: 12 months
Length of Follow-Up Beyond Post-Intervention	N/A
List of Outcomes	1. BMI (kg/m2)<br>2. Body fat rate (%)<br>3. HOMA-IR<br>4. Waist circumference<br>5. HbA1c (%)<br>6. FPG<br>7. PPG

	8. Fasting C peptide<br>9. C peptide 2 hr
Serious Adverse Events	No significant surgery-related complications occurred in the patients who underwent Roux-en-Y with a small stomach pouch, except for 2 cases of marginal ulcers (6.7%, 2/30) in which the patients recovered with conservative treatment. However, postoperative complications, such as marginal ulcers, were observed in 8 patients (26.7%, 8/30) in the Roux-en-Y gastrojejunostomy group, even though 2 of them, who manifested gastrointestinal hemorrhage, all marginal-ulcer patients in both groups were treated with 8-week proton pump inhibitor as conservative treatment
Funding Source	N/R<br>“The study was conducted in compliance with a protocol approved by the Ethics Committee of the Third Xiangya Hospital at Central South University”
Comments	

Supplementary File 3: TIDieR Tool Items

Cheng et al., 2022		
**TIDieR Tool Item**	**Main Paper**	**Other Paper(s)**
**BRIEF NAME**<br>Provide the name or a phrase that describes the intervention.	RYGB versus best medical treatment in Asians with type 2 diabetes mellitus (T2DM) and class I obesity	
**WHY**<br>Describe any rationale, theory, or goal of the elements essential to the intervention.	Emerging but limited data from randomized clinical trials comparing bariatric surgery with lifestyle/medical treatment for T2DM have supported surgical superiority in treating diabetes among patients with mild obesity. However, current evidence relevant to Asian populations is scarce. This randomized controlled trial aimed to assess the durability of metabolic control and weight loss conferred by RYGB versus best medical treatment (including treatment with GLP1RA and SGLT2i) in a multi-ethnic Asian cohort with T2DM and BMI between 27–32 kg/m^2 up to 5 years.	
**WHAT**<br>Materials: Describe any physical or informational materials used in the intervention, including those provided to participants or used in intervention delivery or in training of intervention providers.<br>Provide information on where the materials can be accessed (e.g., online appendix, URL).	N/R	

Procedures: Describe each of the procedures, activities, and/or processes used in the intervention, including any enabling or support activities.	RYGB: Initial preoperative workup included, but was not limited to, consultation with surgeon, diabetologist, dietitian, physiotherapist, and psychologist; blood and urine tests, upper endoscopy, colonoscopy for over 50 years of age or otherwise indicated, sleep study, and abdominal ultrasound. Subjects underwent standard laparoscopic RYGB. Oral medication was then reintroduced after adequate oral fluid was tolerated. In addition, visits by diabetologist, dietitian, and physiotherapist were conducted on day 1 post-surgery. Subjects were discharged the morning of post-operative day 2. Study visits were scheduled at week 2 and week 6 after surgery, then at 3, 6, 9, 12 months, then at 6-months intervals until the program ends. Each visit includes surgeon and diabetologist consult. Dietary and lifestyle intervention were more regular initially. Other visits were scheduled according to needs, with referral to other services as indicated.	
**WHO PROVIDED** For each category of intervention provider (e.g., psychologist, nursing assistant), describe their expertise, background and any specific training given.	Initial preoperative workup included, but was not limited to, consultation with surgeon, diabetologist, dietitian, physiotherapist, and psychologist. Visits with diabetologist, dietitian, and physiotherapist post-surgery. Ongoing study visits with surgeon and diabetologist.	
**HOW** Describe the modes of delivery (e.g., face-to-face or by some other mechanism,	N/R	

such as internet or telephone) of the intervention and whether it was provided individually or in a group.		
**WHERE**<br>Describe the type(s) of location(s) where the intervention occurred, including any necessary infrastructure or relevant features.	Tertiary hospital	
**WHEN and HOW MUCH**<br>Describe the number of times the intervention was delivered and over what period of time including the number of sessions, their schedule, and their duration, intensity or dose.	Study visits were scheduled at week 2 and week 6 after surgery, then at 3, 6, 9, 12 months, then at 6-months intervals until the program ends.	
**TAILORING**<br>If an intervention was planned to be personalized, titrated or adapted, then describe what, why, when, and how.	N/R for RYGB group	
**MODIFICATION**<br>If an intervention was modified during the study, describe the changes (what, why, when, and how).	N/R	
**HOW WELL**<br>Planned: If intervention adherence or fidelity	N/R	

was assessed, describe how and by whom, and if any strategies were used to maintain or improve fidelity, describe them.		
Actual: If intervention adherence or fidelity was assessed, describe the extent to which the intervention was delivered as planned.	Poor participant recruitment is a well-recognized challenge due to a variety of reasons in investigator-initiated clinical trials [37]. Missed appointments at a diabetes clinic in a Singapore public institution was reported to be 53.7% in one study [38]. Non-adherence with follow-up appointments post- bariatric surgery is also high for most bariatric surgery programs [39,40]. Ours is no different, with a follow-up default rate of 55% at 3 years (unpublished data). Hence, the high attrition rate reported here simply reflects a real-world setting. While the extent of loss to follow-up in our trial is in keeping with others, we acknowledge that the likely non-random loss to follow-up may lead to bias in a direction that is challenging to evaluate accurately in our study.	

Courcoulas et al., 2014		
**TIDieR Tool Item**	**Main Paper**	**Other Paper(s)**
**BRIEF NAME**<br>Provide the name or a phrase that describes the intervention.	A Randomized Trial to Compare Surgical and Medical Treatments for Type 2 Diabetes.	
**WHY**<br>Describe any rationale, theory, or goal of the elements essential to the intervention.	Address unanswered questions about the role of bariatric surgery for people with diabetes.<br><br>Determine feasibility of a randomized controlled trial (RCT) and compare initial outcomes of bariatric surgery and a structured weight loss program for treating type 2 diabetes mellitus in grade 1 and 2 obese participants.<br><br>Primary outcomes in the intention to treat (ITT) cohort were feasibility and effectiveness measured by weight loss and improvements in glycemic control.	
**WHAT**<br>Materials: Describe any physical or informational materials used in the intervention, including those provided to participants or used in intervention delivery or in training of intervention providers.<br>Provide information on where the materials can be accessed (e.g., online appendix, URL).	N/R	
Procedures: Describe each of the	The RYGB was performed with a standard retrocolic, retrogastric	Briefly, the trial was a 3-arm RCT stratified by sex and

procedures, activities, and/or processes used in the intervention, including any enabling or support activities.	technique using a linear stapled and hand sewn gastrojejunal anastomosis. Surgical participants underwent clinical follow-up assessments consistent with current practice: RYGB at 2 weeks, 3 months, 6 months, 9 months, and 12 months post-operatively, LAGB at 2 weeks, and at 2, 4,6, 8, 10 and 12 months or more frequently, as necessary, for band adjustment using a standard clinical assessment and questionnaire. Those undergoing surgical intervention were counseled on a diet program consistent with post-bariatric surgery recommendations and were encouraged to exercise a minimum of 3–4 times per week and to focus on weightbearing, aerobic activity Year 1.<br><br>Subjects randomized to undergo the LWLI underwent a standard behavioral weight control program delivered in an in-person, individual, format based on the intervention developed for the Diabetes Prevention Program (DPP)20 and the Look AHEAD Study21 and adapted into a 12-month program for subjects with grade 1–2 obesity. During the initial 6 months of treatment, LWLI participants attended weekly in-person intervention sessions. During months 7–12, they attended in person sessions on the 1st and 3rd week of the month and received brief telephone contacts on the 2nd and 4th weeks. Each session focused on a specific behavioral topic related to weight loss, eating or exercise behaviors. Participants were provided supplemental written	baseline BMI, conducted at an academic medical center, that compared the efficacy for treating T2DM of 2 common surgical procedures (RYGB and LAGB) plus LLLI in years 2 and 3 of follow-up with intensive LWLI in year 1 followed by 2 years of LLLI.<br><br>At years 2 and 3, later low-level (LLLI)lifestyle interventions<br><br>An initial instructional group session was held for participants in both surgical arms (RYGB and LAGB) to provide a lesson on behavioral weight control and orient them to the skills and strategies that had been learned and developed for the LWLI group in year 1. The LLLI for all 3 treatment groups consisted of twice-monthly contact (1 in-person session [approximately 30-45 minutes] and 1 brief [<10 minutes] telephone contact) and regular refresher group series.13,14 Each intervention contact focused on a specific behavioral topic related to weight loss. If an individual missed an in-person session, all intervention materials were mailed to the participant. If a participant became unable to attend monthly in-person LLLI sessions, they received intervention telephone calls in place of visits.

	materials and were asked to self-monitor body weight, eating, and exercise. All LWLI participants were prescribed an energy restricted diet (12001800 kcal/day) and were provided meal plans, meal replacements, and calorie-counter books. Moderate-intensity, 5-day-per-week exercise was prescribed beginning at 20 minutes per day and gradually progressing to at least 60 minutes per day, bouts of activity encouraged to be >10 minutes in duration.	
**WHO PROVIDED** For each category of intervention provider (e.g., psychologist, nursing assistant), describe their expertise, background and any specific training given.	Each participant underwent individual evaluation by the surgeon and after successful medical, nutritional and psychological assessments were completed.	
**HOW** Describe the modes of delivery (e.g., face-to-face or by some other mechanism, such as internet or telephone) of the intervention and whether it was provided individually or in a group.	In-person	Initial informational session was group for the lifestyle intervention started in year 2.
**WHERE** Describe the type(s) of location(s) where the intervention occurred, including any necessary infrastructure or relevant features.	Academic medical center (hospital; tertiary)	

**WHEN and HOW MUCH** Describe the number of times the intervention was delivered and over what period of time including the number of sessions, their schedule, and their duration, intensity or dose.	For surgical group, no other details on length of visits or other sessions.<br><br>Surgical participants underwent clinical follow-up assessments consistent with current practice: RYGB at 2 weeks, 3 months, 6 months, 9 months, and 12 months post-operatively, LAGB at 2 weeks, and at 2, 4, 6, 8, 10 and 12 months or more frequently, as necessary, for band adjustment using a standard clinical assessment and questionnaire.	The LLLI for all 3 treatment groups consisted of twice-monthly contact (1 in-person session [approximately 30-45 minutes] and 1 brief [<10 minutes] telephone contact) and regular refresher group series. If a participant became unable to attend monthly in-person LLLI sessions, they received intervention telephone calls in place of visits.
**TAILORING** If an intervention was planned to be personalized, titrated or adapted, then describe what, why, when, and how.	Patients were able to attend the clinic more frequently if needed based on clinical assessment.	individualized follow-up program
**MODIFICATION** If an intervention was modified during the study, describe the changes (what, why, when, and how).	N/R	
**HOW WELL** Planned: If intervention adherence or fidelity was assessed, describe how and by whom, and if any strategies were used to maintain or improve fidelity, describe them.	N/R	
Actual: If intervention adherence or fidelity was assessed, describe the extent	N/R	

to which the intervention was delivered as planned.		

Cummings et al., 2016		
**TIDieR Tool Item**	**Main Paper**	**Other Paper(s)**
**BRIEF NAME**<br>Provide the name or a phrase that describes the intervention.	The CROSSROADS randomized controlled trial.<br><br>CROSSROADS = (Calorie Reduction Or Surgery: Seeking to Reduce Obesity And Diabetes Study)	
**WHY**<br>Describe any rationale, theory, or goal of the elements essential to the intervention.	we conducted a prospective RCT comparing RYGB to an intensive lifestyle and medical intervention (ILMI), including aggressive, supervised dieting and exercise, modelled after the Diabetes Prevention Program (DPP) [24] and LookAHEAD trials [25].<br><br>Our novel recruitment methods used a shared decision-making (SDM) approach to identify, screen, educate and randomise all adults who demonstrated equipoise between surgical and lifestyle treatment of obesity and diabetes in a large, integrated healthcare delivery system	
**WHAT**<br>Materials: Describe any physical or informational materials used in the intervention, including those provided to participants or used in intervention delivery or in training of intervention providers.<br>Provide information on where the materials can be accessed (e.g., online appendix, URL).	N/R	

Procedures: Describe each of the procedures, activities, and/or processes used in the intervention, including any enabling or support activities.	Participants randomized to surgery underwent a laparoscopic proximal RYGB, using an estimated 40 ml gastric pouch, 100–150 cm alimentary limb, a biliopancreatic limb that included 30–50 cm of jejunum beyond the ligament of Treitz, an antecolic/antegastric approach, and combined stapled and sutured technique. Surgical patients also underwent a 4-week pre-operative and 10-month postoperative behavioral treatment regimen. In the pre-operative phase, patients had weekly telephone-based appointments with a health educator and were required to attend 2–3 bariatric support group meetings. Patients continued to have phone appointments with their health educator for 10 months after surgery. The postoperative behavioural treatment program focused on diet and nutrition counselling, behaviour modification and exercise recommendations.	
**WHO PROVIDED**<br>For each category of intervention provider (e.g., psychologist, nursing assistant), describe their expertise, background and any specific training given.	Investigators at the University of Washington, Group Health Research Institute (GHRI), and Fred Hutchinson Cancer Research Center (FHCRC). A surgeon provided initial screening of potential participants, reviewing charts. A study physician obtained in-person, written informed consent. A health educator for telephone-based appointments pre-op and during follow-up	
**HOW**<br>Describe the modes of delivery (e.g., face-to-face or by some other mechanism, such as internet or telephone) of the intervention and	In-person and telephone-based program. Individual and group components<br><br>In the pre-operative phase, patients had weekly telephone-based appointments with a health educator and were required to attend 2–3	

whether it was provided individually or in a group.	bariatric support group meetings. Patients continued to have phone appointments with their health educator for 10 months after surgery.	
**WHERE**<br>Describe the type(s) of location(s) where the intervention occurred, including any necessary infrastructure or relevant features.	N/R	
**WHEN and HOW MUCH**<br>Describe the number of times the intervention was delivered and over what period of time including the number of sessions, their schedule, and their duration, intensity or dose.	4-week pre-operative: weekly telephone-based appointments with a health educator and were required to attend 2–3 bariatric support group meetings<br><br>10-month postoperative behavioural treatment regimen: phone appointments with their health educator for 10 months after surgery.	
**TAILORING**<br>If an intervention was planned to be personalized, titrated or adapted, then describe what, why, when, and how.	N/R	
**MODIFICATION**<br>If an intervention was modified during the study, describe the changes (what, why, when, and how).	N/R	
**HOW WELL**<br>Planned: If intervention adherence or fidelity was assessed,	N/R	

describe how and by whom, and if any strategies were used to maintain or improve fidelity, describe them.		
Actual: If intervention adherence or fidelity was assessed, describe the extent to which the intervention was delivered as planned.	N/R	

Dixon et al., 2008		
**TIDieR Tool Item**	**Main Paper**	**Other Paper(s)**
**BRIEF NAME**<br>Provide the name or a phrase that describes the intervention.	Adjustable Gastric Banding and Conventional Therapy for Type 2 Diabetes	
**WHY**<br>Describe any rationale, theory, or goal of the elements essential to the intervention.	To determine if surgically induced weight loss results in better glycemic control and less need for diabetes medications than conventional approaches to weight loss and diabetes control.<br><br>Using the LAGB intervention, we conducted a 2-year randomized controlled trial involving 60 obese participants (BMI 30 and 40) to compare surgically induced weight loss with conventional therapy for the management of recently diagnosed type 2 diabetes (2 years).	
**WHAT**<br>Materials: Describe any physical or informational materials used in the intervention, including those provided to participants or used in intervention delivery or in training of intervention providers.<br>Provide information on where the materials can be accessed (e.g., online appendix, URL).	N/R	
Procedures: Describe each of the procedures,	Each potential participant was assessed by a dietitian, a general physician, and a consultant	

activities, and/or processes used in the intervention, including any enabling or support activities.	endocrinologist specializing in diabetes (L.C.) to suggest any changes required to maximize current management. A run-in period of at least 3 months was undertaken in which further alterations to eating, exercise, glucose self-monitoring, and medications were suggested.<br><br>This program delivered the best available medical practice for the treatment, education, and follow-up of patients with type 2 diabetes. Patients had open access to a general physician, dietitian, nurse, and diabetes educator and had visits with at least 1 team member every 6 weeks throughout the 2 years. Medical therapies, including pharmaceutical agents, were determined by an experienced diabetologist on an individual basis. Lifestyle modification programs were individually structured to reduce energy intake, to reduce intake of fat (30%) and saturated fats, and to encourage intake of low glycemic index and high-fiber foods. Physical activity advice encouraged 10000 steps per day and 200 minutes per week of structured activity, including moderate intensity aerobic activity and resistance exercise. Lifestyle was the primary approach to weight loss, but very low calorie diets and medications were discussed with all patients and used after consultation with the dietitian or general physician if the patient expressed a desire to use additional measures.<br><br>Laparoscopic adjustable gastric band progress was reviewed by the bariatric surgical team every 4 to 6	

	weeks throughout the study, and adjustments to band volume were made using standard clinical criteria	
**WHO PROVIDED**<br>For each category of intervention provider (e.g., psychologist, nursing assistant), describe their expertise, background and any specific training given.	General physician, dietitian, nurse, and diabetes educator. Diabetologist and endocrinologist.	
**HOW**<br>Describe the modes of delivery (e.g., face-to-face or by some other mechanism, such as internet or telephone) of the intervention and whether it was provided individually or in a group.	N/R	
**WHERE**<br>Describe the type(s) of location(s) where the intervention occurred, including any necessary infrastructure or relevant features.	N/R	
**WHEN and HOW MUCH**<br>Describe the number of times the intervention was delivered and over what period of time including the number of sessions, their schedule, and their duration, intensity or dose.	Visits with at least 1 team member every 6 weeks throughout the 2 years<br><br>Progress was reviewed by the bariatric surgical team every 4 to 6 weeks throughout the study	

**TAILORING**<br>If an intervention was planned to be personalized, titrated or adapted, then describe what, why, when, and how.	Each potential participant was assessed by a dietitian, a general physician, and a consultant endocrinologist specializing in diabetes (L.C.) to suggest any changes required to maximize current management. A run-in period of at least 3 months was undertaken in which further alterations to eating, exercise, glucose self-monitoring, and medications were suggested.<br><br>Medical therapies, including pharmaceutical agents, were determined by an experienced diabetologist on an individual basis.<br><br>Lifestyle modification programs were individually structured	
**MODIFICATION**<br>If an intervention was modified during the study, describe the changes (what, why, when, and how).	N/R	
**HOW WELL**<br>Planned: If intervention adherence or fidelity was assessed, describe how and by whom, and if any strategies were used to maintain or improve fidelity, describe them.	Study compliance was assessed using attendance at appointments and completion of questionnaires.	
Actual: If intervention adherence or fidelity was assessed, describe the extent to which the intervention was delivered as planned.	N/R (only length of surgery)	

Fernandez-Soto et al., 2017		
**TIDieR Tool Item**	**Main Paper**	**Other Paper(s)**
**BRIEF NAME**<br>Provide the name or a phrase that describes the intervention.	Comparison of remission of type 2 diabetes mellitus after mixed and restrictive bariatric surgery	
**WHY**<br>Describe any rationale, theory, or goal of the elements essential to the intervention.	In this work, we seek to find out the short- and midterm effectiveness of one restrictive technique (LSG) in type 2 diabetes mellitus improvement, weight loss, and related co-morbidities progress, in comparison with two mixed procedures (RYGB and BPD), after one year of follow-up [11].<br><br>Furthermore, we try to identify reliable clinical baseline predictors of the likelihood of type 2 diabetes mellitus remission after these different<br>surgical procedures.<br><br>This is a prospective and observational study of co-morbid, type 2 diabetes mellitus evolution in 49 morbidly obese patients: 37 underwent mixed surgery procedures and 12 a restrictive surgery procedure	
**WHAT**<br>Materials: Describe any physical or informational materials used in the intervention, including those provided to participants or used in intervention delivery or in training of intervention providers.	N/R	

Provide information on where the materials can be accessed (e.g., online appendix, URL).		
Procedures: Describe each of the procedures, activities, and/or processes used in the intervention, including any enabling or support activities.	Four weeks before surgery, patients followed a hypocaloric diet with 800 Kcal/day and 56 g protein/day intake. After the operation, multivitamin and mineral supplements were prescribed in patients who underwent the mixed procedures, to minimise micronutrient deficiency [12]. Nonpregnant and non-lactating female patients had to agree to use a reliable method of contraception for two years.<br><br>We conducted three different bariatric techniques grouped in two types of surgical procedures: mixed and restrictive. Mixed procedures have a restrictive and malabsorptive action. Biliopancreatic diversion Scopinaro and Roux-en-Y Gastric Bypass, which we performed as mixed procedures,<br><br>As a restrictive procedure, we conducted laparoscopic sleeve gastrectomy.	
**WHO PROVIDED**<br>For each category of intervention provider (e.g., psychologist, nursing assistant), describe their expertise, background and any specific training given.	Surgeon<br><br>Progress of all patients after surgery weas controlled by a multidisciplinary team at the Surgery and Nutrition Unit, Endocrinology and Surgery Department.	
**HOW**<br>Describe the modes of delivery (e.g., face-	N/R	

to-face or by some other mechanism, such as internet or telephone) of the intervention and whether it was provided individually or in a group.		
**WHERE**<br>Describe the type(s) of location(s) where the intervention occurred, including any necessary infrastructure or relevant features.	Clinic hospital<br><br>Progress of all patients after surgery was controlled by a multidisciplinary team at the Surgery and Nutrition Unit, Endocrinology and Surgery Department, Clinic Hospital San Cecilio (Granada, Spain).	
**WHEN and HOW MUCH**<br>Describe the number of times the intervention was delivered and over what period of time including the number of sessions, their schedule, and their duration, intensity or dose.	Surgery was one-time intervention. No other visits reported. Data recorded at baseline, six and twelve months post-surgical intervention.	
**TAILORING**<br>If an intervention was planned to be personalized, titrated or adapted, then describe what, why, when, and how.	N/R	
**MODIFICATION**<br>If an intervention was modified during the study, describe the changes (what, why, when, and how).	N/R	
**HOW WELL**	N/R	

Planned: If intervention adherence or fidelity was assessed, describe how and by whom, and if any strategies were used to maintain or improve fidelity, describe them.		
Actual: If intervention adherence or fidelity was assessed, describe the extent to which the intervention was delivered as planned.	N/R	

Hofso et al., 2019		
**TIDieR Tool Item**	**Main Paper**	**Other Paper(s)**
**BRIEF NAME**<br>Provide the name or a phrase that describes the intervention.	Gastric bypass versus sleeve gastrectomy for type 2 diabetes	
**WHY**<br>Describe any rationale, theory, or goal of the elements essential to the intervention.	Aimed to compare the two most common procedures, hypothesizing higher remission rates of diabetes after gastric bypass than after sleeve gastrectomy.<br><br>The Oseberg study is an ongoing triple-blind, randomized, single-center trial.	
**WHAT**<br>Materials: Describe any physical or informational materials used in the intervention, including those provided to participants or used in intervention delivery or in training of intervention providers.<br>Provide information on where the materials can be accessed (e.g., online appendix, URL).	N/R	
Procedures: Describe each of the procedures, activities, and/or processes used in the intervention, including any enabling or support activities.	The two intervention groups received identical pre- and post-operative treatment, including a low-calorie diet (<1200 kcal/day) during the two weeks preceding surgery. All patients will be encouraged to normalize their eating behavior and to increase their physical activity level. Antidiabetic and antihypertensive medication, statin	

	therapy, the management of reflux disease and vitamin and mineral supplementations, were adjusted according to specific predefined algorithms (appendix pp 7, 8). Patients were informed about healthy diets and physical activity, and the medical treatment was in accordance with international guidelines.<br>Pre-operative antibiotic prophylaxis with oral trimetoprim-sulpha before surgery will be used.<br><br>The surgical procedures were performed laparoscopically.<br><br>All patients will follow standard post-operative care with observation in a post-operative ward the first few hours after surgery with early mobilization before transfer to the gastrointestinal surgery ward. Patients will be observed in a gastrointestinal surgery ward and visited daily by a bariatric surgeon.<br><br>On the day of the operation patients will not be allowed to drink. The first week after surgery clear liquids followed by full liquid intake are allowed. Patients will be allowed mashed food during week 2 and 3 post-surgery and be encouraged to gradually eat normal food thereafter. During surgery and hospitalization compression stockings grade II will be used. Low molecular heparin will be given 6 hours after surgery and daily for 14 days, unless there is an indication that longer treatment is required. Patients will be submitted from hospital 1-3 days after surgery depending on the distance to their home. All patients will receive a	

	prescription for a proton pump inhibitor to be used post-operatively for 4 weeks.	
**WHO PROVIDED**<br>For each category of intervention provider (e.g., psychologist, nursing assistant), describe their expertise, background and any specific training given.	All procedures were performed by at least one of four experienced bariatric surgeons, all of whom are certified specialists in gastrointestinal surgery.<br><br>Trained nurse/research assistant.<br><br>Physician consultations.<br><br>Skilled anesthetists familiar with obesity surgery.	
**HOW**<br>Describe the modes of delivery (e.g., face-to-face or by some other mechanism, such as internet or telephone) of the intervention and whether it was provided individually or in a group.	Face-to-face follow-up visits	Telephone follow-up for 3 patients
**WHERE**<br>Describe the type(s) of location(s) where the intervention occurred, including any necessary infrastructure or relevant features.	Tertiary health care resource center	
**WHEN and HOW MUCH**<br>Describe the number of times the intervention was delivered and over what period of time including the number of sessions, their schedule, and their	N/R beyond assessment/outcome measurement visits.<br><br>3 weeks prior to (baseline) and 5, 16, 34 and 52 weeks after randomization and thereafter annually for four more years.<br><br>All visits will include a clinical examination (body weight, anthropometric measures and blood	

duration, intensity or dose.	pressure), laboratory analyses and a registration of supplementation and medication used.<br><br>Additional data will be collected four times during the study period (three weeks prior to and five weeks and one and five years after randomization).	
**TAILORING**<br>If an intervention was planned to be personalized, titrated or adapted, then describe what, why, when, and how.	Antidiabetic and antihypertensive medication, statin therapy, the management of reflux disease and vitamin and mineral supplementations, were adjusted according to specific predefined algorithms at each data collection point.	
**MODIFICATION**<br>If an intervention was modified during the study, describe the changes (what, why, when, and how).	Iron supplementation changed from 65 mg for men and 130 mg for all women to 100 mg for pre-menopausal women only (Aug 2013).<br><br>The five-year visit was extended to include all examinations included at the one year visit (Jan 2018).<br><br>SGLT2-inhibitors (anti-diabetic drug initially regarded as a third-line treatment), were now considered a second-line medication (Feb 2019).	One patient who underwent sleeve gastrectomy was converted to gastric bypass because of insufficient weight loss, whereas one gastric bypass patient was re-operated because of small bowel obstruction.
**HOW WELL**<br>Planned: If intervention adherence or fidelity was assessed, describe how and by whom, and if any strategies were used to maintain or improve fidelity, describe them.	Protocol violations will be registered at every study visit.<br><br>In the case of low vitamin levels after surgery, the first step is to ensure the patient is taking the supplements. Thereafter supplementation of vitamin and mineral deficiencies will follow predefined regimes.<br><br>Seven authors (DH, FF, HB, JKH, LKJ, MSC and JH) had independent access to the data, with all authors vouching for data completeness, accuracy and	

	for the fidelity of the trial to the protocol.	
Actual: If intervention adherence or fidelity was assessed, describe the extent to which the intervention was delivered as planned.	Adherence to prescribed vitamin and mineral supplementations was high in both groups (Table 13).	

Ikramuddin et al., 2016		
**TIDieR Tool Item**	**Main Paper**	**Other Paper(s)**
**BRIEF NAME**<br>Provide the name or a phrase that describes the intervention.	Durability of Addition of Roux-en-Y Gastric Bypass to Lifestyle Intervention and Medical Management in Achieving Primary Treatment Goals for Uncontrolled Type 2 Diabetes in Mild to Moderate Obesity	Roux-en-Y gastric bypass for diabetes (2015)
**WHY**<br>Describe any rationale, theory, or goal of the elements essential to the intervention.	To compare 3-year achievement of an American Diabetes Association composite treatment goal (HbA1c <7.0%, LDL cholesterol <100 mg/dL, and systolic blood pressure <130 mmHg) after 2 years of intensive lifestyle-medical management intervention, with and without Roux-en-Y gastric bypass, with one additional year of usual care.<br><br>This randomized controlled trial was designed to provide 2 years of intense lifestyle and medical management in all subjects with three additional years of observation while on usual medical care.<br><br>The 2-year lifestyle intervention was based on protocols from two successful clinical trials: the Diabetes Prevention Program (DPP) and the Look AHEAD (Action for Health in Diabetes) study.	
**WHAT**<br>Materials: Describe any physical or informational materials used in the intervention, including those provided to participants or used in intervention	Each subject’s primary physician received a letter describing the study, the subject’s current status, medications, and goals of care. The primary physicians also received recommendations about medications and information about the need for nutritional supplementation as appropriate.	

delivery or in training of intervention providers.<br>Provide information on where the materials can be accessed (e.g., online appendix, URL).		
Procedures: Describe each of the procedures, activities, and/or processes used in the intervention, including any enabling or support activities.	Gastric bypass was laparoscopically performed in a standardized fashion with construction of a 20-mL lesser curvature gastric pouch and a 100-cm biliopancreatic limb.<br><br>The 2-year lifestyle intervention was based on protocols from two successful clinical trials: the Diabetes Prevention Program (DPP) and the Look AHEAD (Action for Health in Diabetes) study.<br><br>The intensive medical management protocol for both treatment groups aimed to optimize drug therapy to control hyperglycemia, cholesterol, and hypertension.	Lifestyle and medical management plus a Roux-en-Y gastric bypass.<br><br>Participants were instructed to weigh themselves and record eating and exercise behaviors daily and advised to progressively increase their amount of moderate-intensity physical activity (such as walking) to 325 min per week. Participants met regularly with a dietitian or registered nurse to discuss strategies for weight management and increasing physical activity, including self-monitoring, stimulus control, problem-solving, social support, cognitive behavior modification, recipe modification, eating away from home, and relapse prevention.<br><br>Medicines for glycemic control were added or reintroduced in the following order: metformin, a glucagon-like peptide-1 agonist or dipeptidyl peptidase-4 inhibitor, sulfonylurea or pioglitazone, and insulin. We pursued

		control of LDL cholesterol with 3-hydroxy-3-methylglutaryl-coenzyme A (HMG-CoA) reductase inhibitors first, then ezetimibe if necessary. Blood pressure medicines were used in the following order: angiotensin-converting enzyme inhibitors or angiotensin receptor II blockers, diuretics, β blockers, and additional agents as necessary. If triglyceride concentrations remained higher than 3·39 mmol/L after hyperglycemia was controlled, fenofibrate or fish oil were added to participants' diets. Smoking cessation was strongly recommended for all. An angiotensin converting enzyme inhibitor or angiotensin receptor II blocker was provided for participants with microalbuminuria or macro-albuminuria. Aspirin (81–100 mg daily) was added, consistent with evolving recommendations from the ADA, when not contra indicated.<br><br>Medicines approved by the US Food and Drug Administration for long-term obesity treatment were used. By protocol, participants in the gastric bypass group were prescribed multivitamins and other supplements, including calcium, iron, vitamin D, and

		vitamin B12, irrespective of routine test results, to prevent nutritional deficiencies. All patients received routine testing for nutritional deficiencies and supplements were adjusted as necessary.<br><br>The Roux-en-Y surgical technique was standardized across all sites and done with construction of a 20 mL lesser curvature gastric pouch, and a 100 cm biliopancreatic limb.<br><br>The technical skill of each surgeon was established by personal observation of the principal surgeon. The study surgeons performed all postoperative surgical interventions.
**WHO PROVIDED**<br>For each category of intervention provider (e.g., psychologist, nursing assistant), describe their expertise, background and any specific training given.	Surgeon, endocrinologist, study coordinator.<br><br>Additionally, after 24 months: primary physician (usual care)	
**HOW**<br>Describe the modes of delivery (e.g., face-to-face or by some other mechanism, such as internet or telephone) of the intervention and whether it was	In-person (clinic visits).	

provided individually or in a group.		
**WHERE**<br>Describe the type(s) of location(s) where the intervention occurred, including any necessary infrastructure or relevant features.	A university and 4 hospitals from 2 different countries (2x2) (tertiary). Academic and research hospitals.<br><br>After 24 months: primary	
**WHEN and HOW MUCH**<br>Describe the number of times the intervention was delivered and over what period of time including the number of sessions, their schedule, and their duration, intensity or dose.	During the third year: The study coordinator contacted participants at 30 months to maintain their connection with the study and to obtain interim data on adverse events. Study endocrinologist's clinic visit at 36 months.	Counselling sessions consisted of 24 meetings (one per week) during the first 6 months, one meeting every 2 weeks between months 7 and 9, one meeting per month between months 10 and 15, then one meeting every 3 months either up to 24 months or until a total of 40 modules were completed. The lifestyle and medical management intervention protocol for 12–24 months was similar in both treatment groups.<br><br>Visits with an endocrinologist took place each month for 6 months, then every 3 months (or monthly if not at goal) for the next 6 months, then every 3 months through the second year.
**TAILORING**<br>If an intervention was planned to be personalized, titrated or adapted, then describe what, why, when, and how.		Medications were tailored to participant lab values and blood pressure.
**MODIFICATION**		Minor modifications were made to the interventions in

If an intervention was modified during the study, describe the changes (what, why, when, and how).		the USA versus Taiwan to account for differences in language and culture.<br><br>A total of 11 participants crossed over (Figure 1). Two participants randomized to gastric bypass declined surgery but participated in the rest of the study. Nine participants randomized to the lifestyle–medical management group elected gastric bypass an average of 2 years after randomization and remained in the study.
**HOW WELL**<br>Planned: If intervention adherence or fidelity was assessed, describe how and by whom, and if any strategies were used to maintain or improve fidelity, describe them.	Participants were encouraged to increase medication compliance, nutritional supplementation, and dietary control if adherence was deemed an issue.	All surgeons committed to following the surgical protocol, which was reviewed at an onsite meeting. The technical skill of each surgeon was assessed by personal observation of the principal surgeon. The study surgeons did all postoperative surgical interventions.
Actual: If intervention adherence or fidelity was assessed, describe the extent to which the intervention was delivered as planned.		Despite protocol requirements for supplementation, we were often not able to correct deficiencies with mineral and vitamin supplements, perhaps because of poor (participant) compliance.

Kehagias et al., 2023		
**TIDieR Tool Item**	**Main Paper**	**Other Paper(s)**
**BRIEF NAME**<br>Provide the name or a phrase that describes the intervention.	Diabetes Remission After LRYGBP With and Without Fundus Resection: A Randomized Clinical Trial	
**WHY**<br>Describe any rationale, theory, or goal of the elements essential to the intervention.	"...the modification of metabolic LRYGBP with fundus resection, in patients with obesity and T2DM, may appear as a metabolic weapon in the armamentarium of bariatric surgeon by ensuring optimized diabetic control through overwhelming activation of neuroendocrine mechanisms."	
**WHAT**<br>Materials: Describe any physical or informational materials used in the intervention, including those provided to participants or used in intervention delivery or in training of intervention providers.<br>Provide information on where the materials can be accessed (e.g., online appendix, URL).	N/R	
Procedures: Describe each of the procedures, activities, and/or processes used in the intervention, including any enabling or support activities.	Description of surgical procedure provided in manuscript.<br><br>The participants were not allowed to drink or eat for 6 h prior to blood collection. Preoperatively antidiabetic medications were terminated for 48 h and GLP-1 analogues for one week. Blood sampling was carried out during an 75gr OGTT (0, 30, 60, 120min)	

	preoperatively, at 6 and 12 months postoperatively.	
**WHO PROVIDED**<br>For each category of intervention provider (e.g., psychologist, nursing assistant), describe their expertise, background and any specific training given.	Surgeon at the surgical department of the University Hospital of Patras	
**HOW**<br>Describe the modes of delivery (e.g., face-to-face or by some other mechanism, such as internet or telephone) of the intervention and whether it was provided individually or in a group.	N/R	
**WHERE**<br>Describe the type(s) of location(s) where the intervention occurred, including any necessary infrastructure or relevant features.	Surgery Department of the University Hospital of Patras, in Greece	
**WHEN and HOW MUCH**<br>Describe the number of times the intervention was delivered and over what period of time including the number of sessions, their schedule, and their duration, intensity or dose.	Beyond surgery and outcome measurements, no other details were provided.<br><br>Fasting values of glycemic parameters and gastrointestinal hormones in each group preoperatively, at 6 and 12 months after surgery (e.g., HbA1c, C-peptide, HOMA-IR, glucose, insulin, GLP-1, PYY, and Ghrelin)	

**TAILORING**<br>If an intervention was planned to be personalized, titrated or adapted, then describe what, why, when, and how.	N/R	
**MODIFICATION**<br>If an intervention was modified during the study, describe the changes (what, why, when, and how).	N/R	
**HOW WELL**<br>Planned: If intervention adherence or fidelity was assessed, describe how and by whom, and if any strategies were used to maintain or improve fidelity, describe them.	N/R	
Actual: If intervention adherence or fidelity was assessed, describe the extent to which the intervention was delivered as planned.	Neither intraoperative complications nor conversion to open were reported.	

Lee et al., 2011		
**TIDieR Tool Item**	**Main Paper**	**Other Paper(s)**
**BRIEF NAME**<br>Provide the name or a phrase that describes the intervention.	Gastric Bypass vs Sleeve Gastrectomy for Type 2 Diabetes Mellitus	
**WHY**<br>Describe any rationale, theory, or	This study aims to evaluate the efficacy of 2 different gastro- intestinal metabolic operations (gastric bypass vs	

goal of the elements essential to the intervention.	sleeve gastrectomy) for the treatment of T2DM and to test the foregut hypothesis.<br><br>Foregut hypothesis: Changes in gastrointestinal hormone secretion would favor an early improvement of T2DM in GB surgery that bypasses the duodenum and upper jejunum	
**WHAT**<br>Materials: Describe any physical or informational materials used in the intervention, including those provided to participants or used in intervention delivery or in training of intervention providers.<br>Provide information on where the materials can be accessed (e.g., online appendix, URL).	N/R	
Procedures: Describe each of the procedures, activities, and/or processes used in the intervention, including any enabling or support activities.	Surgery was performed with the patients under general anesthesia in the reversed Trendelenburg position, with the operator standing between the patient's legs. A standard laparoscopic surgical technique with 5 to 6 trocars was used for both procedures.<br><br>For the case of GB surgical procedure, a simplified laparoscopic mini-GB was adopted and has been previously described.<br><br>Patients were advised to take a daily multivitamin tablet as a supplement. Iron supplement, vitamin B12 injection,	

	and blood transfusion were given only in symptomatic patients.	
**WHO PROVIDED**<br>For each category of intervention provider (e.g., psychologist, nursing assistant), describe their expertise, background and any specific training given.	Each potential participant was assessed by a multidisciplinary and integrated medical unit, with the aid of a team including a general physician, endocrinologist, psychiatrist, and dietician. A thorough assessment was performed of each patient's general condition and mental status, complications of obesity and diabetes mellitus, risk factors, and motivations for surgery. The endocrinologist and surgeon codetermined when a patient was ready for randomization.<br><br>"Patients were regularly followed up at the outpatient clinic by the aforementioned multidisciplinary team."<br><br>The surgical team performed both types of surgical procedures and had broad experience in both techniques.	
**HOW**<br>Describe the modes of delivery (e.g., face-to-face or by some other mechanism, such as internet or telephone) of the intervention and whether it was provided individually or in a group.	N/R	
**WHERE**<br>Describe the type(s) of location(s) where the intervention occurred, including any necessary infrastructure or relevant features.	The study was conducted in the Department of Surgery of the Min-Sheng General Hospital, National Taiwan University.<br><br>Follow-up was completed in "the outpatient clinic."	

**WHEN and HOW MUCH**<br>Describe the number of times the intervention was delivered and over what period of time including the number of sessions, their schedule, and their duration, intensity or dose.	12-month follow-up. Beyond assessment/measurement visits, no details on other intervention components.<br><br>BMI and HbA1c levels reported at 0,1,3,6 and 12 months.<br>Primary and secondary outcomes reported at 12 months including Insulin secretion	
**TAILORING**<br>If an intervention was planned to be personalized, titrated or adapted, then describe what, why, when, and how.	N/R	
**MODIFICATION**<br>If an intervention was modified during the study, describe the changes (what, why, when, and how).	N/R	
**HOW WELL**<br>Planned: If intervention adherence or fidelity was assessed, describe how and by whom, and if any strategies were used to maintain or improve fidelity, describe them.	Study adherence was assessed using attendance at appointments and completion of questionnaires.<br><br>A complication was defined as the occurrence of an unexpected medical event that made departure from the clinical pathway necessary. An early complication was defined as a complication that occurred within 30 days postoperatively. A major complication was defined as a complication that required interventional management and hospitalization for more than 14 days. Complications related to the operation occurred more than 30 days postoperatively, and complications that	

	required readmission were defined as late complications.	
Actual: If intervention adherence or fidelity was assessed, describe the extent to which the intervention was delivered as planned.	N/R	

Lin et al., 2022		
**TIDieR Tool Item**	**Main Paper**	**Other Paper(s)**
**BRIEF NAME**<br>Provide the name or a phrase that describes the intervention.	Loop versus Roux-en-Y duodenojejunal bypass with sleeve gastrectomy for type 2 diabetes: short-term outcomes of a single-center randomized controlled trial	
**WHY**<br>Describe any rationale, theory, or goal of the elements essential to the intervention.	In this study, we performed a single-center, randomized, controlled trial to compare loop DJB-SG with Roux-en-Y DJB-SG with different reconstruction methods but the same size of gastric sleeve and length of intestine bypassed in patients with T2D with preoperative body mass indexes (BMIs) of 27.5–40 kg/m2.<br><br>This study's main aim was to evaluate the effect of reconstruction method (loop versus Roux-en-Y) on T2D remission at 1-year follow-up.<br><br>Secondary aims were to compare the weight loss, major perioperative complications, co-morbidity resolution, medical cost, nutritional status, and gastrointestinal disorders at 1-year follow-up.	
**WHAT**<br>Materials: Describe any physical or informational materials used in the intervention, including those provided to participants or used in intervention delivery or in training of intervention providers.<br>Provide information on where the materials can be	N/R	

accessed (e.g., online appendix, URL).		
Procedures: Describe each of the procedures, activities, and/or processes used in the intervention, including any enabling or support activities.	All operations were performed laparoscopically. loop DJB-SG compared to Roux-en-Y DJB-SG with different reconstruction methods but the same size of gastric sleeve and length of intestine bypassed<br>All patients were asked to take proton-pump inhibitors for 4 weeks and supplement with the multivitamin and multi-mineral complex and protein powder for at least 1 year or longer if malnutrition exists.	
**WHO PROVIDED**<br>For each category of intervention provider (e.g., psychologist, nursing assistant), describe their expertise, background and any specific training given.	N/R	
**HOW**<br>Describe the modes of delivery (e.g., face-to-face or by some other mechanism, such as internet or telephone) of the intervention and whether it was provided individually or in a group.	N/R	
**WHERE**<br>Describe the type(s) of location(s) where the intervention occurred, including any necessary infrastructure or relevant features.	Single-center hospital	

**WHEN and HOW MUCH**<br>Describe the number of times the intervention was delivered and over what period of time including the number of sessions, their schedule, and their duration, intensity or dose.	1 year follow-up (post-intervention outcomes)<br><br>No details on other intervention sessions/visits or outcome measurement timepoints.	
**TAILORING**<br>If an intervention was planned to be personalized, titrated or adapted, then describe what, why, when, and how.	Meanwhile, 2 patients undergoing loop DJB-SG received synchronous hiatal hernia repair by simple cruroplasty.	
**MODIFICATION**<br>If an intervention was modified during the study, describe the changes (what, why, when, and how).	N/R	
**HOW WELL**<br>Planned: If intervention adherence or fidelity was assessed, describe how and by whom, and if any strategies were used to maintain or improve fidelity, describe them.	N/R	
Actual: If intervention adherence or fidelity was assessed, describe the extent to which the intervention was delivered as planned.	N/R	

Mingrone et al., 2012		
**TIDieR Tool Item**	**Main Paper**	**Other Paper(s)**
**BRIEF NAME**<br>Provide the name or a phrase that describes the intervention.	Bariatric surgery versus conventional medical therapy for type 2 diabetes	
**WHY**<br>Describe any rationale, theory, or goal of the elements essential to the intervention.	To compare the efficacy of two types of bariatric surgery (gastric bypass and biliopancreatic diversion) with conventional medical therapy in severely obese patients with type 2 diabetes.<br><br>Single-center, nonblinded, randomized, controlled trial.	
**WHAT**<br>Materials: Describe any physical or informational materials used in the intervention, including those provided to participants or used in intervention delivery or in training of intervention providers.<br>Provide information on where the materials can be accessed (e.g., online appendix, URL).	N/R	
Procedures: Describe each of the procedures, activities, and/or processes used in the intervention, including any enabling or support activities.	Multidisciplinary team evaluation.<br><br>Medical therapy was adjusted according to the seven-point glycemic profile during the first 3 months and according to glycated hemoglobin levels thereafter. Discontinuation of medical therapy was considered in cases of normalization of the	

	glycemic profile, glycated hemoglobin levels, or both. Daily multivitamin and mineral supplementation were prescribed to the surgical groups; patients undergoing biliopancreatic diversion received additional vitamin D and calcium supplementation.<br><br>Surgery: Gastric bypass or biliopancreatic diversion. A detailed description of the surgical procedures is provided in the Supplementary Appendix.	
**WHO PROVIDED**<br>For each category of intervention provider (e.g., psychologist, nursing assistant), describe their expertise, background and any specific training given.	Multidisciplinary team (including a diabetologist, a dietitian, and a nurse) conducted evaluations<br><br>Two teams of bariatric surgeons, one with expertise in laparoscopic gastric bypass and the other with expertise in open biliopancreatic diversion, performed the procedures.	Programs for diet and lifestyle modifications were designed and administered by experienced diabetologists and dietitians (all with more than 15 years of clinical practice)
**HOW**<br>Describe the modes of delivery (e.g., face-to-face or by some other mechanism, such as internet or telephone) of the intervention and whether it was provided individually or in a group.	N/R	
**WHERE**<br>Describe the type(s) of location(s) where the intervention occurred, including any necessary infrastructure or relevant features.	Day hospital (Tertiary)	
**WHEN and HOW MUCH**	N/R	N/R

Describe the number of times the intervention was delivered and over what period of time including the number of sessions, their schedule, and their duration, intensity or dose.	No visits or details beyond surgery and outcome measurement appointments - At baseline and at 1, 3, 6, 9, 12, and 24 months after surgery.	Study participants had visits at baseline and at months 1, 3, 6, 9, and 12, and then every 6 months until month 60, or more often, as clinically necessary for diabetes control.
**TAILORING** If an intervention was planned to be personalized, titrated or adapted, then describe what, why, when, and how.	Medical therapy was adjusted according to the seven-point glycemic profile e during the first 3 months and according to glycated hemoglobin levels thereafter. Discontinuation of medical therapy was considered in cases of normalization of the glycemic profile, glycated hemoglobin levels, or both.	Diet and lifestyle modification and dosage of glucose lowering drugs, insulin, and glucagon-like peptide-1 (GLP-1) analogues were optimized on an individual basis with the aim of reaching adequate glycemic control (HbA1c <7·0% [<53·0 mmol/mol]) in all groups and as per standard diabetes guidelines.
**MODIFICATION** If an intervention was modified during the study, describe the changes (what, why, when, and how).	N/R	Medical therapy options were updated throughout the 10-year period to reflect the most current standards of care.
**HOW WELL** Planned: If intervention adherence or fidelity was assessed, describe how and by whom, and if any strategies were used to maintain or improve fidelity, describe them.	N/R	
Actual: If intervention adherence or fidelity was assessed,	N/R	All patients complied with follow-up visits and self-reported compliance with the drug regimen.

describe the extent to which the intervention was delivered as planned.		

Moriconi et al., 2022		
**TIDieR Tool Item**	**Main Paper**	**Other Paper(s)**
**BRIEF NAME**<br>Provide the name or a phrase that describes the intervention.	Predicting diabetes relapse after Roux en-Y Gastric Bypass, 10-year follow up.	
**WHY**<br>Describe any rationale, theory, or goal of the elements essential to the intervention.	Bariatric surgery is considered an effective treatment for morbid obesity and is a useful adjunct in the treatment of type 2 diabetes mellitus (T2DM) in subjects with severe obesity. The exact mechanisms behind diabetes remission after bariatric surgery are not completely understood, but improvement in insulin sensitivity and ß-cell function as well as changes in the entero-insular axis appear to play an important role.<br><br>This study's aim was to determine the impact of RYGB on T2DM over 10-year follow-up, focusing on the incidence of T2DM relapse after the initial remission to identify potential predictive factors.	
**WHAT**<br>Materials: Describe any physical or informational materials used in the intervention, including those provided to participants or used in intervention delivery or in training of intervention providers.<br>Provide information on where the materials can be	N/R	

accessed (e.g., online appendix, URL).		
Procedures: Describe each of the procedures, activities, and/or processes used in the intervention, including any enabling or support activities.	One hundred twenty-seven subjects with morbid obesity and T2DMattending the outpatient clinic of the Internal Medicine Unit of the Azienda Ospedaliero-Universitaria Pisana (Pisa, Italy), were enrolled between 2008 and 2009 to take part in a single-center, open, 10-year prospective study. Of them, 96 patients underwent RYGB and 31refused to undergo bariatric surgery and were recruited into a medical intensive therapy program (MT).<br><br>In the MT group patients were treated by oral anti-hyperglycemic agents and/or insulin based on individual characteristics with the aim of reaching HbA1c levels < 6.5%. Patients received nutritional counseling and a low-calorie diet tailored to achieve a weight loss > 10% of initial body weight.<br><br>Patients in the surgery group were admitted to our ward two days before surgery and returned three days after the intervention for another three days, in order to check glucose control and adjust drug therapy. On the day of surgery, patients with a fasting plasma glucose concentration >8.0 mmol/l were started on an insulin infusion adjusted to maintain fasting plasma glucose between 6.0 and 8.0 mmol/l during surgery and for the following day, until they started eating again. Before surgery, 27 patients were on insulin therapy, 20 patients were on sulfonylureas plus metformin, and 58 patients took	

	metformin alone. A small group of 8 patients had sulfonylureas alone, due to intolerance to metformin, or a combination of metformin and exenatide.	
**WHO PROVIDED**<br>For each category of intervention provider (e.g., psychologist, nursing assistant), describe their expertise, background and any specific training given.	Before surgery, patients were evaluated by a multidisciplinary team, including a surgeon, an anesthesiologist, a diabetologist, a dietician, a psychologist and a psychiatrist.<br><br>N/R who completed each aspect of the intervention including surgery.	
**HOW**<br>Describe the modes of delivery (e.g., face-to-face or by some other mechanism, such as internet or telephone) of the intervention and whether it was provided individually or in a group.	Face-to-face	
**WHERE**<br>Describe the type(s) of location(s) where the intervention occurred, including any necessary infrastructure or relevant features.	Checked into internal medicine unit at 45 days post-op.<br>"Internal Medicine Unit of the Azienda Ospedaliero-Universitaria Pisana (Pisa, Italy)"<br><br>N/R where the operation occurred.	
**WHEN and HOW MUCH**<br>Describe the number of times the intervention was delivered and over what period of time including the number of sessions, their schedule, and their	"All patients were checked in our outpatient clinic of the Internal Medicine Unit at 45 days, 3, 6, and 12 months, and then every six months for the following years. At each visit, physical examination, weight control and laboratory tests were performed; furthermore, all patients underwent dietary assessment conducted by our team (using the EPIC Alimentary	

duration, intensity or dose.	Questionnaire). Blood samples included blood count, glycemia, HbA1c, lipid profile, kidney and liver function, electrolytes, urinalysis and albumin-to-creatinine ratio.<br><br>Admitted 3 days pre-op. Followed up in clinic starting day 45 and as stated above.<br><br>Intervention (operation) was delivered once and follow up occurred accordingly.<br><br>"Patients in the surgery group were admit- ted to our ward two days before surgery and returned three days after the intervention for another three days, in order to check glucose control and adjust drug therapy."	
**TAILORING**<br>If an intervention was planned to be personalized, titrated or adapted, then describe what, why, when, and how.	Patients received nutritional counseling and a low-calorie diet tailored to achieve a weight loss > 10% of initial body weight.<br><br>In both group, medical therapy was adjusted according to the metabolic pro- file, and discontinuation of medical therapy was considered in case of glycemic normalization."<br><br>Diet and medications were tailored.	
**MODIFICATION**<br>If an intervention was modified during the study, describe the changes (what, why, when, and how).	N/R	
**HOW WELL**<br>Planned: If intervention adherence or fidelity was assessed,	N/R	

describe how and by whom, and if any strategies were used to maintain or improve fidelity, describe them.		
Actual: If intervention adherence or fidelity was assessed, describe the extent to which the intervention was delivered as planned.	N/R	

Murphy et al., 2018		
**TIDieR Tool Item**	**Main Paper**	**Other Paper(s)**
**BRIEF NAME**<br>Provide the name or a phrase that describes the intervention.	Laparoscopic Sleeve Gastrectomy Versus Banded Roux-en-Y Gastric Bypass for Diabetes and Obesity.	
**WHY**<br>Describe any rationale, theory, or goal of the elements essential to the intervention.	Compared the effectiveness of (LSG) versus SR-LRYGB among patients with T2D and morbid obesity.<br><br>Prospective, randomized, parallel, 2-arm, blinded clinical trial conducted in a single Auckland (New Zealand) centre.<br><br>The primary outcome was T2D remission defined by different HbA1c thresholds at 1 year. Secondary outcomes included weight loss, quality of life, anxiety and depressive symptoms, postoperative complications and mortality.	
**WHAT**<br>Materials: Describe any physical or informational materials used in the intervention, including those provided to participants or used in intervention delivery or in training of intervention providers. Provide information on where the materials can be accessed (e.g., online appendix, URL).	N/R	
Procedures: Describe each of the procedures, activities, and/or processes used in the intervention, including any enabling or support activities.	This was a single-centre, prospective, randomized, parallel 2-arm, double-blind (patient and assessor) clinical trial conducted in Auckland, New Zealand, involving 114 obese patients with T2D who were randomized 1:1 to LSG or SR-LRYGB (both routine procedures at our institution).	All participants were prescribed a very-low-calorie diet with three servings of OPTIFAST (Nestle, Vevey, Switzerland) containing approximately 150 cal plus vegetables for 2 weeks before surgery (2023).

	All pharmacological agents for diabetes were<br>discontinued following surgery and subject to endocrinologist review before discharge. Metabolic medications were commenced post-operatively per a standard medical review protocol by endocrinologists blinded to treatment allocation. Generally, Metformin was restarted first, followed by adding sulfonylurea, then basal insulin, or premixed insulin, depending on the pattern of prandial hyperglycemia.	All patients were prescribed a twice-daily multivitamin (Centrum Plus; Pfizer; New Zealand) containing 200 mg elemental calcium and 600 IU vitamin D3 post-operatively (2023).
**WHO PROVIDED**<br>For each category of intervention provider (e.g., psychologist, nursing assistant), describe their expertise, background and any specific training given.	Endocrinologist monitored medications/pharmacological agents	Operations were performed by bariatric surgical fellows under the direct supervision of an experienced bariatric surgeon (2023).<br><br>Surgical team (2023).
**HOW**<br>Describe the modes of delivery (e.g., face-to-face or by some other mechanism, such as internet or telephone) of the intervention and whether it was provided individually or in a group.	N/R	
**WHERE**<br>Describe the type(s) of location(s) where the intervention occurred, including any necessary infrastructure or relevant features.	Tertiary hospital	
**WHEN and HOW MUCH**<br>Describe the number of times the intervention	N/R beyond surgery procedures and follow-up outcome measurements	All patients were reviewed by an endocrinologist up to 5 years post-operatively for adjustment of metabolic

was delivered and over what period of time including the number of sessions, their schedule, and their duration, intensity or dose.		medications according to a prespecified adjustment protocol and following this management of diabetic medications were transferred to general practitioners. Patients were offered follow-up appointments with the surgical team at yearly intervals for symptom review and data collection. Laboratory and medication dispensing data were also collected from an online medical record database analysis and accepted if within a window of ±6 months from the 7-year post-operative anniversary date.
**TAILORING** If an intervention was planned to be personalized, titrated or adapted, then describe what, why, when, and how.	N/R	All patients were reviewed by an endocrinologist up to 5 years post-operatively for adjustment of metabolic medications according to a prespecified adjustment protocol and following this management of diabetic medications was transferred to general practitioners.
**MODIFICATION** If an intervention was modified during the course of the study, describe the changes (what, why, when, and how).		Data collection for secondary outcomes presented in previous iterations of this study, including blood pressure and quality-of-life surveys, was limited due to the COVID-19 pandemic and the inability to see patients in person at the 7-year follow-up due to regional lockdowns. The lack of collected data for these parameters meant that we could not meaningfully analyze differences between groups, so the decision was

		made to exclude them from analysis and reporting at this timepoint (2023).
**HOW WELL**<br>Planned: If intervention adherence or fidelity was assessed, describe how and by whom, and if any strategies were used to maintain or improve fidelity, describe them.	N/R	
Actual: If intervention adherence or fidelity was assessed, describe the extent to which the intervention was delivered as planned.	N/R	

Parikh et al., 2014		
**TIDieR Tool Item**	**Main Paper**	**Other Paper(s)**
**BRIEF NAME**<br>Provide the name or a phrase that describes the intervention.	Bariatric surgery vs. intensive medical weight management on diabetes remission in type 2 diabetic patients who do NOT meet NIH criteria for surgery.	
**WHY**<br>Describe any rationale, theory, or goal of the elements essential to the intervention.	To compare bariatric surgery vs. intensive medical weight management (MWM) in patients with type 2 diabetes (T2DM) who do not meet current NIH criteria for bariatric surgery.<br><br>57 patients with T2DM and BMI 30–35 who otherwise met the criteria for bariatric surgery were randomized to MWM vs. surgery (bypass, sleeve or band, based on patient preference). The primary outcomes assessed at 6 months were changes in insulin resistance (HOMA-IR) and diabetes remission. Secondary outcomes included changes in HbA1c, weight, and sRAGE.	
**WHAT**<br>Materials: Describe any physical or informational materials used in the intervention, including those provided to participants or used in intervention delivery or in training of intervention providers.<br>Provide information on where the materials can be accessed (e.g., online appendix, URL).	Postoperative dietary guidelines were based on the ASMBS Allied Health Nutritional Guidelines for the Surgical Weight Loss Patient.<br><br>https://www.soard.org/article/S1550-7289%2808%2900163-9/pdf	

Procedures:<br>Describe each of the procedures, activities, and/or processes used in the intervention, including any enabling or support activities.	Patients randomized to surgery underwent bypass, band or sleeve gastrectomy based on patient preference (drawing upon information learned in the monthly bariatric surgery information seminar and during the surgeon consultation). All patients underwent a thorough evaluation by a surgeon, internist, nutritionist, and psychologist and then completed a liquid protein diet for two weeks before surgery to decrease hepatomegaly.<br><br>Post-surgery: Patients were maintained on clear liquids for 48 hours postoperatively, then advanced to a full liquid diet (including low-fat, low sugar, protein-rich shakes) for 2 weeks, followed by a pureed diet for 2 weeks and then transitioned to a regular diet.<br>Band adjustments were done percutaneously in the clinic according to a commonly used algorithm based on hunger and weight loss.	
**WHO PROVIDED**<br>For each category of intervention provider (e.g., psychologist, nursing assistant), describe their expertise, background and any specific training given.	Surgeon, internist, nutritionist, and psychologist	
**HOW**<br>Describe the modes of delivery (e.g., face-to-face or by some other mechanism, such as internet or telephone) of the intervention and	N/R	

whether it was provided individually or in a group.		
**WHERE**<br>Describe the type(s) of location(s) where the intervention occurred, including any necessary infrastructure or relevant features.	Clinic	
**WHEN and HOW MUCH**<br>Describe the number of times the intervention was delivered and over what period of time including the number of sessions, their schedule, and their duration, intensity or dose.	Patients were seen postoperatively at 2 weeks, 4 weeks and then monthly for the study. Total duration of 6 months.	
**TAILORING**<br>If an intervention was planned to be personalized, titrated or adapted, then describe what, why, when, and how.	Surgery type was patient preference (bypass, band or sleeve gastrectomy).<br><br>Post-operative nutrition was based on adaptive guidelines (ASMBS Allied Health Nutritional Guidelines for the Surgical Weight Loss Patient).<br><br>Band adjustments were done percutaneously in the clinic according to a commonly used algorithm based on hunger and weight loss.	
**MODIFICATION**<br>If an intervention was modified during the study, describe the changes (what, why, when, and how).	N/R	
**HOW WELL**	N/R	

Planned: If intervention adherence or fidelity was assessed, describe how and by whom, and if any strategies were used to maintain or improve fidelity, describe them.		
Actual: If intervention adherence or fidelity was assessed, describe the extent to which the intervention was delivered as planned.	N/R	

Singh et al., 2023		
**TIDieR Tool Item**	**Main Paper**	**Other Paper(s)**
**BRIEF NAME**<br>Provide the name or a phrase that describes the intervention.	One Anastomosis Gastric Bypass (OAGB) vs Roux en Y Gastric Bypass (RYGB) for Remission of T2DM in Patients with Morbid Obesity: A Randomized Controlled Trial	
**WHY**<br>Describe any rationale, theory, or goal of the elements essential to the intervention.	This randomized study was designed to compare OAGB and RYGB with remission of T2DM as the primary outcome.	
**WHAT**<br>Materials: Describe any physical or informational materials used in the intervention, including those provided to participants or used in intervention delivery or in training of intervention providers.<br>Provide information on where the materials can be accessed (e.g., online appendix, URL).	N/R	
Procedures: Describe each of the procedures, activities, and/or processes used in the intervention, including any enabling or support activities.	All the procedures were performed laparoscopically. For OAGB, a long gastric tube based on lesser curvature was fashioned over a 38 Fr bougie with a biliopancreatic limb length of 200 cm.<br><br>Upper gastrointestinal endoscopy (UGIE) was performed at 1 year and 4 years following surgery.	

	Serum total protein, albumin, vitamin D, vitamin B 12, intact PTH, folate, and iron levels were measured at 3, 6, and 12 months postoperatively and then yearly.	
**WHO PROVIDED**<br>For each category of intervention provider (e.g., psychologist, nursing assistant), describe their expertise, background and any specific training given.	A single surgeon.	
**HOW**<br>Describe the modes of delivery (e.g., face-to-face or by some other mechanism, such as internet or telephone) of the intervention and whether it was provided individually or in a group.	N/R	
**WHERE**<br>Describe the type(s) of location(s) where the intervention occurred, including any necessary infrastructure or relevant features.	Tertiary care academic hospital	
**WHEN and HOW MUCH**<br>Describe the number of times the intervention was delivered and over what period of time including the number of sessions, their schedule, and their	N/R beyond surgery and outcome timepoint visits<br><br>complete remission and improvement rates of type 2 diabetes mellitus following OAGB and RYGB at 6 months, 12 months, and yearly thereafter.	

duration, intensity or dose.	Upper gastrointestinal endoscopy (UGIE) was performed at 1 year and 4 years following surgery.<br><br>Serum total protein, albumin, vitamin D, vitamin B 12, intact PTH, folate, and iron levels were measured at 3, 6, and 12 months postoperatively and then yearly.	
**TAILORING**<br>If an intervention was planned to be personalized, titrated or adapted, then describe what, why, when, and how.	N/R	
**MODIFICATION**<br>If an intervention was modified during the study, describe the changes (what, why, when, and how).	N/R	
**HOW WELL**<br>Planned: If intervention adherence or fidelity was assessed, describe how and by whom, and if any strategies were used to maintain or improve fidelity, describe them.	N/R	
Actual: If intervention adherence or fidelity was assessed, describe the extent to which the intervention was delivered as planned.	N/R	

Techagumpuch et al., 2019		
**TIDieR Tool Item**	**Main Paper**	**Other Paper(s)**
**BRIEF NAME**<br>Provide the name or a phrase that describes the intervention.	Two Years Outcome in Diabetes Control after Bariatric Surgery Comparison between Laparoscopic Sleeve Gastrectomy and Laparoscopic Roux-En-Y Gastric Bypass	
**WHY**<br>Describe any rationale, theory, or goal of the elements essential to the intervention.	The present study was conducted to compare the effectiveness of diabetic control at two years after gastric bypass surgery and sleeve gastrectomy among Thai patients.	
**WHAT**<br>Materials: Describe any physical or informational materials used in the intervention, including those provided to participants or used in intervention delivery or in training of intervention providers.<br>Provide information on where the materials can be accessed (e.g., online appendix, URL).	N/R	
Procedures: Describe each of the procedures, activities, and/or processes used in the intervention, including any enabling or support activities.	All operations were performed laparoscopically by the same surgeon and by the same surgical team. The patients were operated under general anesthesia in a reverse Trendelenburg position. A standard laparoscopic technique with four to five ports was used for both procedures.<br><br>For the sleeve gastrectomy, the procedure was started from mobilized greater curvature of stomach until	

	reach angle of His. The stomach was then incised using linear staple with 4 cm antral sparing and the staple line was continued to create the residual stomach size equally to Bougie 36 Fr.<br><br>After the operation, all patients were educated about nutrition, medication, and routine post- operative care. Patients with fasting plasma glucose (FPG) of more than 120 or HbA1c of more than 6% continued antiglycemic control medication according to ADA guideline for DM control (13). Follow-up was arranged at 2-week, 1-month, and every three months. At six-month and two-year after operation, the evaluation of FPG, HbA1c, and weight loss were performed by physician.	
**WHO PROVIDED**<br>For each category of intervention provider (e.g., psychologist, nursing assistant), describe their expertise, background and any specific training given.	All operations were performed laparoscopically by the same surgeon and by the same surgical team.<br><br>At six-month and two-year after operation, the evaluation of FPG, HbA1c, and weight loss were performed by physician.	
**HOW**<br>Describe the modes of delivery (e.g., face-to-face or by some other mechanism, such as internet or telephone) of the intervention and whether it was provided individually or in a group.	N/R.<br><br>The article states that patients participating in the research must have the "ability to follow-up for glycemic control at the authors' hospital."	
**WHERE**<br>Describe the type(s) of location(s) where the intervention	The study was conducted in the Department of Surgery of the King Chulalongkorn Memorial University Hospital.	

occurred, including any necessary infrastructure or relevant features.	Tertiary	
**WHEN and HOW MUCH**<br>Describe the number of times the intervention was delivered and over what period of time including the number of sessions, their schedule, and their duration, intensity or dose.	Follow-up was arranged at 2-week, 1-month, and every three months. At six-month and two-year after operation, the evaluation of FPG, HbA1c, and weight loss were performed by physician.<br><br>All patients received follow-up at two years.	
**TAILORING**<br>If an intervention was planned to be personalized, titrated or adapted, then describe what, why, when, and how.	N/R	
**MODIFICATION**<br>If an intervention was modified during the study, describe the changes (what, why, when, and how).	N/R	
**HOW WELL**<br>Planned: If intervention adherence or fidelity was assessed, describe how and by whom, and if any strategies were used to maintain or improve fidelity, describe them.	N/R	
Actual: If intervention adherence or fidelity was assessed,	N/R	

describe the extent to which the intervention was delivered as planned.		

Wallenius et al., 2020		
**TIDieR Tool Item**	**Main Paper**	**Other Paper(s)**
**BRIEF NAME**<br>Provide the name or a phrase that describes the intervention.	Sleeve gastrectomy and Roux-en-Y gastric bypass in the treatment of type 2 diabetes.	
**WHY**<br>Describe any rationale, theory, or goal of the elements essential to the intervention.	The aim of the present study was to compare diabetes remission rates in obese T2D patients (body mass index [BMI] 35–50) undergoing RYGB or SG. We hypothesized in line with the results of earlier studies that RYGB would be superior to SG in diabetes remission rates.	
**WHAT**<br>Materials: Describe any physical or informational materials used in the intervention, including those provided to participants or used in intervention delivery or in training of intervention providers.<br>Provide information on where the materials can be accessed (e.g., online appendix, URL).	N/R	
Procedures: Describe each of the procedures, activities, and/or processes used in the intervention, including any enabling or support activities.	All operations were performed by laparoscopy. For<br>RYGB, a 15- to 30-mL proximal gastric pouch was constructed using a 45-mm linear stapler. The biliopancreatic limb of approximately 50 cm was measured by hand-overhand along the mesenteric border. An antecolic, antegastric, end-to-side gastrojejunostomy was formed with a linear stapler combined with	

	hand-suturing of the remaining opening. A 120-cm alimentary limb was constructed as described previously [11]. The gastroenteroanastomosis and gastric pouch staple lines were tested intraoperatively for leakage by placing a gastrotube in the pouch and infusing methylene blue solution. Mesenteric defects were closed by clips or by suturing in all patients.<br><br>A standardized supplementation regimen of micronutrients was prescribed to all patients including daily intake of 100 to 200 mg iron, 1 g calcium, 800 to 1600 U of vitamin D3, 1 mg vitamin B12, and a multivitamin supplementation.<br><br>All diabetes medications before and after surgery were adjusted and prescribed by their usual physicians, and therefore reflect the real-world practices used mainly at their primary healthcare centers.	
**WHO PROVIDED**<br>For each category of intervention provider (e.g., psychologist, nursing assistant), describe their expertise, background and any specific training given.	Surgeon<br><br>All diabetes medications before and after surgery were adjusted and prescribed by their usual physicians, and therefore reflect the real-world practices used mainly at their primary healthcare centers.	
**HOW**<br>Describe the modes of delivery (e.g., face-to-face or by some other mechanism, such as internet or telephone) of the intervention and	N/R	

whether it was provided individually or in a group.		
**WHERE**<br>Describe the type(s) of location(s) where the intervention occurred, including any necessary infrastructure or relevant features.	4 bariatric surgery centers in Sweden (Sahlgrenska University Hospital/ SahlgrenskaandSahlgrenska/ O€strainGothen- burg, Skaraborg Hospital in Sk€ovde, and Ersta Hospital in Stockholm).	
**WHEN and HOW MUCH**<br>Describe the number of times the intervention was delivered and over what period of time including the number of sessions, their schedule, and their duration, intensity or dose.	Follow-up visits at the study centers were scheduled at 6 weeks and at 6, 12, and 24 months after surgery. Preoperatively, demographic data and the patients' medical history was collected. On all visits, including preoperatively, BMI (weight, height), waist circumference, blood pressure, and any ongoing medications for treatment of diabetes, dyslipidemia, or hypertension were recorded. Blood samples were collected for analysis of complete blood count, C-reactive protein, glycemic control (glycosylated hemoglobin [HbA1C], fasting glucose, insulin), lipid profiles, liver function tests, electrolytes and creatinine, iron, calcium, and vitamin D.<br><br>The primary endpoint was remission of T2D at 1 year after surgery with a planned follow-up time of 5 years.	
**TAILORING**<br>If an intervention was planned to be personalized, titrated or adapted, then describe what, why, when, and how.	N/R	
**MODIFICATION**<br>If an intervention was modified during the study, describe	One control patient, (which was not considered a surgical complication) had to be converted to RYGB because of a thick and inflamed gastric antrum,	

the changes (what, why, when, and how).	where both the staple and hand-sewn sutures lacerated the tissue, and therefore this patient could not be operated by protocol but had to be converted to a RYGB, with resection of the remnant stomach.	
**HOW WELL** Planned: If intervention adherence or fidelity was assessed, describe how and by whom, and if any strategies were used to maintain or improve fidelity, describe them.	N/R	
Actual: If intervention adherence or fidelity was assessed, describe the extent to which the intervention was delivered as planned.	N/R	

Wentworth et al., 2014		
**TIDieR Tool Item**	**Main Paper**	**Other Paper(s)**
**BRIEF NAME**<br>Provide the name or a phrase that describes the intervention.	Multidisciplinary diabetes care with and without bariatric surgery in overweight people.	Long-term impact of weight loss for people who are overweight<br>but not obese, and with type 2 diabetes (2022).
**WHY**<br>Describe any rationale, theory, or goal of the elements essential to the intervention.	Aimed to identify whether laparoscopic adjustable gastric band surgery can improve glucose control in people with type 2 diabetes who were overweight but not obese.<br><br>An open-label, parallel-group, randomized controlled trial.	
**WHAT**<br>Materials: Describe any physical or informational materials used in the intervention, including those provided to participants or used in intervention delivery or in training of intervention providers.<br>Provide information on where the materials can be accessed (e.g., online appendix, URL).	N/R	
Procedures: Describe each of the procedures, activities, and/or processes used in the intervention, including any enabling or support activities.	Multidisciplinary diabetes care was based on guidelines from the American Diabetes Association and delivered in Melbourne by an endocrinologist.<br><br>Consultations with a dietitian and diabetes educator. Additional consultations with the care team if deemed necessary.	

	Participants were advised to do at least 150 min of moderate-intensity physical activity each week. HbA1c was targeted to less than 7·0% (54 mmol/mol) with use of drugs available through the Australian Pharmaceutical Benefit Scheme. Metformin was recommended to all participants unless it was not tolerated or if results of an oral glucose tolerance test were normal at 1 year. Sitagliptin or exenatide were commenced if HbA1c was greater than 7·0% despite metformin or sulfonylurea therapy. Insulin was commenced after sitagliptin and exenatide were trialed and shown to be ineffective at lowering HbA1c to less than 7·0%. We were able to combine exenatide with insulin, but usually increased the insulin dose and did not prescribe exenatide if HbA1cwas greater than 7·0% despite insulin therapy. Blood pressure was targeted to less than 120/80 mm Hg in participants with albuminuria or a history of cardiovascular disease, and to less than 130/80 mm Hg for others. Antihypertensive drug therapy was intensified if these targets were not achieved after 3 months of lifestyle change. Anti-platelet therapy and statins were prescribed to all participants with a history of cardiovascular disease and to those older than 40 years who had an additional cardiovascular risk factor. Annual ophthalmology review was arranged through the family physician. Tailored calorie-restricted diet. Multivitamin supplements were recommended to all participants in the gastric banding group. The	

	sessions with a diabetes educator focused on diabetes self-management, including blood glucose monitoring and sick-day management (i.e., patients are educated about the possibility of hyperglycemia or hypoglycemia if they develop an illness such as respiratory tract or bowel infections).<br><br>Patients in the gastric banding group underwent surgery within 3 months of randomization.<br><br>Aftercare to adjust the band and optimize eating and exercise behaviors.	
**WHO PROVIDED**<br>For each category of intervention provider (e.g., psychologist, nursing assistant), describe their expertise, background and any specific training given.	Surgeon, endocrinologist, physician, pathologist (biochemistry), dietitian, diabetes educator and study nurse. Members of care team (not identified). Ophthalmology assessment through family physician.	
**HOW**<br>Describe the modes of delivery (e.g., face-to-face or by some other mechanism, such as internet or telephone) of the intervention and whether it was provided individually or in a group.	Outpatient consultations. Mode N/R but individual face-to-face implied.	At 10-year follow-up: A single physician (JMW) interviewed all participants in person or by telephone and each subsequently attended a community optometrist or ophthalmologist for eye examination (2022).<br><br>COVID restrictions during the study required some participants to consult their GP for measurement of weight, waist circumference and blood pressure. Pathology endpoints were measured by accredited

		pathology providers in the community
**WHERE**<br>Describe the type(s) of location(s) where the intervention occurred, including any necessary infrastructure or relevant features.	Private hospital specializing in bariatric surgery (Tertiary)<br><br>Affiliated specialist bariatric center/clinic for follow-up (Secondary)<br><br>Lab/pathology clinic (secondary)	
**WHEN and HOW MUCH**<br>Describe the number of times the intervention was delivered and over what period of time including the number of sessions, their schedule, and their duration, intensity or dose.	Biochemistry and physician review was done every 3 months in year 1, and every 6 months in year 2. Consultations with a dietitian and a diabetes educator were arranged within 6 months in those in the laparoscopic adjustable gastric banding group.	Multidisciplinary diabetes care (MDC) or GB surgery combined with MDC for 5 years. MDC involved review by a diabetes specialist every 3-6 months and by a dietician and diabetes educator on two occasions in the first year and as required thereafter. Followed for 5 years, then discharged to receive community care and reassessed after 10 years (2022).
**TAILORING**<br>If an intervention was planned to be personalized, titrated or adapted, then describe what, why, when, and how.	Additional consultations with members of the care team were arranged if deemed necessary by either JMW or the participant. If HbA1c was greater than 7% in year 2 of the trial, endocrinology review was scheduled every 3 months.<br><br>Metformin was recommended to all participants unless it was not tolerated or if results of an oral glucose tolerance test were normal at 1 year.<br><br>Sitagliptin or exenatide were commenced if HbA1c was greater than 7·0% despite metformin or sulfonylurea therapy. Insulin was commenced after sitagliptin and exenatide were trialed and shown to	

	be ineffective at lowering HbA1c to less than 7·0%. We were able to combine exenatide with insulin, but usually increased the insulin dose and did not prescribe exenatide if HbA1c was greater than 7·0% despite insulin therapy. Blood pressure was targeted to less than 120/80 mm Hg in participants with albuminuria or a history of cardiovascular disease, and to less than 130/80 mm Hg for others. Antihypertensive drug therapy was intensified if these targets were not achieved after 3 months of lifestyle change.<br><br>Drugs were weaned if JMW (endo) judged that the participant would continue to meet treatment targets, which were reassessed within 3 months of this change.<br><br>The dietitian recommended a tailored calorie-restricted diet to all participants.<br><br>Aftercare to adjust the band and optimize eating and exercise behaviours was provided at The Centre for Bariatric Surgery (Melbourne, Australia) according to the management algorithms established by the centre.<br><br>Incidental hiatal hernia was repaired as part of the procedure.	
**MODIFICATION**<br>If an intervention was modified during the study, describe the changes (what, why, when, and how).	N/R	COVID restrictions during the study required some participants to consult their GP for measurement of weight, waist circumference and blood pressure.
**HOW WELL**	Attendance at consultations	

Planned: If intervention adherence or fidelity was assessed, describe how and by whom, and if any strategies were used to maintain or improve fidelity, describe them.		
Actual: If intervention adherence or fidelity was assessed, describe the extent to which the intervention was delivered as planned.	One participant in the intervention group failed to attend scheduled physician reviews. Nine participants in the intervention group (39%) did not consult with the dietitian.	

Yi et al., 2015		
**TIDieR Tool Item**	**Main Paper**	**Other Paper(s)**
**BRIEF NAME**<br>Provide the name or a phrase that describes the intervention.	Comparison of the effects of Roux-en-Y gastrojejunostomy and LRYGB with small stomach pouch on type 2 diabetes.	
**WHY**<br>Describe any rationale, theory, or goal of the elements essential to the intervention.	Both procedures (laparoscopic Roux-en-Y gastrojejunostomy and LRYGB with a small gastric pouch) were effective in T2DM.<br>However, controversy exists concerning which procedure is more suitable for Chinese T2DM patients. Therefore, we conducted a comparative study to assess the efficacy and side effects of 2 procedures.	
**WHAT**<br>Materials: Describe any physical or informational materials used in the intervention, including those provided to participants or used in intervention delivery or in training of intervention providers.<br>Provide information on where the materials can be accessed (e.g., online appendix, URL).	N/R	
Procedures: Describe each of the procedures, activities, and/or processes used in the intervention, including any	As part of a preoperative laboratory assessment, the patients enrolled in the study underwent baseline blood collection for measurements of FPG levels, PPG levels,<br>OGTT, OGT-C-peptide release (CPR), and HbA1c levels. Homoeostatic	

enabling or support activities.	model assessment (HOMA-IR) was also calculated using the standardized equation. In addition, BMI, the body fat rate, and waist circumference were measured.<br><br>All of the surgical procedures were performed at Third Xiangya Hospital by one surgeon (description of specific surgical procedures were provided).<br><br>The total time required for each procedure was approximately 3 hours.	
**WHO PROVIDED**<br>For each category of intervention provider (e.g., psychologist, nursing assistant), describe their expertise, background and any specific training given.	One single surgeon	
**HOW**<br>Describe the modes of delivery (e.g., face-to-face or by some other mechanism, such as internet or telephone) of the intervention and whether it was provided individually or in a group.	N/R	
**WHERE**<br>Describe the type(s) of location(s) where the intervention occurred, including any necessary infrastructure or relevant features.	Tertiary hospital	
**WHEN and HOW MUCH**	Outcomes were measured at baseline, 6 and 12 months after the surgery.	

Describe the number of times the intervention was delivered and over what period of time including the number of sessions, their schedule, and their duration, intensity or dose.		
**TAILORING**<br>If an intervention was planned to be personalized, titrated or adapted, then describe what, why, when, and how.	N/R	
**MODIFICATION**<br>If an intervention was modified during the study, describe the changes (what, why, when, and how).	N/R	
**HOW WELL**<br>Planned: If intervention adherence or fidelity was assessed, describe how and by whom, and if any strategies were used to maintain or improve fidelity, describe them.	N/R	
Actual: If intervention adherence or fidelity was assessed, describe the extent to which the intervention was delivered as planned.	N/R	
